# Shrimp oral immunotherapy outcomes in the phase 2 clinical trial: MOTIF

**DOI:** 10.3389/falgy.2025.1458131

**Published:** 2025-07-22

**Authors:** Shirley Y. Jiang, Shu Cao, Kristine Martinez, Reyna Sharma, Olivia Raeber, Andrea Fernandes, Dinara Bogetic, Abhinav Kaushik, Sheena Gupta, Monali Manohar, Holden T. Maeker, Andrew R. Chin, Andrew J. Long, Catherine Feight, Margaret Woch, Kari C. Nadeau, R. Sharon Chinthrajah, Sayantani B. Sindher

**Affiliations:** ^1^ Sean N Parker Center for Allergy and Asthma Research at Stanford University, Stanford University, Stanford, CA, United States; ^2^ Institute for Immunity, Transplantation and Infection, Stanford University School of Medicine, Stanford, CA, United States; ^3^ Department of Environmental Health, T.H. Chan School of Public Health, Harvard University, Boston, MA, United States

**Keywords:** shrimp, allergy, oral immunotherapy, clinical trial, double-blind placebo-controlled food challenge

## Abstract

**Introduction:**

Shrimp is a common but understudied food allergen with relatively high rates of emergency department visits. Here we report the shrimp OIT outcomes in the MOTIF (NCT03504774) clinical trial and discuss some of the challenges with performing this study.

**Methods:**

In this phase 2 clinical trial, 12 shrimp allergic participants aged 7–55 years (median age 21.5 years) were enrolled to receive shrimp OIT. Shrimp OIT was performed up to a maintenance dose of 1,000 mg shrimp protein by week 28 with desensitization to shrimp assessed by double-blind placebo-controlled food challenge at week 52 followed by switching to avoidance and assessing sustained unresponsiveness (SU) at week 58. The primary endpoint was the change in CD28 in CD4+ allergen specific (CD154+) T-cells at baseline and 52 weeks.

**Results:**

Shrimp OIT induced desensitization to a cumulative 4,043 mg shrimp protein in 58.3% (7/12) of the intention to treat and 87.5% (7/8) of the per protocol group after 52 weeks of shrimp OIT. Most shrimp OIT participants who remained in the study after desensitization (87.5%, 7/8) achieved SU. Although adverse events were common during shrimp OIT (75%), most were mild (Bock grade 1, 88%) and there were no severe (Bock grade 3+) reactions or use of epinephrine. No significant differences in CD28 expression were observed after shrimp OIT.

**Conclusions:**

Shrimp OIT is safe and effective for the treatment of shrimp allergy. Most participants were successful and achieved SU after 6 weeks of avoidance.

## Introduction

Food allergies are globally recognized as a growing concern due to their increasing incidence and significant impacts on both physical and psychosocial welfare (1–6). Individuals diagnosed with food allergy have increased anxiety and hesitation for events that are often common place for non-allergic individuals such as eating at restaurants, attending school, and social events (7). Due to the substantial impact food allergy has on quality of life, food allergy clinical trials are needed to characterize and identify potential therapeutic strategies such as oral immunotherapy (OIT) to alleviate this burden. However, while there are a wealth of studies on more common allergens such as peanut and milk (8–15), the efficacy and safety of therapies such as OIT in other allergens such as shrimp are poorly understood and have generally been limited to case series (16).

Shellfish are the most common food allergens in adults and among the top allergens in children, affecting 9.6 million and 4.3 million individuals respectively in the United States (17, 18). A Canadian study has suggested that among shellfish, shrimp is most often responsible for allergic reactions including anaphylaxis (19), highlighting the importance of shrimp allergy therapeutics. Here we report the shrimp OIT outcomes and challenges faced in a phase 2 OIT clinical trial at Stanford University [T Cell Reagent Research for Monitoring T Cells in Food Allergy (MOTIF), NCT03504774]. The main goals of the study were to characterize the change in T-cell signature in participants achieve desensitization and subsequent sustained unresponsiveness compared to those who don't. The primary endpoint of the MOTIF trial was the differences in the expression of CD28 in CD4+ allergen specific (CD154+) T-cells at baseline and 52 weeks, since several studies have suggested that differences in immune cell populations such as CD4+ T cells may drive OIT outcomes (20–22).

## Methods

### For MOTIF shrimp OIT clinical trial

MOTIF was a phase 2, single-allergen OIT study for cashew and shrimp at the Sean N. Parker Center for Allergy and Asthma Research at Stanford University (Stanford, CA, USA). The Institutional Review Board of Stanford University School of Medicine approved the single site protocol under IND 18892. The study was conducted in accordance with the current revision of the Declaration of Helsinki and with the International Conference for Harmonization Good Clinical Practice (GCP) regulations and guidelines. The results for the cashew OIT participants will be published separately (publication in review). Here we discuss the shrimp OIT outcomes from MOTIF.

Participants aged 7–55 years were recruited between July 2019 and December 2021, with a history of shrimp allergy as indicated by serum IgE to shrimp ≥0.35 kUA/L, and/or a skin prick test (SPT) to shrimp ≥3 mm compared to saline control. Inclusion criteria required dose-limiting symptoms at or before the 300 mg dosing level of food allergen (FA) protein during a screening double-blind placebo-controlled food challenge (DBPCFC). Each screening DBPCFC consisted of several escalating doses of 3, 10, 30, 100, and 300 mg of oat flour for placebo and shrimp protein in flour form based on PRACTALL dosing guidelines (23) concealed in an appropriate vehicle, such as applesauce or pudding, ingested by the participant every 15 min as tolerated. A cumulative dose of 443 mg for shrimp was chosen based on eligibility for clinical trials as determined by the Consortium of Food Allergy Research (24). Dose-limiting symptoms and severities were determined according to modified Bock criteria (25). SPTs were performed on the volar surface of the forearm or back using a positive histamine control, a negative saline control (both from Hollister-Stier), and allergen extracts from Greer®. Mean wheal diameter was measured after 15 min. The Greer® extract used consisted of a mixture of white, brown, and pink shrimp (*Litopaenaeus setiferus, Farfantepenaeus aztecus*, and *Farfantepenaeus dourarum*) and testing was compliant as part of eligibility for clinical trials. Shrimp-specific IgE and IgG4 levels were measured by ImmunoCAP® fluorescence enzyme immunoassay. Written consent from a parent was obtained from participants age 7–17 years. Written consent was obtained directly from all adult participants (18+ years of age). Participants with severe or uncontrolled asthma, a history of uncontrolled cardiovascular disease, eosinophilic gastrointestinal disease, and allergy to oats (used as control) were excluded from the study.

**Figure 1:**
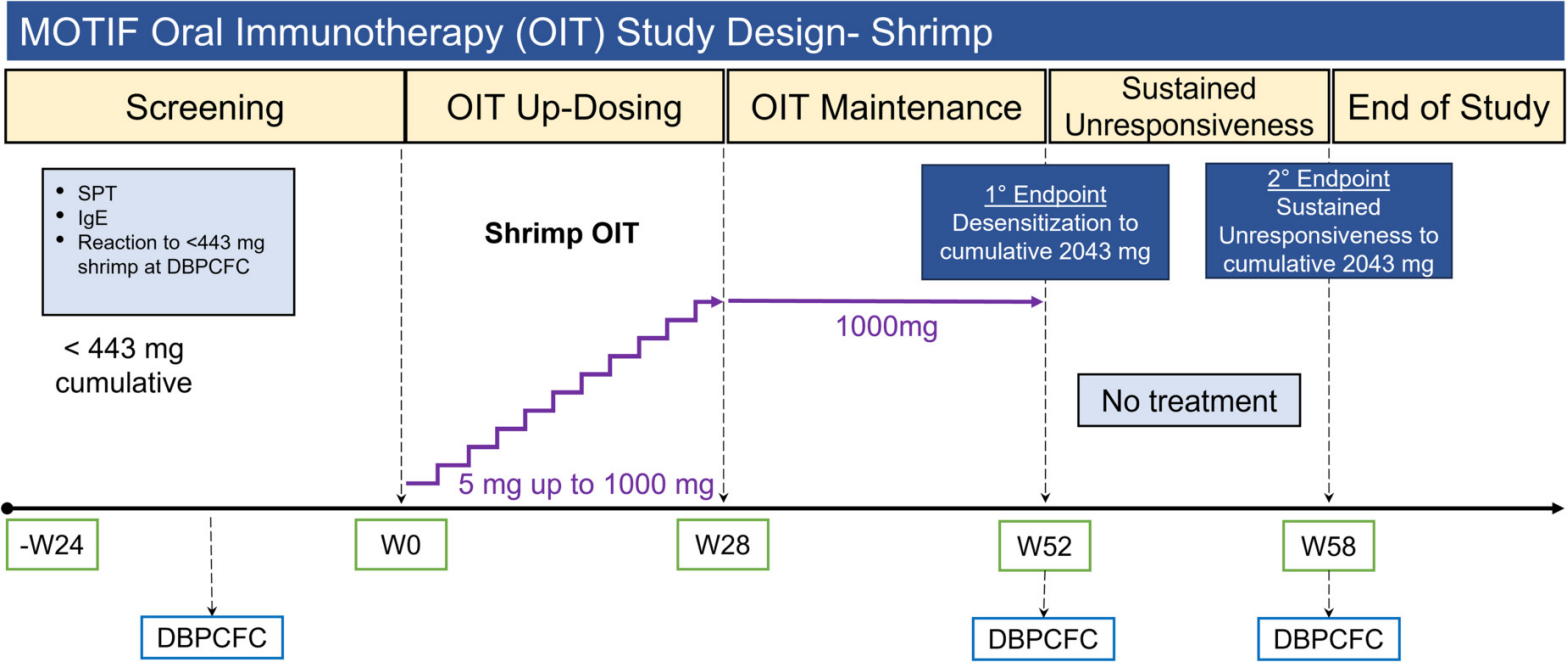
MOTIF study design for shrimp allergic participants. OIT, oral immunotherapy; SPT, skin prick test; IgE, immunoglobulin E; DBPCFC, double-blind placebo controlled food challenge; W#, week number.

Participants underwent an initial dose escalation day up to a maximum single dose of 5 mg shrimp protein (26, 27). The OIT dose was escalated every 2 weeks until they reached a maintenance dose of 1,000 mg shrimp protein (Supplementary Table S1). Participants continued their maintenance dose until week 52, after which they were transitioned to shrimp avoidance for an additional 6 weeks to assess for sustained unresponsiveness. DBPCFCs were performed at baseline, desensitization (week 52) and sustained unresponsiveness (week 58) time points (Figure 1) to a maximum cumulative dose of 4,043 mg shrimp protein using standard methodology according to validated guidelines (25, 28, 29).

Peripheral blood mononuclear cells (PBMCs) from baseline, week 52, and week 58 were thawed and re-suspended in RPMI with 5% heat-inactivated human AB serum and Penicillin-Streptomycin at a concentration of 3 × 10^6^ cells/ml in polypropylene FACS tubes. The PBMCs were rested overnight at 37°C and 5% CO2 followed by stimulation with 200 µg/ml of shrimp protein solution, derived from the flours used for DBPCFC, for 24 h. Unstimulated PBMCs served as controls. Brefeldin A (5 µg/ml; Biolegend, San Diego, CA) and Monensin (2 µm, Biolegend, San Diego, CA) were added to allergen-stimulated as well as unstimulated PBMCs for the last 4 h of incubation to inhibit vesicular transport of CD40l and CD69. At the end of the 24-h incubation, 120 µl of PBMC culture supernatant was collected for Luminex proteomic assays. and PBMCs were transferred into a 96-well, V-bottom plate. Harvested PBMCs were washed with CyFACS buffer (Dulbecco's PBS + 0.1% BSA + 0.2 M EDTA + 0.1% Sodium Azide) and stained with a 24-marker flow antibody panel (Supplementary Table S2). Data were acquired using BD Symphony A5™ cytometer and analyzed by manual gating. Live CD4+ cells overexpressing CD69 and CD40l in response to allergen stimulation were identified as allergen-reactive. For Luminex proteomics assays, the culture supernatants were probed using a 48-plex Cytokine/Chemokine Magnetic Bead Panel (HCYTA-60K-PX48, Millipore Sigma, Burlington, MA). Plasma aliquots were probed with the following Luminex panels: HCYTA-60K-PX48, HCP2MAG-62K-PX23, HSP1MAG- 63K-06, and HADCYMAG-61K-03 (Millipore Sigma).

### For shrimp screening

Due to the unexpected challenges in recruiting participants with DBPCFC-validated shrimp allergy, we evaluated shrimp sensitivity in research participants from September 2013 to December 2021 who underwent OFCs for shrimp as part of screening for several different clinical trials at Stanford University that included shrimp treatment options (IRB-34738). Included participants were initially screened for a clinical history that was suggestive of an IgE-mediated food allergy to shrimp. Participants with a prior history of reaction to shrimp requiring intubation or eliciting hypotension or moderate-to-severe asthma were excluded. During the initial screening visit for assessing eligibility for clinical research trials, SPT and IgE testing were performed at Stanford University, or results from prior testing at a physician's office within the past year were included. Positive sIgE was considered ≥0.35 kUA/L and positive shrimp SPT wheal was considered ≥3 mm. In cases with negative SPT and/or sIgE results, challenges were performed due to clinical history strongly suggestive of an IgE-mediated shrimp allergy. House dust mite (HDM) SPT to D. *farina*e and D. *pteronyssinus* were performed on a later subset of participants, to assess the potential of HDM sensitization in shrimp screen fails. All participants with a positive clinical history, regardless of the outcome of their shrimp sIgE level and SPT underwent an DBPCFC to shrimp.

### Statistical analysis

#### For MOTIF shrimp OIT clinical trial

The intent-to-treat (ITT) population was defined as all enrolled participants; the per-protocol (PP) population was defined as only individuals who remained in the study at each challenge endpoint. The clinical efficacy analyses were primarily performed in the ITT population and further summarized in PP population as secondary analysis. We tested the association between baseline characteristics and desensitization and SU using logistic regression adjusted for allergen. Participants who dropped out of the study were censored at their dropout date. The percentages of participants with any adverse events were compared descriptively across allergen groups. All analyses were conducted using two-sided tests where *p* < 0.05 was determined to be the cut-off for statistical significance.

#### For shrimp screening

Participants' demographic, clinical history and DBPCFC data were collected from our REDCap database. Demographic data included age at enrollment, sex, ethnicity, and race. Clinical history included a history of food allergy, asthma, allergic rhinitis and atopic dermatitis. These clinical diagnoses consisted of self-reported physician diagnoses. Continuous variables were summarized using median and range, categorical variables were summarized using frequency and percentage. The Kruskal–Wallis rank sum test or Fisher's exact test were used to test whether continuous and categorical variables were associated with food challenge outcome. All tests were two-sided and conducted at the 0.05 alpha level.

## Results

### MOTIF shrimp OIT clinical trial

**Figure 2:**
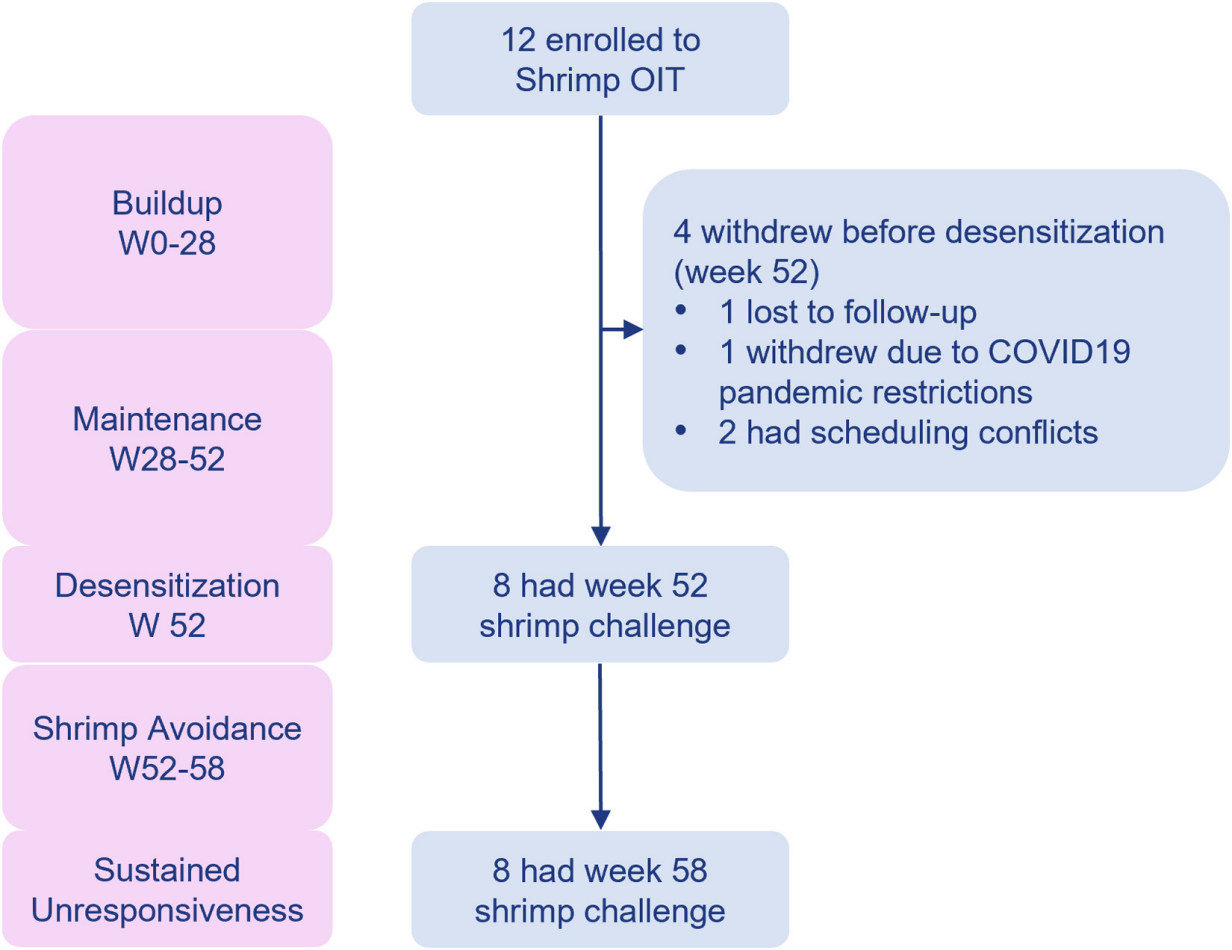
MOTIF consort diagram.

Of the 27 participants screened for shrimp OIT in the MOTIF study, 15 (55.6%) did not meet the eligibility criteria, resulting in the enrollment of 12 participants for shrimp OIT. Among the 12 enrolled participants, 4 withdrew before the desensitization time point (week 52, Figure 2). Withdrawal reasons included lost to follow-up, difficulties with COVID19 pandemic restrictions, and scheduling conflicts. Baseline clinical characteristics for the ITT population are summarized in Table 1. The median age at enrollment was 21.5 years (IQR 17.3–37.8 years). Most (83%) of the participants had a history of comorbid conditions including allergic rhinitis (75%), asthma (58.3%), and atopic dermatitis (50%). The median shrimp-specific IgE was 2.5 kUA/L (IQR 0.6–5.4) and the median cumulative tolerated dose (CTD) on screening DBPCFC was 143 mg (IQR 35–143) for shrimp. All shrimp allergic participants had demonstrated a systemic allergic reaction to shrimp in their medical history.

**Table 1:** Baseline demographics for shrimp allergic participants in the MOTIF study.

Shrimp allergic participants	*n* = 12
Age (years) median [IQR]	21.5 [16.3, 36]
Male	4 (33.3%)
Race	
• White	7 (58.3%)
• Asian	4 (33.3%)
• Multiracial	0 (0%)
• Native Hawaiian or Pacific Islander	1 (8.3%)
Hispanic or Latino	1 (8.3%)
Number of food allergies median [IQR]	3.5 [1, 10]
Asthma and allergy condition diagnosis	
• Any comorbid atopic condition	10 (83%)
• Allergic Rhinitis	9 (75%)
• Asthma	7 (58.3%)
• Atopic Dermatitis	6 (50%)
Baseline food challenge cumulative tolerated dose (CTD) (mg) median [IQR]	143 [35.5, 143]
Baseline Total IgE (IU/ml) median [IQR]	443 [143, 443]
Specific IgE (IU/ml) median [IQR]	2.5 [0.6, 5.4]
SPT Average wheal (mm) median [IQR]	4.8 [4, 8]

**Figure 3:**
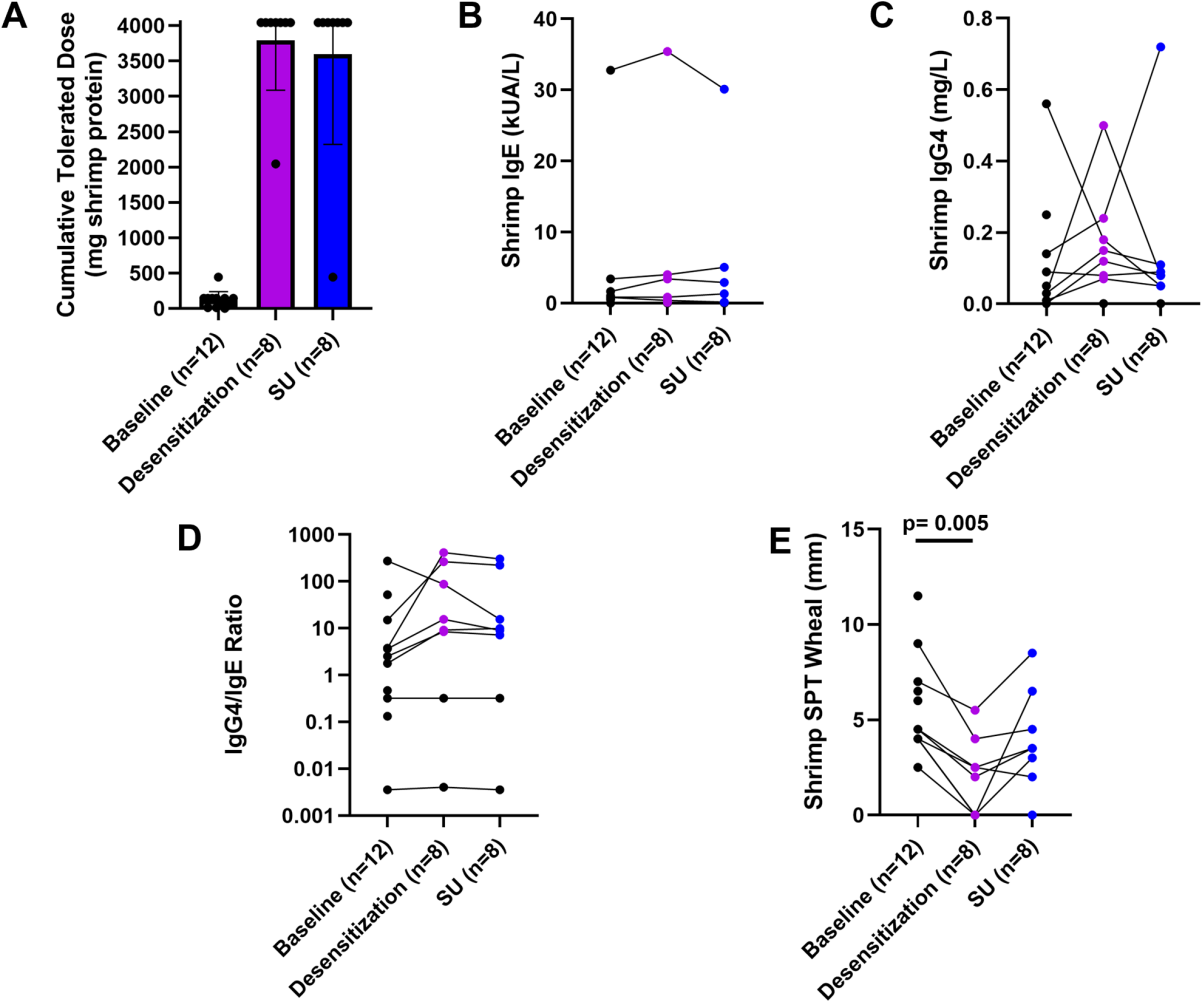
Shrimp OIT outcomes. Shrimp DBPCFC outcomes **(A)**, IgE **(B)**, IgG4 **(C)**, IgG4/IgE ratio, and SPT wheals at baseline (week 0), desensitization (week 52), and sustained unresponsiveness (week 58).

The majority of participants (87.5%, 7/8), who completed OIT were desensitized to 4,043 mg shrimp protein, the maximum assessed cumulative dose in the DBPCFC at week 52. The remaining participant (12.5%, 1/8) was desensitized to 2,043 mg shrimp protein (Figure 3A). Those who tolerated the maximum assessed cumulative dose also (87.5%, 7/8) maintained desensitization to 4,043 mg shrimp protein after 6 weeks of shrimp avoidance at week 58. One participant's CTD (12.5%, 1/8) was reduced to 443 mg shrimp protein after 6 weeks of shrimp avoidance. There were no significant changes in shrimp IgE, shrimp IgG4, or IgG4/IgE ratio (Figures 3B–D). Shrimp OIT decreased the median SPT wheal diameter from 4.25 mm at baseline to 2.5 mm after desensitization (*p* = 0.005, Figure 3E).

**Figure 4:**
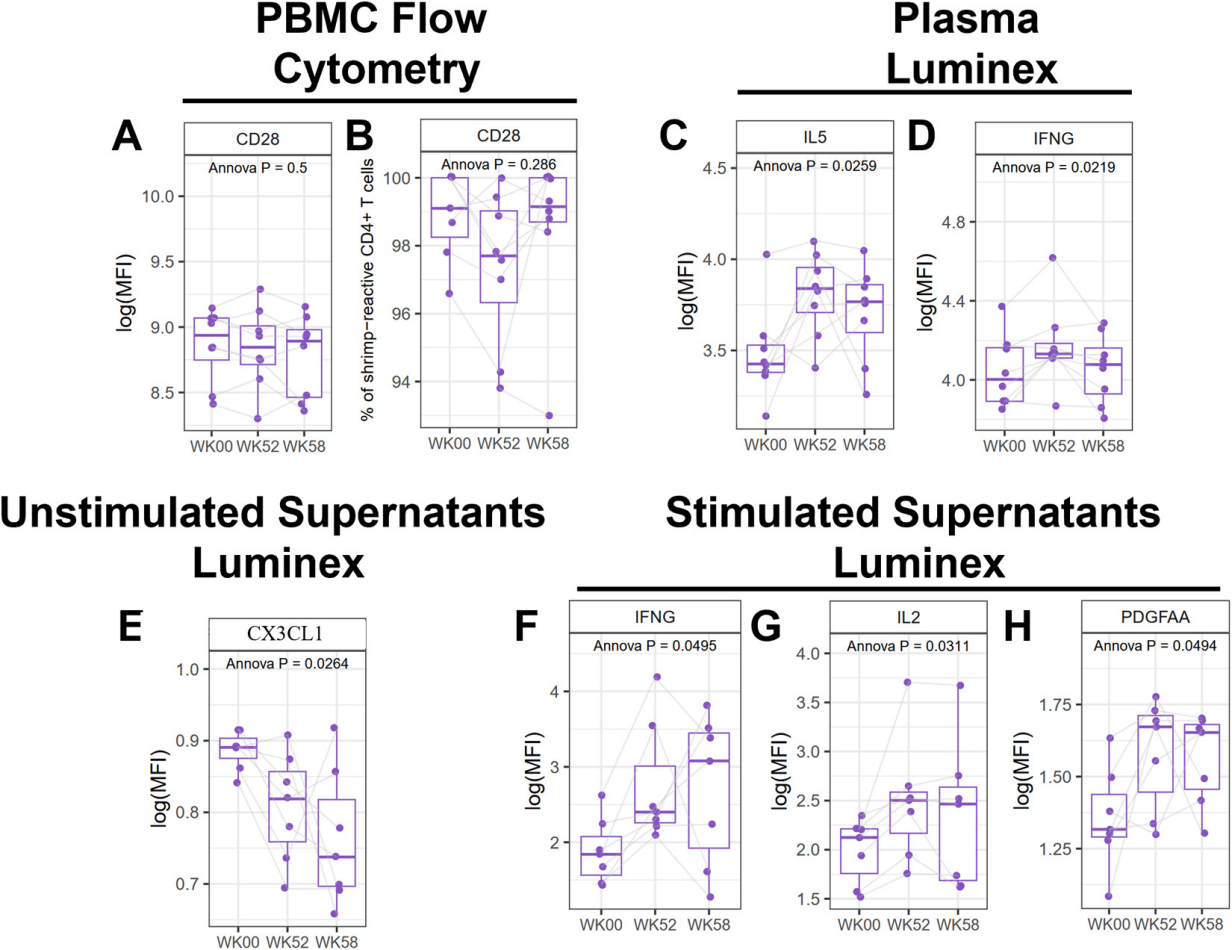
Mechanistic analyses from the MOTIF study. Flow cytometry of PBMCs for CD28 **(A,B)**. Luminex proteomics from plasma **(C,D)**, unstimulated **(E)**, and shrimp-stimulated PBMC culture supernatants **(F–H)**.

The primary outcome for the MOTIF study was the change in expression of CD28 in CD4+ allergen specific (CD154+) T-cells at baseline and 52 weeks. No significant changes in CD28 expression were observed after shrimp OIT (Figures 4A,B, Supplementary Figure S1). Luminex assays from plasma samples identified several genes that were significantly altered in response to shrimp OIT, including upregulation of IL5 (*p* = 0.0259) and interferon gamma (IFNG, *p* = 0.0219; Figures 4C,D, Supplementary Figure S2). Differences in secreted proteins was assessed by Luminex assays from unstimulated or shrimp-stimulated PBMC supernatants. Shrimp OIT was found to decrease the secretion of fractalkine (CX3CL1, *p* = 0.0264; Figure 4E, Supplementary Figure S3) in unstimulated PBMCs and increase the expression of IFNG (*p* = 0.0495), IL2 (*p* = 0.0311), and platelet-derived growth factor AA (PDGFAA, *p* = 0.0494; Figures 4F–H, Supplementary Figure S4).

### Safety

Adverse events (AEs) during shrimp OIT were common (75%, 9/12), however most (88%) were mild (Bock grade 1) and the rest (12%) were moderate (Bock grade 2, Table 2). There were no severe AEs (Bock grade 3), requiring epinephrine, emergency department visits, or cases of anaphylaxis during shrimp OIT in this study. Most AEs (98.8%, 68/69) occurred during the OIT buildup phase. Gastrointestinal (GI) AEs were most common (64%) followed by skin AEs (20%).

**Table 2:** Safety and adverse events for shrimp allergic participants in the MOTIF study.

Stage		Total	OIT Build up w0–w28	OIT Maintenance w29–w52	OIT Avoidance (SU) w53–w58/End of Study w58
Total active participants	12	12	12	8	
Total participants who experienced AE	9 (75%)	9 (75%)	1 (8%)	0	
Total unique AE episodes	69	68	1 (100%)	0	
Total AE experienced	84 (100%)	83 (100%)	1 (100%)	0	
Category	GI	54 (64%)	53 (64%)	1 (100%)	0
	General	3 (4%)	3 (4%)	0	0
	Respiratory	5 (6%)	5 (6%)	0	0
	Skin	17 (20%)	17 (20%)	0	0
	Other	5 (6%)	5 (6%)	0	0
Grade of adverse event	1	61 (88%)	60 (88%)	1 (100%)	0
	2	8 (12%)	8 (12%)	0	0
	3	0	0	0	0
Epinephrine use	0	0	0	0	
Emergency department visit	0	0	0	0	
Concomitant medication	16 (23%)	15 (25%)	1 (100%)	0	
Anaphylaxis	0	0	0	0	

### Shrimp screening assessment

Despite the generally higher reported prevalence of shrimp allergy in comparison to cashew allergy (17, 18), the MOTIF study was much less successful in recruiting shrimp allergic participants. Furthermore, there was a high screen fail rate for shrimp allergic participants (55.6%, 15/27 screened). Considering that we had attempted to enrich for shrimp allergic participants using shrimp IgE and/or SPT, we hypothesized that the thresholds that are generally used for enriching for food allergic participants may be different for shrimp allergy than other allergens. Therefore, we independently evaluated shrimp sensitivity retrospectively in research participants who were screened for shrimp allergy for several different clinical trials (including MOTIF) at the Sean N. Parker Center for Allergy and Asthma Research at Stanford University with a clinical history suggestive for clinical reactivity to shrimp allergy. We identified a screening cohort of 48 participants that had a median of 20.5 years of age (range 7–53 years old), were 41.7% male, and had a diverse racial distribution (Supplementary Table S3). There was a high prevalence of atopic history in this cohort, 83.3% had asthma (*n* = 20), 59.4% had allergic rhinitis (*n* = 19), and 65.2% had atopic dermatitis (*n* = 15).

Only 18 (51.4%) out of the 35 participants with positive sIgE or SPT experienced dose-limiting reactions during the DBPCFC at or below the 300 mg dose (cumulative 443 mg) of shrimp (Supplementary Figure S5). Conversely, 7 (53.8%) out of the 13 participants who had negative sIgE and SPT to shrimp reacted to the 443 mg cumulative shrimp DBPCFC. Many (44%) of the participants were highly sensitized to shrimp and reacted at ≤13 mg shrimp protein (Supplementary Figure S6A). There was no significant difference in the total IgE or sIgE (medians: total IgE: 461.50 vs. 965.80 kUA/L, *p* = 0.45; sIgE: 4.63 vs. 11.80 kUA/L, *p* = 0.20, Supplementary Figures S6B,C), SPT wheal size (4.75 vs. 5 mm, *p* = 0.71, Supplementary Figure S6D), or atopic history (asthma *p* = 0.69, allergic rhinitis *p* = 0.91, atopic dermatitis *p* = 0.40; Supplementary Figure S7) between participants with and without reactivity to DBPCFC.

Some house dust mite (HDM) epitopes including D. *pteronyssinus* and D. *farinae*, are also structurally similar to the common shrimp allergen tropomyosin, leading to allergic cross-reactivity to shrimp (30, 31). These HDM epitopes could interfere with shrimp sIgE and SPT diagnostic assays for shrimp (32) thus increasing the risk of false-positive shrimp diagnoses (33, 34). To assess the potential of cross-reactivity, co-sensitization to HDM (D. *pteronyssinus* and D. *farina)* by SPT was assessed for a subset of 13 participants who had additional assays performed. There was no significant difference in the SPT wheals for either D. *pteronyssinus* (4.25 mm vs. 6.5 mm, *p* = 0.23) or D. *farinae* (5 mm vs. 5.5 mm, *p* = 0.425) between participants with and without reactivity to shrimp DBPCFC (Supplementary Figures S6 E,F).

## Discussion

Despite the relatively high prevalence of shrimp allergy, few studies have assessed the safety and efficacy of shrimp OIT. We found that shrimp OIT is effective at inducing desensitization, with 58.3% of the ITT and 87.5% of the PP group achieving desensitization to 4,043 mg shrimp protein after 52 weeks of shrimp OIT. Furthermore, all of these participants (58.3% of the ITT and 87.5% of the PP group) were able to maintain this level of desensitization after 6 weeks of shrimp avoidance. Although AEs were common during shrimp OIT, with 75% of participants experiencing at least 1 AE. Most of these AEs (88%) were mild (Bock grade 1), with 12% being moderate (Bock grade 2) and no severe reactions. Overall, these findings suggest that shrimp OIT is safe and effective for the treatment of shrimp allergy.

The primary endpoint of the MOTIF study was to investigate how shrimp OIT changed the expression of CD28 in CD4+ allergen specific (CD154+) T-cells at baseline and 52 weeks, however no differences in CD28 expression or other immune populations were observed by flow cytometry. However, Luminex assays did identify several proteins whose expression changed during shrimp OIT. We observed that shrimp OIT increases the expression of IL5 and IFNG on PBMCs in plasma. Shrimp OIT also reduced the release of CX3CL1 in unstimulated PBMCs and increased the release of IFNG, IL2, and PDGFAA in shrimp-stimulated PBMCs. Originally, we intended to look for immune markers that could differentiate between sustained and transient desensitization during OIT, however due to the very high levels of sustained unresponsiveness (87.5% of the PP group), the post-OIT outcomes were not diverse enough to perform this analysis. Further studies are needed to understand differences in the immune responses between different shrimp OIT outcomes.

We encountered unexpected challenges in enrolling participants with shrimp allergy in the MOTIF study with a screen fail rate of 55.6%, despite the attempted enrichment of shrimp sensitized participants using SPT and sIgE tests which has previously been successful for screening for other allergens (8, 26, 27, 35). This prompted us to evaluate the utility of the screening tools for shrimp OIT clinical trials. For clinical trials, cost-effective and accessible screening tools that can accurately screen for individuals who are likely to have reactive DBPCFC outcomes are desired, however our analysis suggests that assays such as SPT and sIgE are not correlated with DBPCFC outcomes. Only 51.4% of participants with positive SPT and/or sIgE to shrimp had a dose-limiting reaction at or below 300 mg of shrimp during DBPCFCs, therefore SPT and sIgE are currently insufficient as a pre-screening tool for shrimp allergy clinical trials. Furthermore, 53.8% (7/13) of participants with negative sIgE and SPT, but positive clinical history, had reactivity to DBPCFC. It is possible that these participants are allergic to shrimp epitopes/components that are not present in the SPT extracts or sIgE assays used in this study. In line with this, there are other major shrimp allergens such as hemocyanin, which can be even more abundant in shrimp than the commonly assessed tropomyosin depending on the source of the shrimp (36). Unfortunately, we did not find any association between any of the components we tested here with DBPCFC outcomes, suggesting that they are currently not ready to be used for shrimp clinical trial screening. Other studies suggest that shrimp component resolved diagnostics and basophil activation tests may have better diagnostic value than traditional SPT and IgE tests (37–41). Screening DBPCFCs were conducted up to a 300 mg dose (443 mg cumulative dose), aligning with the established standard for food allergy standards (42). Our pre-screening cohort spanned a diverse age range from children to adults and demonstrated a balanced gender distribution (41.7% male). While our cohort included significant proportions of Whites (51.1%) and Asians (26.7%), there was an underrepresentation of other racial groups.

Our assessment of the safety and efficacy of shrimp OIT in the MOTIF study had several limitations including its small cohort size (12 shrimp allergic participants), single-site design, and limited mechanistic data. Larger multi-site studies are needed to further define the efficacy and safety of shrimp OIT. In light of our retrospective screening assessment and DBPCFCs performed under Prescreening, further studies are needed to define better approaches to enriching for shrimp allergic participants for clinical trials. While our findings show that SPT and IgE tests are not associated with DBPCFC outcomes to 443 mg cumulative shrimp, outside of clinical trials shrimp is often diagnosed at much higher thresholds on the order of grams (38, 39). This suggests that shrimp should not be treated like other allergens such as peanut for clinical trials and a higher threshold should be used for clinical trial inclusion. Therefore, further studies are needed to better characterize the differences between shrimp and more commonly studied allergens.

## Data Availability

The raw data supporting the conclusions of this article will be made available by the authors, without undue reservation.

## Supplementary material

Supplementary Figure S1 Immune marker expression in PBMCs during shrimp OIT.

Supplementary Figure S2 Luminex assay from plasma during shrimp OIT.

Supplementary Figure S3 Luminex assay from unstimulated PBMC culture supernatants.

Supplementary Figure S4 Luminex assay from shrimp-stimulated PBMC culture supernatants.

Supplementary Figure S5 Consort diagram for retrospective chart review.

Supplementary Figure S6 Allergen sensitization in participants with positive or negative shrimp DBPCFC outcomes from retrospective chart review.

Supplementary Figure S7 Associations of baseline characteristics and the DBPCFC outcome.

Supplementary Table S1 Dosing steps for shrimp OIT.

Supplementary Table S2 Staining panel for FACS analysis.

Supplementary Table S3 Screening demographics for participants screened for shrimp allergy for multiple studies.
