# Supplementary material for: Shrimp oral immunotherapy outcomes in the phase 2 clinical trial: MOTIF
Source: Front Allergy. 2025 Jul 22;6:1458131. doi: 10.3389/falgy.2025.1458131 (PMC12321884; doi:10.3389/falgy.2025.1458131)
Supplement: Supplementary Figure S1 — Immune marker expression in PBMCs during shrimp OIT. [file Datasheet1.pdf]

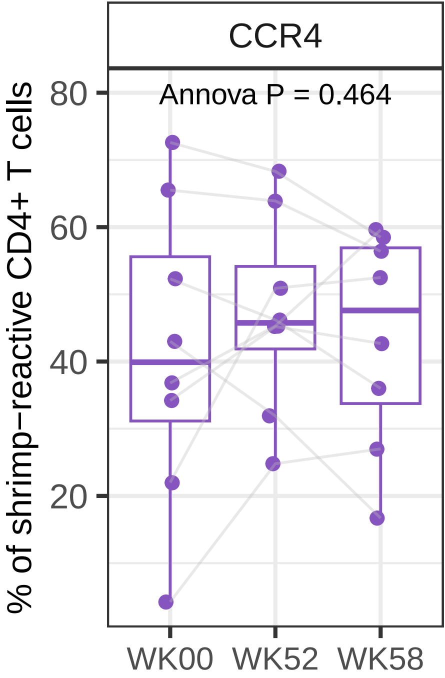
CCR4
Annova P = 0.464
% of shrimp−reactive CD4+ T cells
80
60
40
20
WK00
WK52
WK58

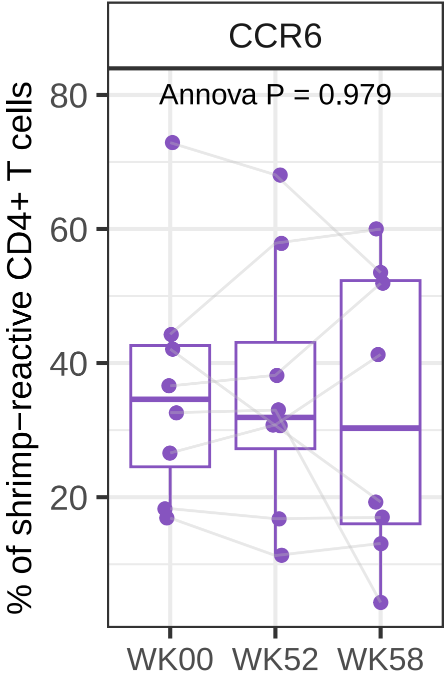
CCR6
Annova P = 0.979
% of shrimp−reactive CD4+ T cells
80
60
40
20
WK00
WK52
WK58

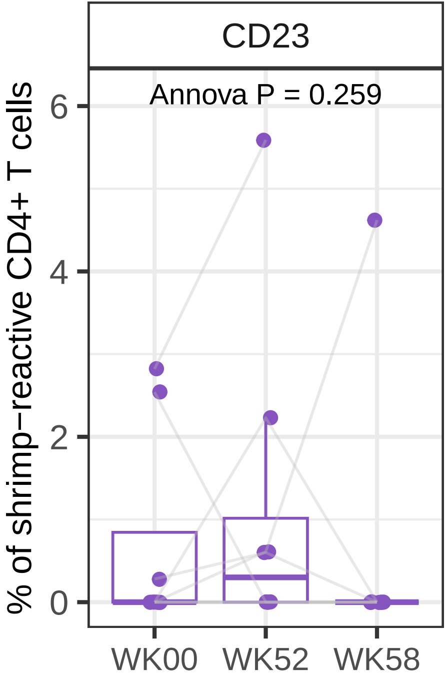
CD23
Annova P = 0.259
% of shrimp−reactive CD4+ T cells
0
2
4
6
WK00
WK52
WK58

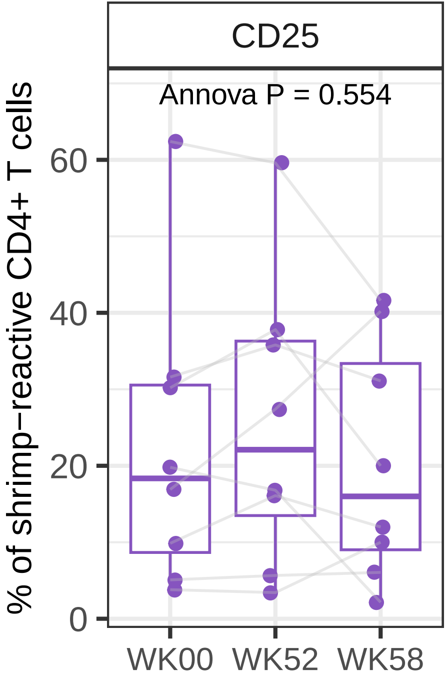
CD25
Annova P = 0.554
% of shrimp−reactive CD4+ T cells
60
40
20
0
WK00
WK52
WK58

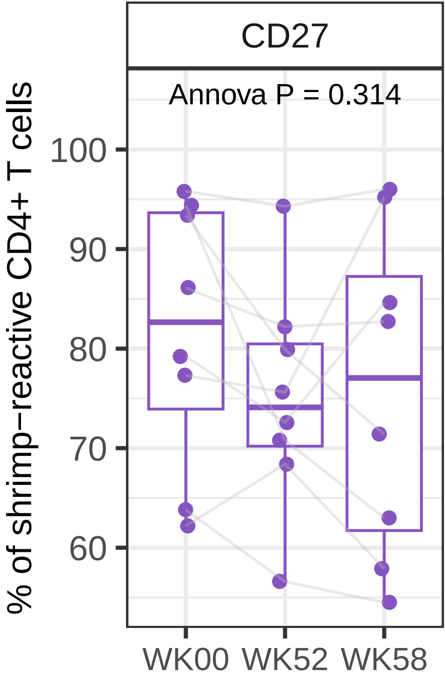
CD27
Annova P = 0.314
% of shrimp−reactive CD4+ T cells
100
90
80
70
60
WK00
WK52
WK58

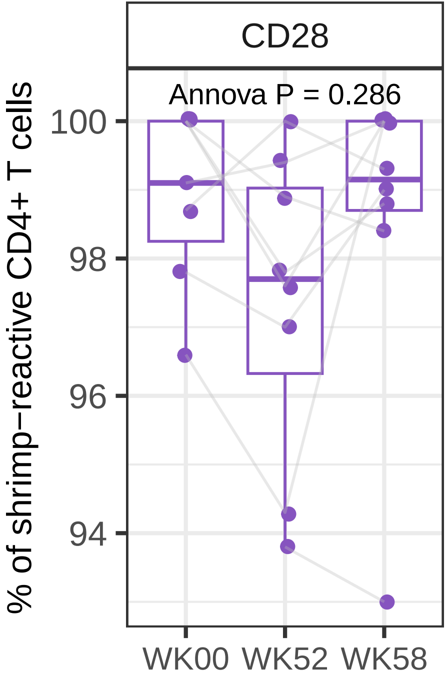
CD28
Annova P = 0.286
% of shrimp−reactive CD4+ T cells
100
98
96
94
WK00
WK52
WK58

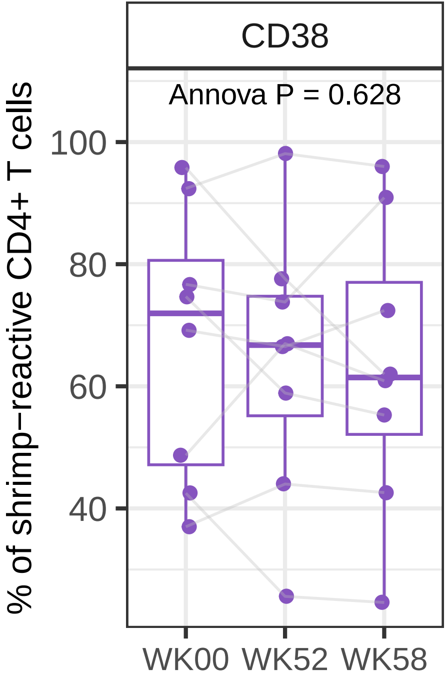
CD38
Annova P = 0.628
% of shrimp−reactive CD4+ T cells
100
80
60
40
WK00
WK52
WK58

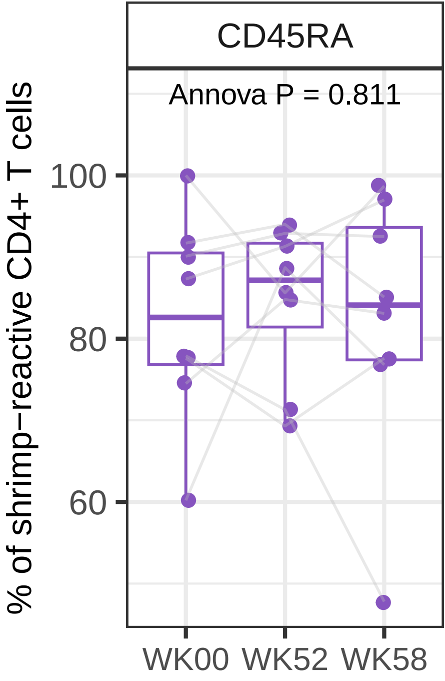
CD45RA
Annova P = 0.811
% of shrimp−reactive CD4+ T cells
100
80
60
WK00
WK52
WK58

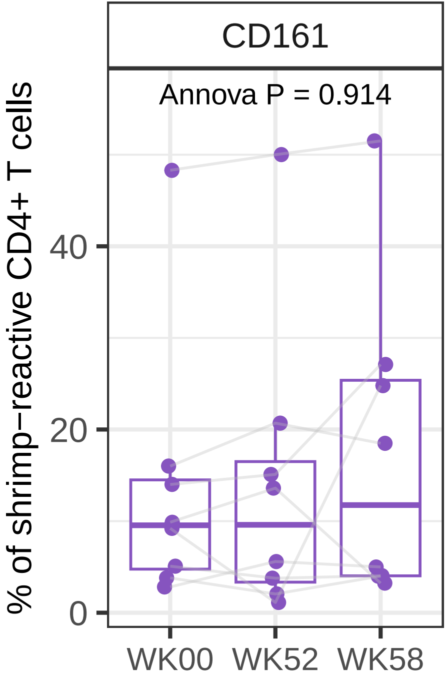
CD161
Annova P = 0.914
% of shrimp−reactive CD4+ T cells
0
20
40
WK00
WK52
WK58

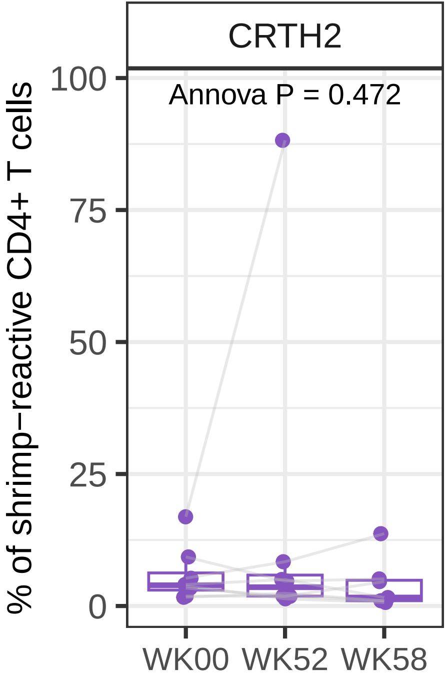

CRTH2
Annova P = 0.472
% of shrimp−reactive CD4+ T cells
100
75
50
25
0
WK00
WK52
WK58

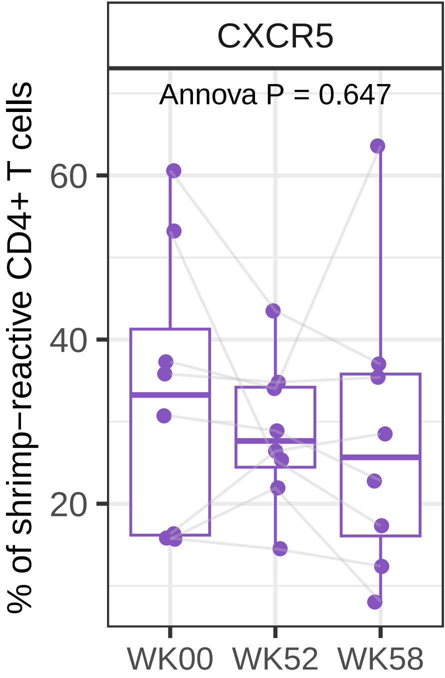

CXCR5
Annova P = 0.647
% of shrimp−reactive CD4+ T cells
60
40
20
WK00
WK52
WK58

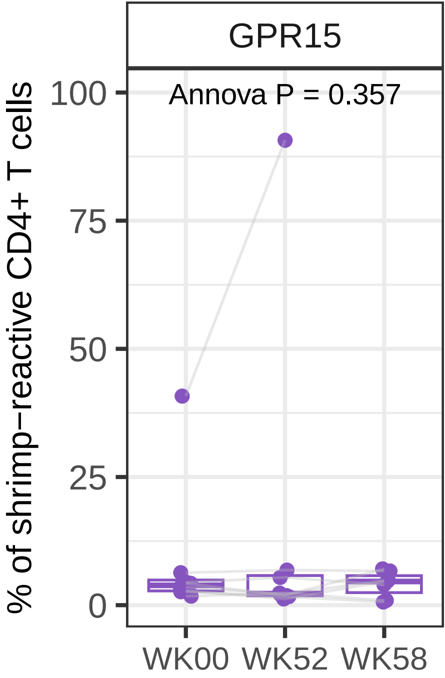
GPR15
Annova P = 0.357
% of shrimp−reactive CD4+ T cells
100
75
50
25
0
WK00
WK52
WK58

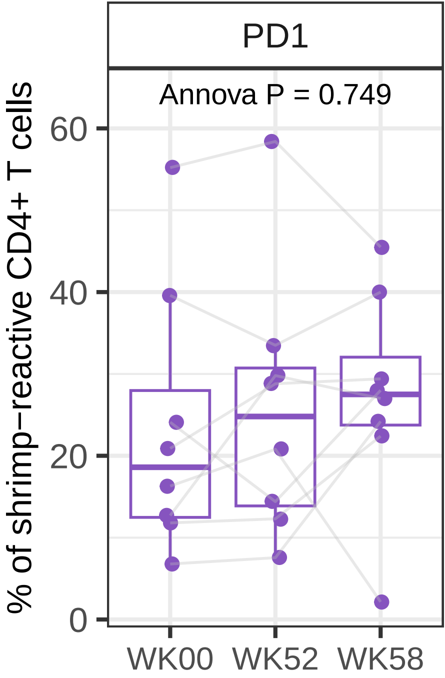
PD1
Annova P = 0.749
% of shrimp−reactive CD4+ T cells
60
40
20
0
WK00
WK52
WK58

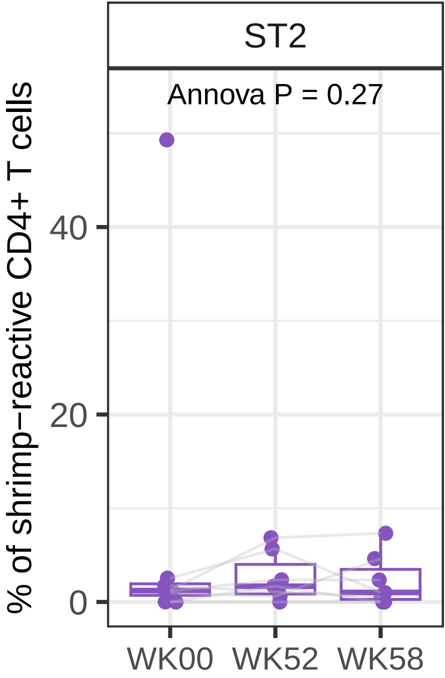

ST2
Annova P = 0.27
% of shrimp−reactive CD4+ T cells
40
20
0
WK00
WK52
WK58

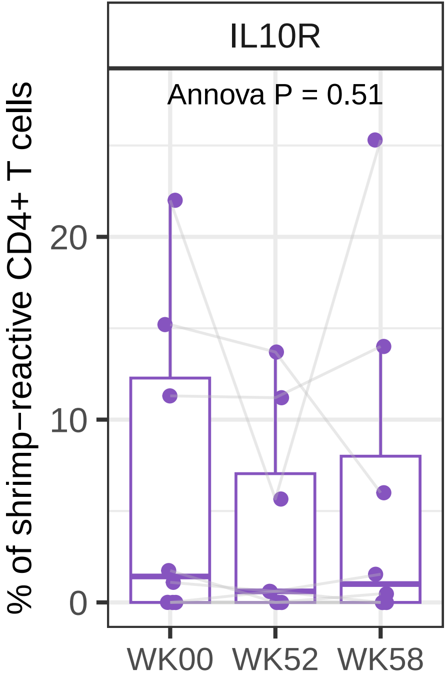

IL10R
Annova P = 0.51
% of shrimp−reactive CD4+ T cells
20
10
0
WK00
WK52
WK58

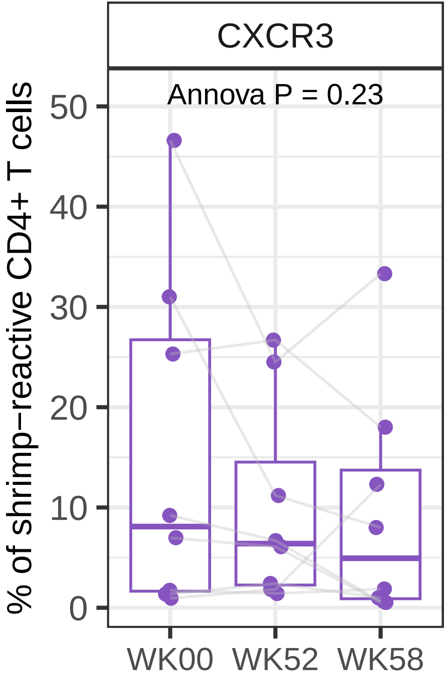
CXCR3
Annova P = 0.23
% of shrimp−reactive CD4+ T cells
0
10
20
30
40
50
WK00
WK52
WK58
