# Supplementary material for: Shrimp oral immunotherapy outcomes in the phase 2 clinical trial: MOTIF
Source: Front Allergy. 2025 Jul 22;6:1458131. doi: 10.3389/falgy.2025.1458131 (PMC12321884; doi:10.3389/falgy.2025.1458131)
Supplement: Supplementary Figure S2 — Luminex assay from plasma during shrimp OIT. [file Datasheet2.pdf]

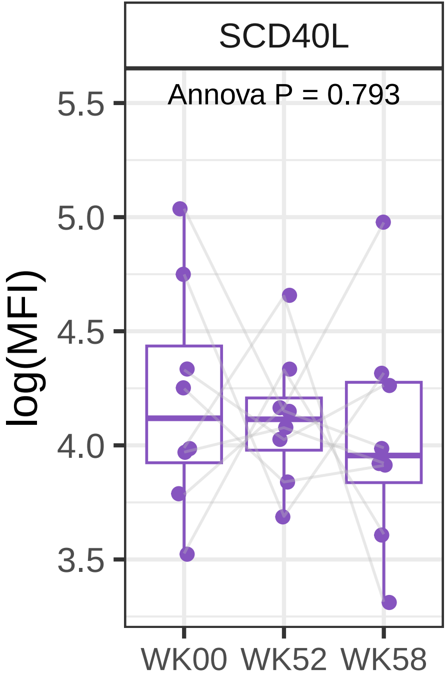
SCD40L
Annova P = 0.793
log(MFI)
5.5
5.0
4.5
4.0
3.5
WK00
WK52
WK58

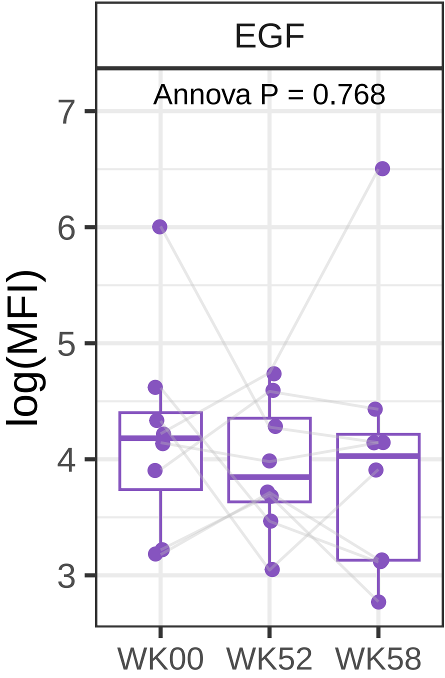
EGF
Annova P = 0.768
log(MFI)
7
6
5
4
3
WK00
WK52
WK58

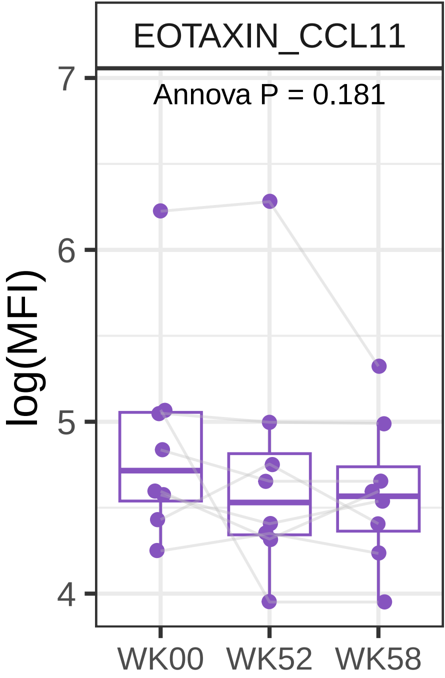
EOTAXIN_CCL11
Annova P = 0.181
log(MFI)
7
6
5
4
WK00
WK52
WK58

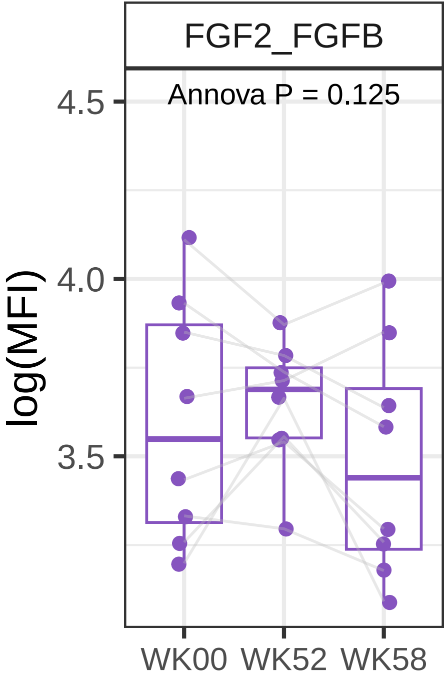
FGF2_FGFB
Annova P = 0.125
log(MFI)
4.5
4.0
3.5
WK00
WK52
WK58

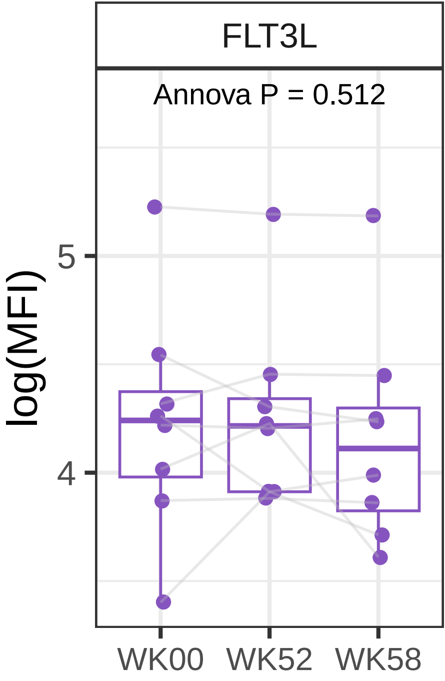
FLT3L
Annova P = 0.512
log(MFI)
5
4
WK00
WK52
WK58

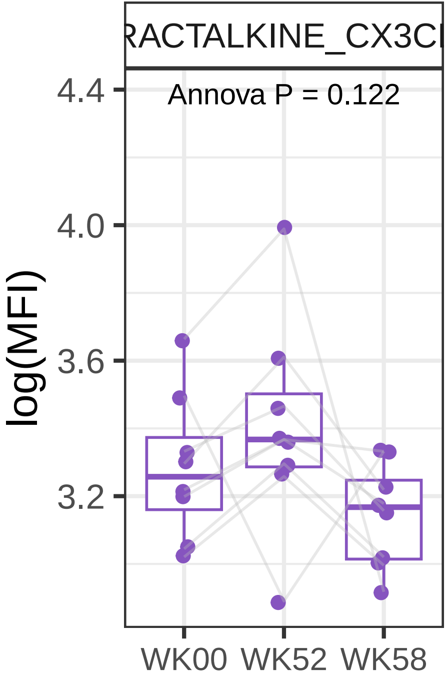
RACTALKINE_CX3CL
Annova P = 0.122
4.4
4.0
3.6
3.2
log(MFI)
WK00
WK52
WK58

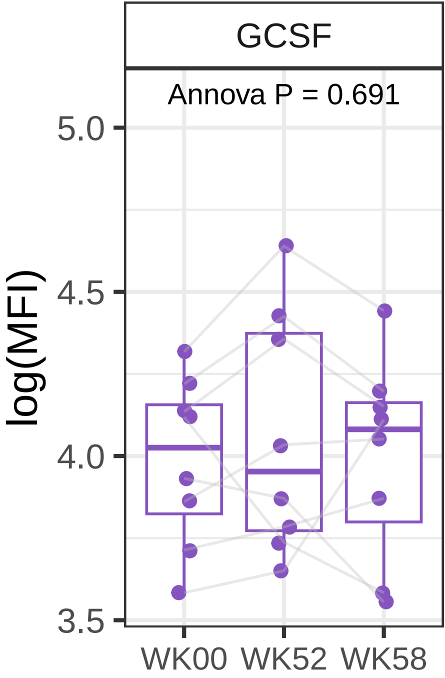
GCSF
Annova P = 0.691
log(MFI)
5.0
4.5
4.0
3.5
WK00
WK52
WK58

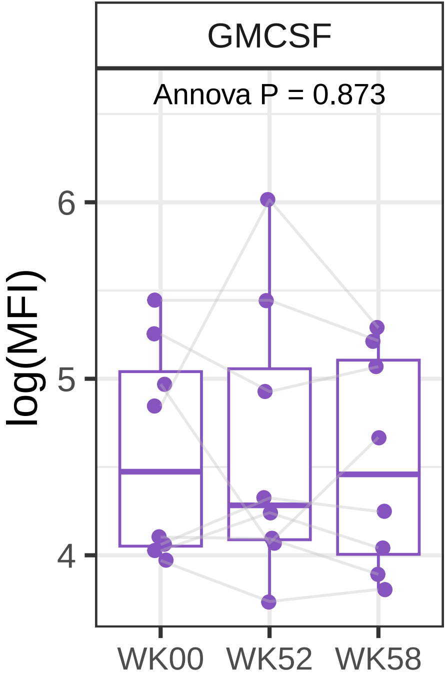
GMCSF
Annova P = 0.873
log(MFI)
6
5
4
WK00
WK52
WK58

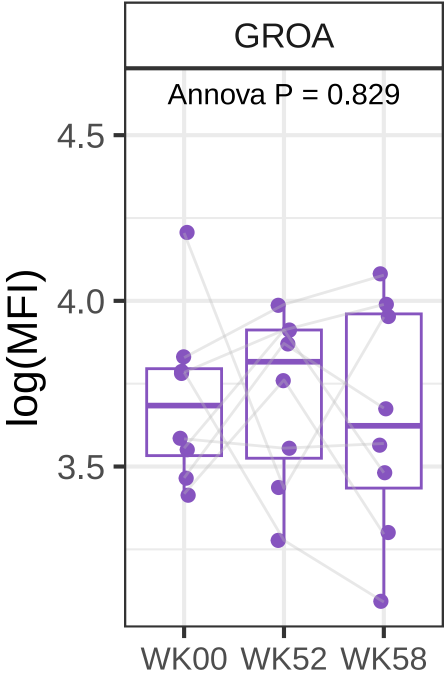
GROA
Annova P = 0.829
log(MFI)
4.5
4.0
3.5
WK00
WK52
WK58

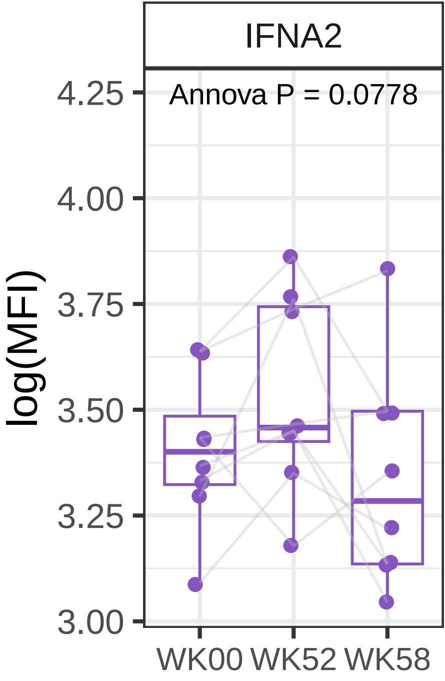

IFNA2
Annova P = 0.0778
log(MFI)
4.25
4.00
3.75
3.50
3.25
3.00
WK00
WK52
WK58

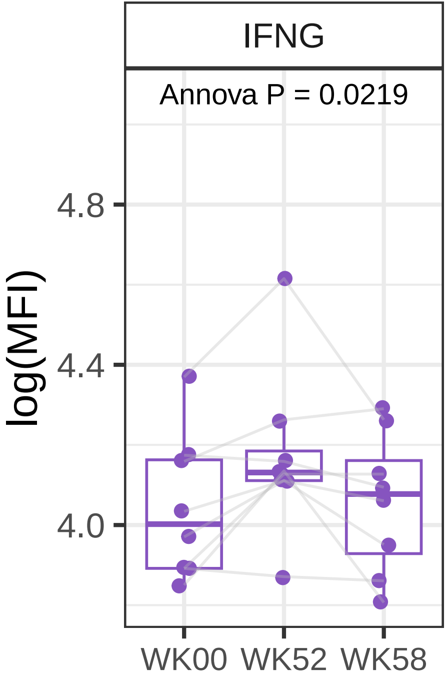
IFNG
Annova P = 0.0219
log(MFI)
4.8
4.4
4.0
WK00
WK52
WK58

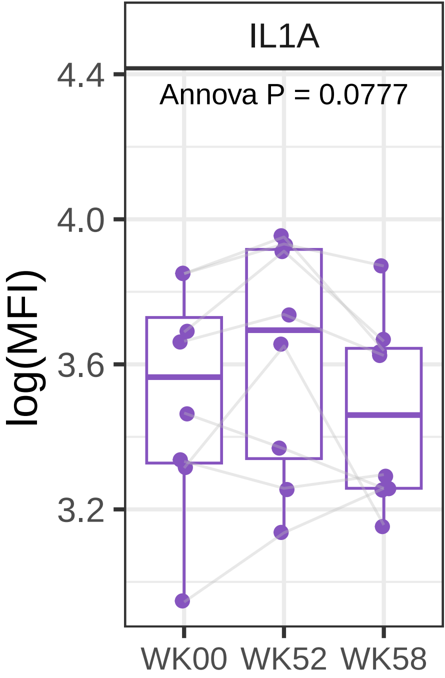
IL1A
Annova P = 0.0777
log(MFI)
4.4
4.0
3.6
3.2
WK00
WK52
WK58

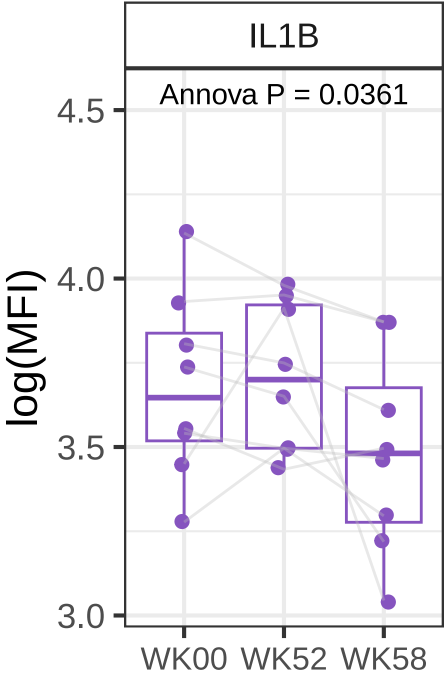
IL1B
Annova P = 0.0361
log(MFI)
4.5
4.0
3.5
3.0
WK00
WK52
WK58

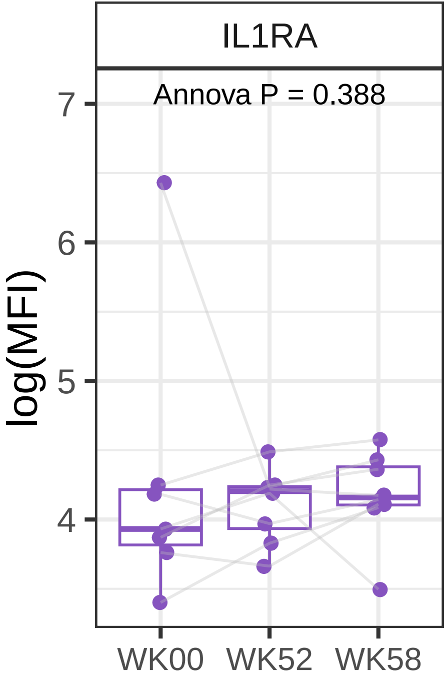
IL1RA
Annova P = 0.388
log(MFI)
7
6
5
4
WK00
WK52
WK58

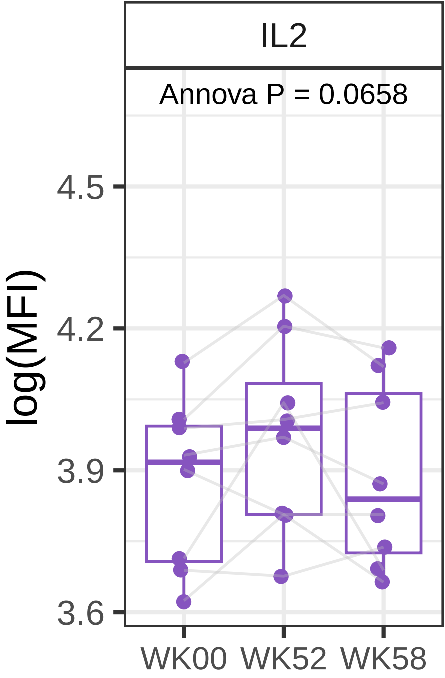
IL2
Annova P = 0.0658
log(MFI)
4.5
4.2
3.9
3.6
WK00
WK52
WK58

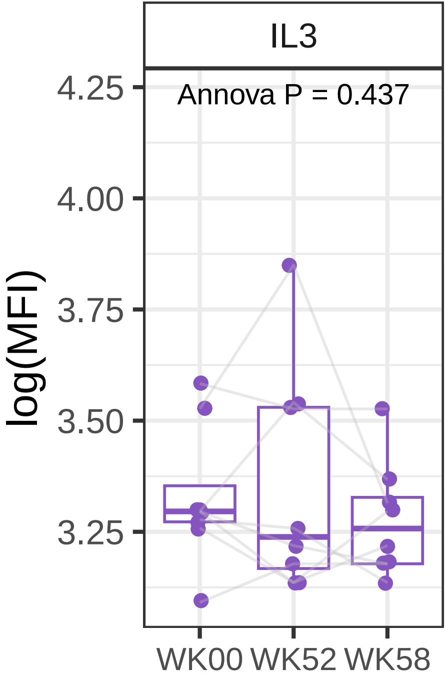
IL3
Annova P = 0.437
4.25
4.00
3.75
3.50
3.25
log(MFI)
WK00
WK52
WK58

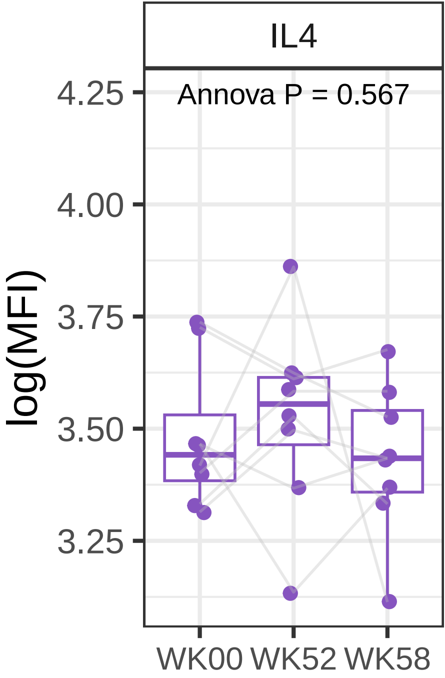
IL4
Annova P = 0.567
log(MFI)
4.25
4.00
3.75
3.50
3.25
WK00
WK52
WK58

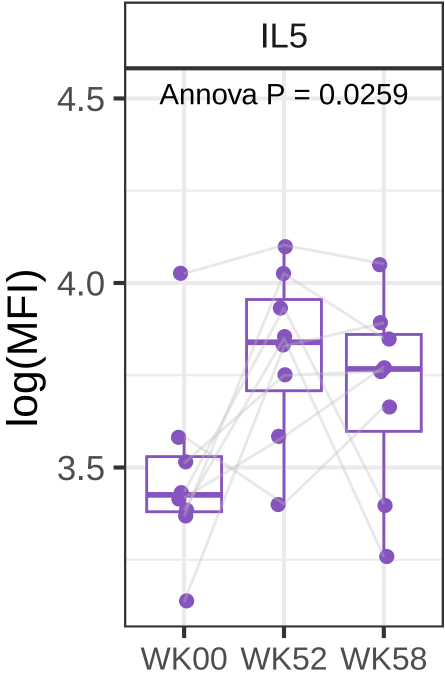

IL5
Annova P = 0.0259
log(MFI)
4.5
4.0
3.5
WK00
WK52
WK58

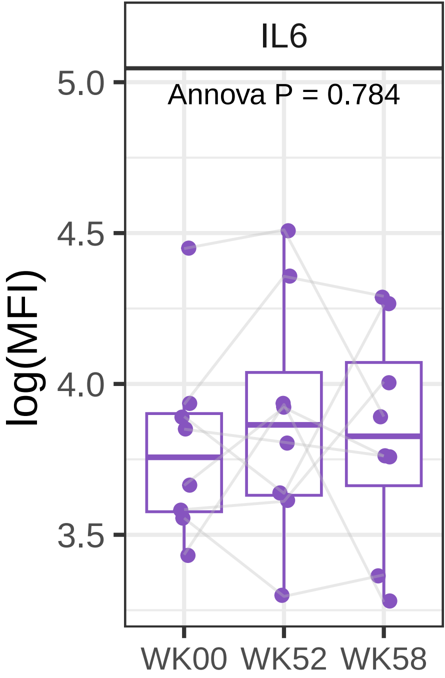
IL6
Annova P = 0.784
log(MFI)
5.0
4.5
4.0
3.5
WK00
WK52
WK58

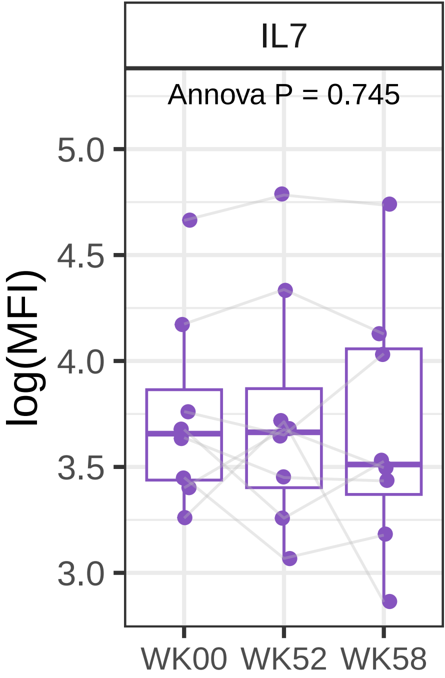
IL7
Annova P = 0.745
log(MFI)
5.0
4.5
4.0
3.5
3.0
WK00
WK52
WK58

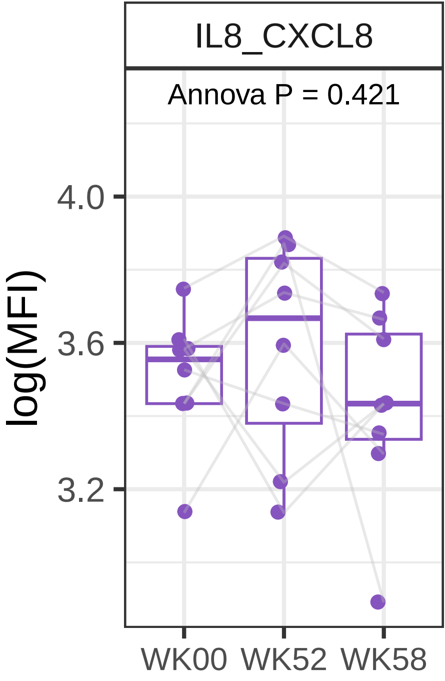
IL8_CXCL8
Annova P = 0.421
log(MFI)
4.0
3.6
3.2
WK00
WK52
WK58

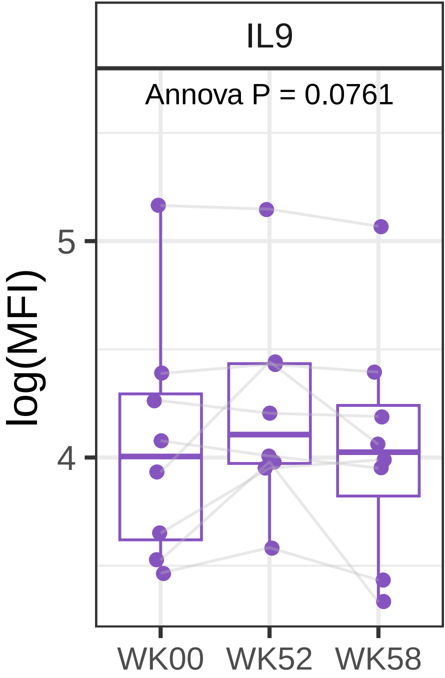
IL9
Annova P = 0.0761
log(MFI)
5
4
WK00
WK52
WK58

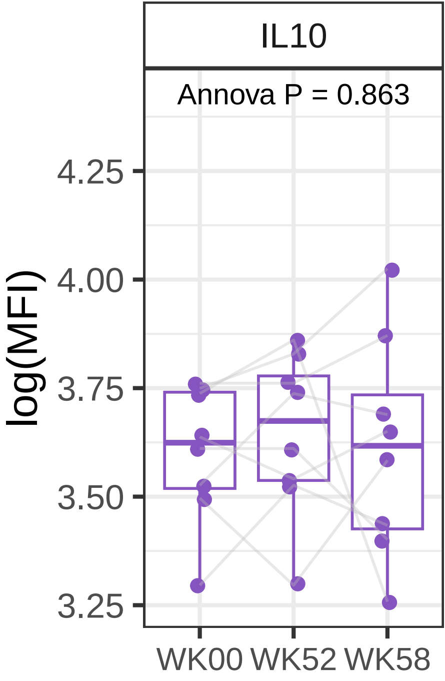
IL10
Annova P = 0.863
log(MFI)
4.25
4.00
3.75
3.50
3.25
WK00
WK52
WK58

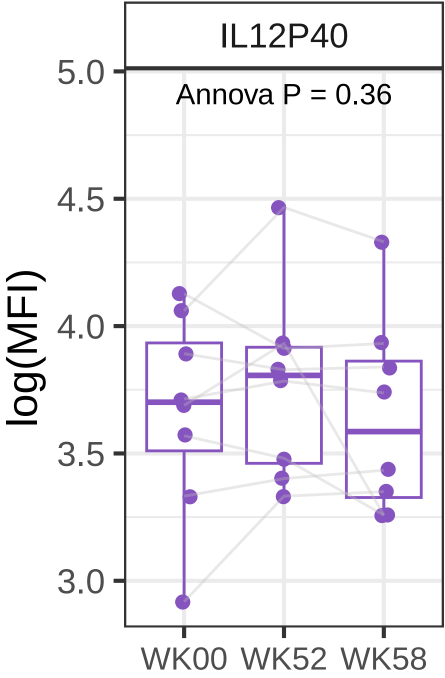
IL12P40
Annova P = 0.36
log(MFI)
5.0
4.5
4.0
3.5
3.0
WK00
WK52
WK58

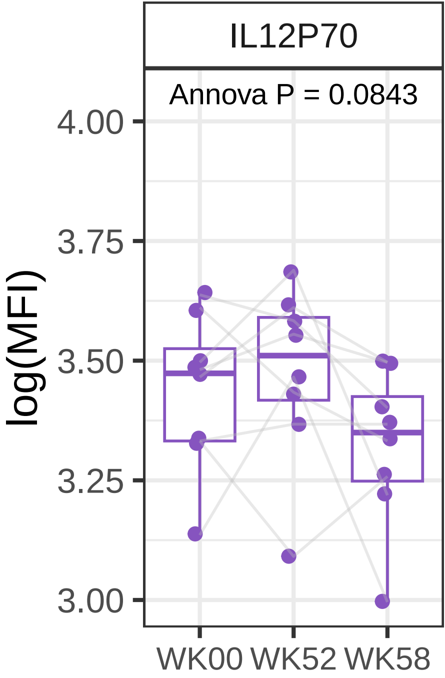
IL12P70
Annova P = 0.0843
log(MFI)
4.00
3.75
3.50
3.25
3.00
WK00
WK52
WK58

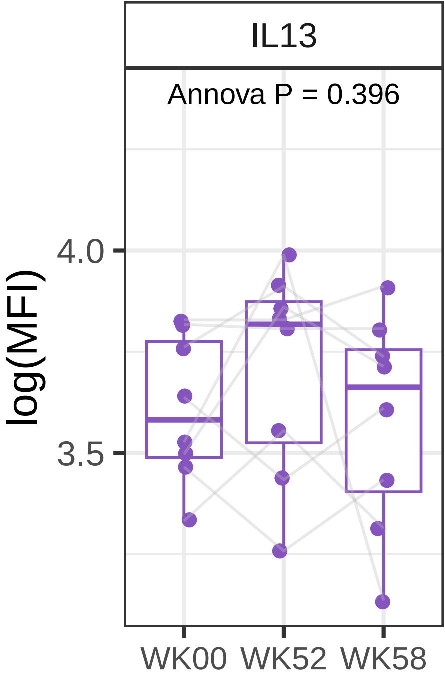
IL13
Annova P = 0.396
log(MFI)
4.0
3.5
WK00
WK52
WK58

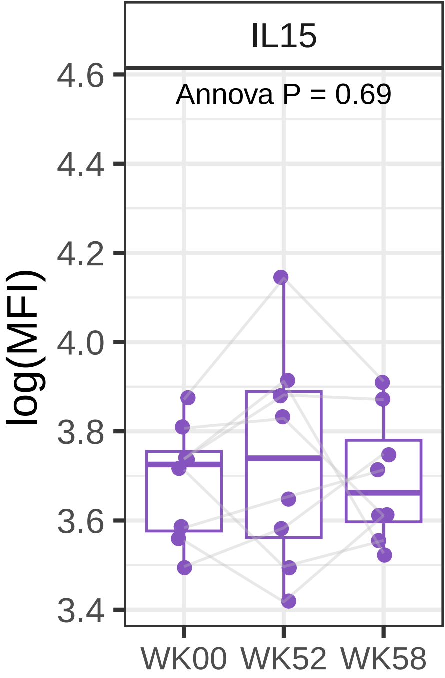
IL15
Annova P = 0.69
log(MFI)
4.6
4.4
4.2
4.0
3.8
3.6
3.4
WK00
WK52
WK58

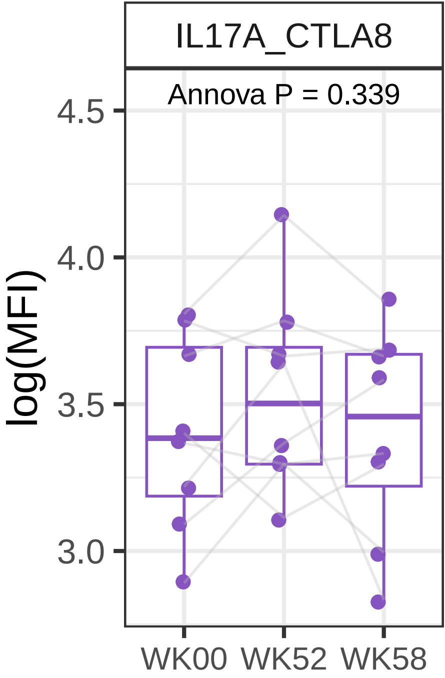

IL17A_CTLA8
Annova P = 0.339
4.5
4.0
3.5
3.0
log(MFI)
WK00
WK52
WK58

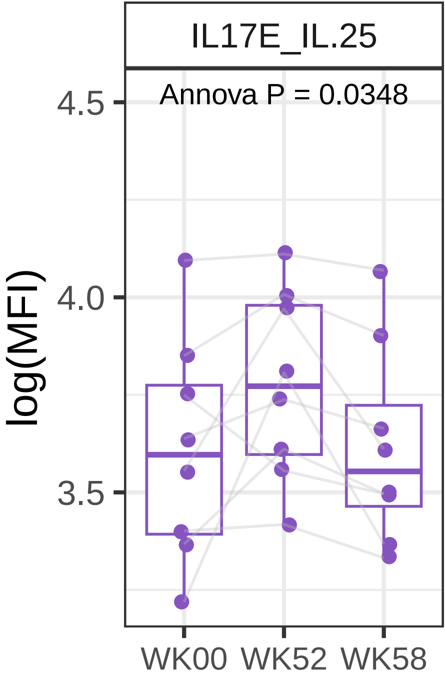
IL17E_IL.25
Annova P = 0.0348
log(MFI)
4.5
4.0
3.5
WK00
WK52
WK58

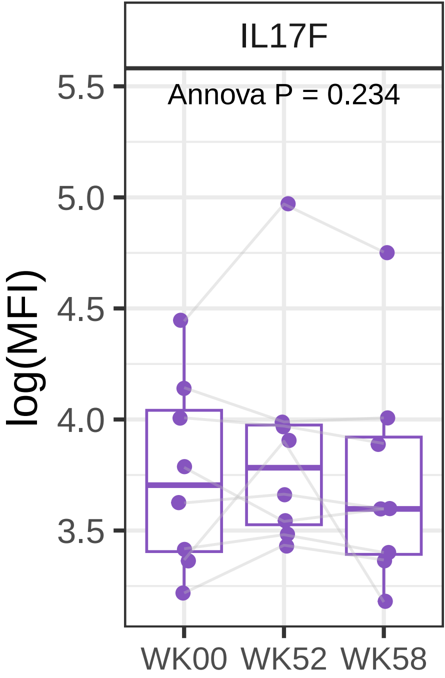
IL17F
Annova P = 0.234
log(MFI)
5.5
5.0
4.5
4.0
3.5
WK00
WK52
WK58

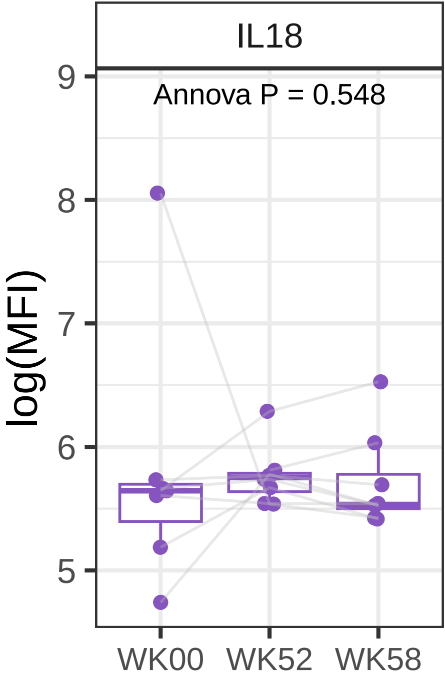
IL18
Annova P = 0.548
log(MFI)
9
8
7
6
5
WK00
WK52
WK58

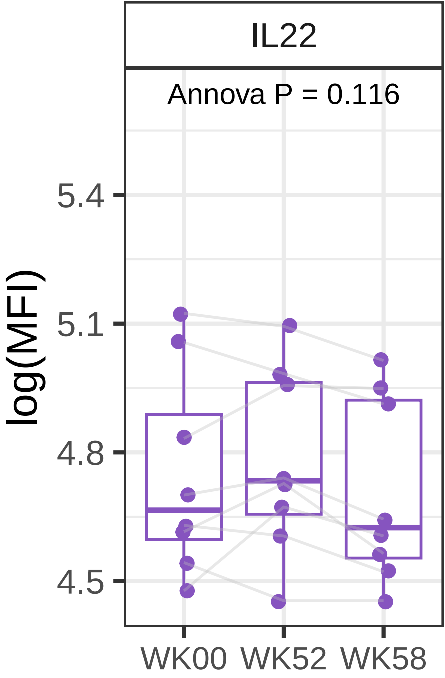
IL22
Annova P = 0.116
log(MFI)
5.4
5.1
4.8
4.5
WK00
WK52
WK58

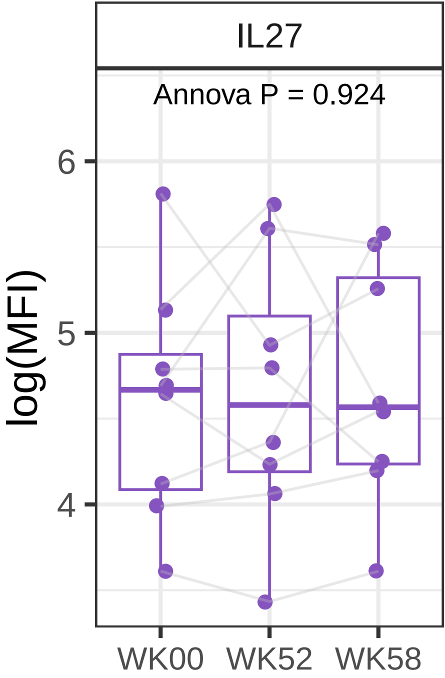
IL27
Annova P = 0.924
log(MFI)
6
5
4
WK00
WK52
WK58

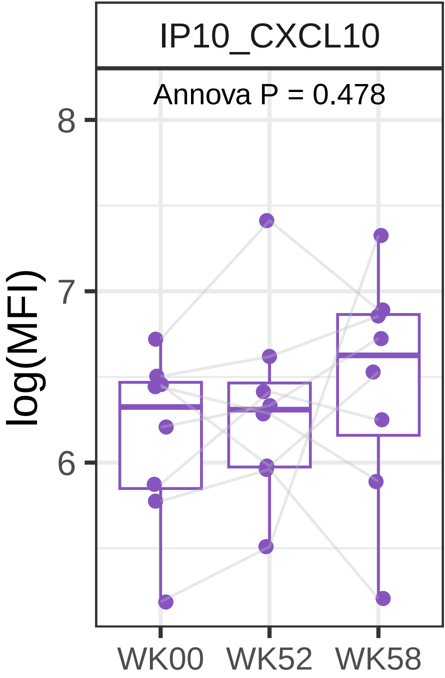
IP10_CXCL10
Annova P = 0.478
8
7
6
log(MFI)
WK00
WK52
WK58

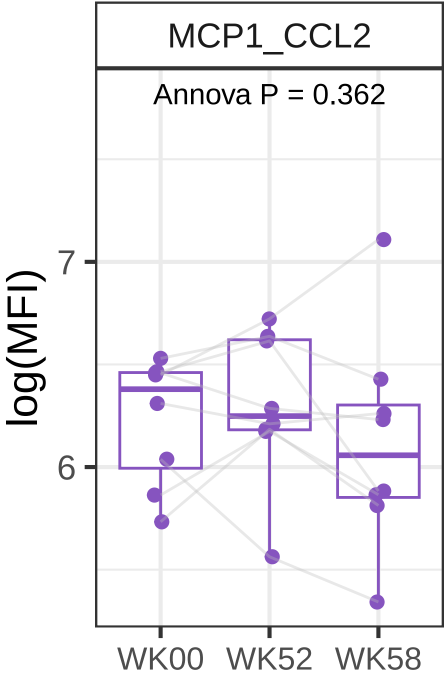

MCP1_CCL2
Annova P = 0.362
log(MFI)
7
6
WK00
WK52
WK58

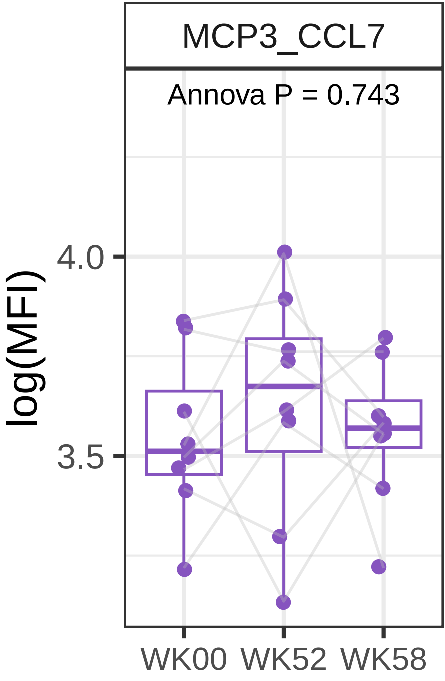

MCP3_CCL7
Annova P = 0.743
log(MFI)
4.0
3.5
WK00
WK52
WK58

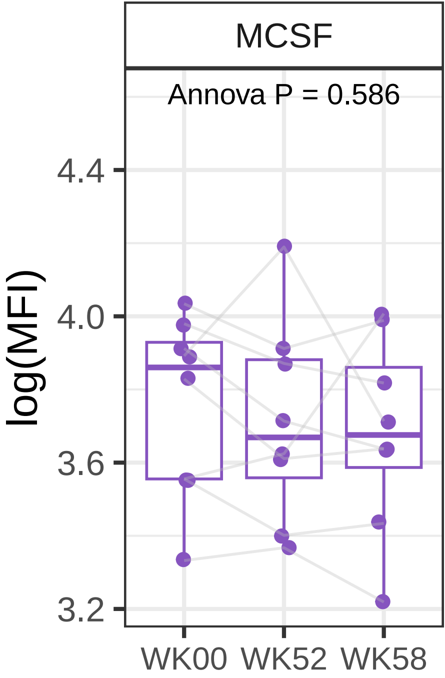

MCSF
Annova P = 0.586
log(MFI)
4.4
4.0
3.6
3.2
WK00
WK52
WK58

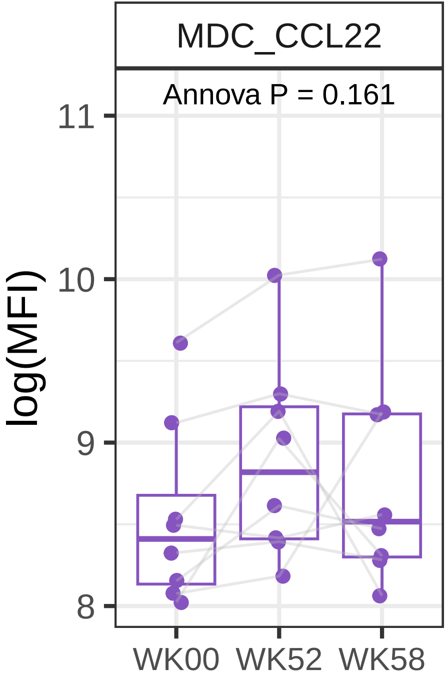

MDC_CCL22
Annova P = 0.161
log(MFI)
11
10
9
8
WK00
WK52
WK58

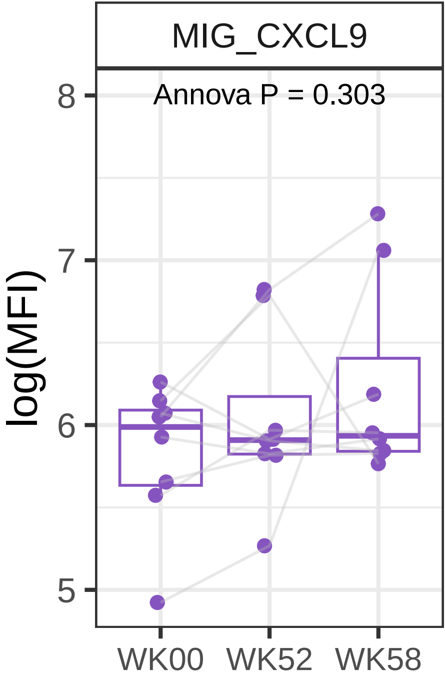
MIG_CXCL9
Annova P = 0.303
log(MFI)
8
7
6
5
WK00
WK52
WK58

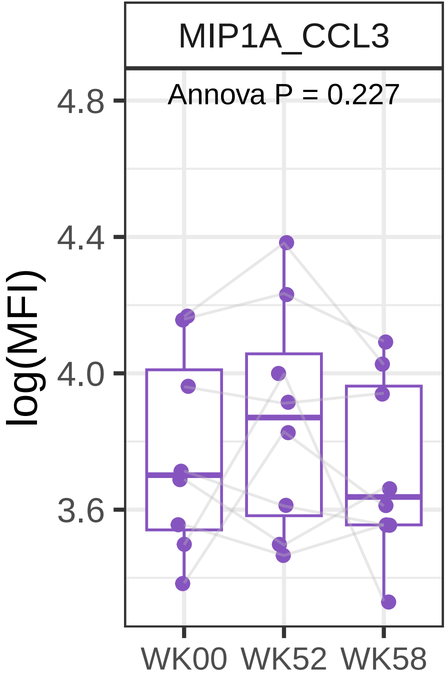

MIP1A_CCL3
Annova P = 0.227
log(MFI)
4.8
4.4
4.0
3.6
WK00
WK52
WK58

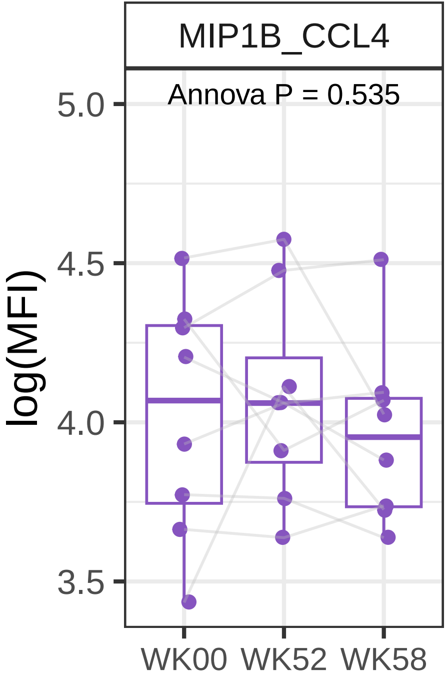
MIP1B_CCL4
Annova P = 0.535
log(MFI)
5.0
4.5
4.0
3.5
WK00
WK52
WK58

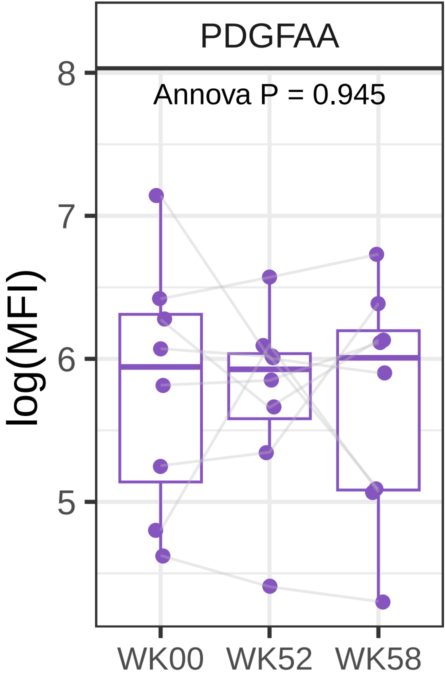
PDGFAA
Annova P = 0.945
log(MFI)
8
7
6
5
WK00
WK52
WK58

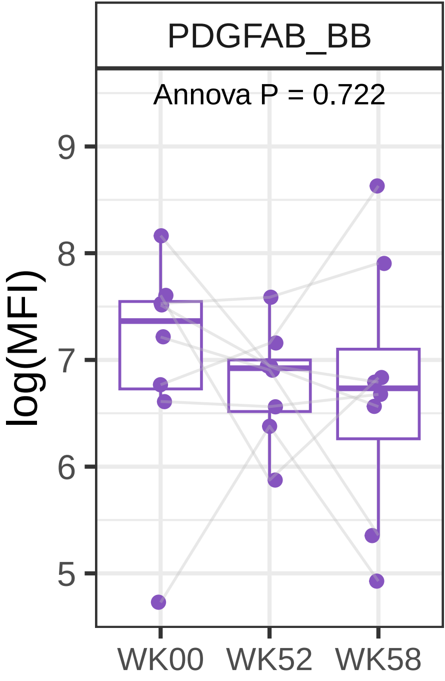
PDGFAB_BB
Annova P = 0.722
log(MFI)
9
8
7
6
5
WK00
WK52
WK58

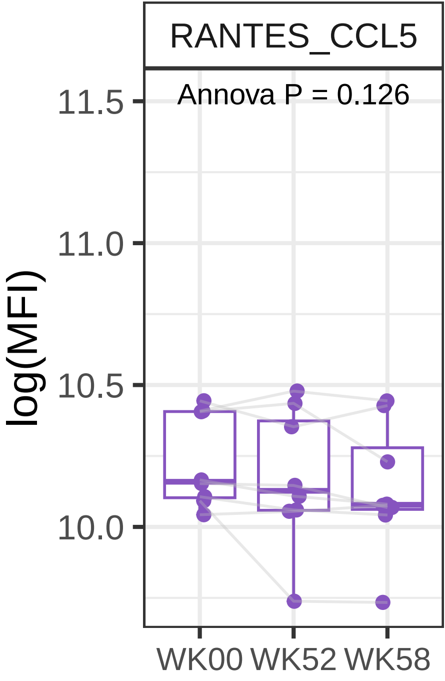
RANTES_CCL5
Annova P = 0.126
log(MFI)
11.5
11.0
10.5
10.0
WK00
WK52
WK58

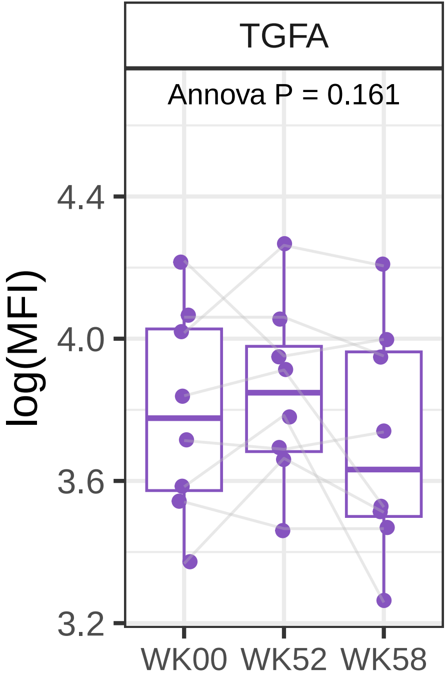
TGFA
Annova P = 0.161
4.4
4.0
3.6
3.2
log(MFI)
WK00
WK52
WK58

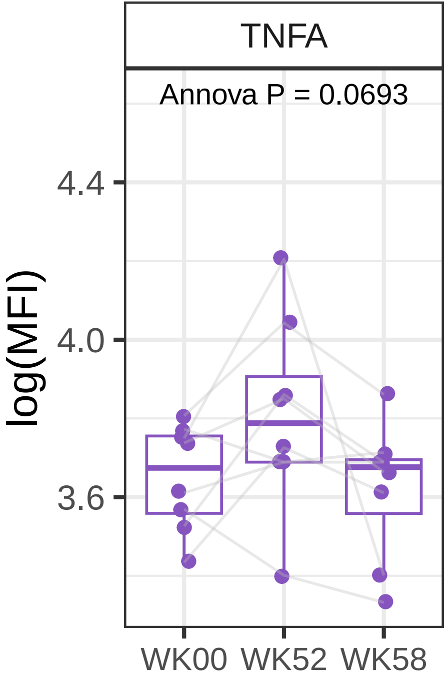

TNFA
Annova P = 0.0693
log(MFI)
4.4
4.0
3.6
WK00
WK52
WK58

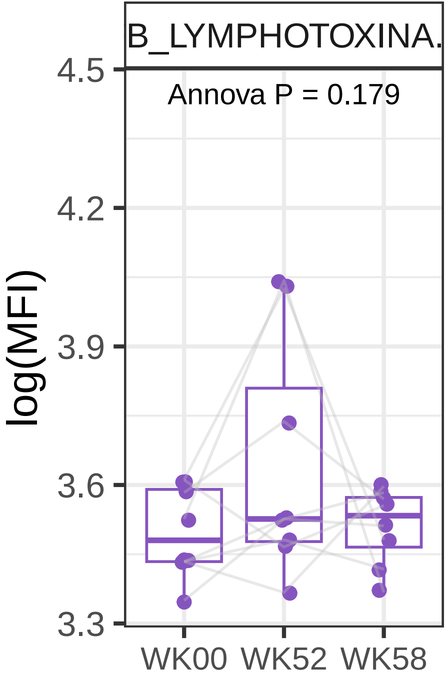

B_LYMPHOTOXINA.
Annova P = 0.179
log(MFI)
4.5
4.2
3.9
3.6
3.3
WK00
WK52
WK58

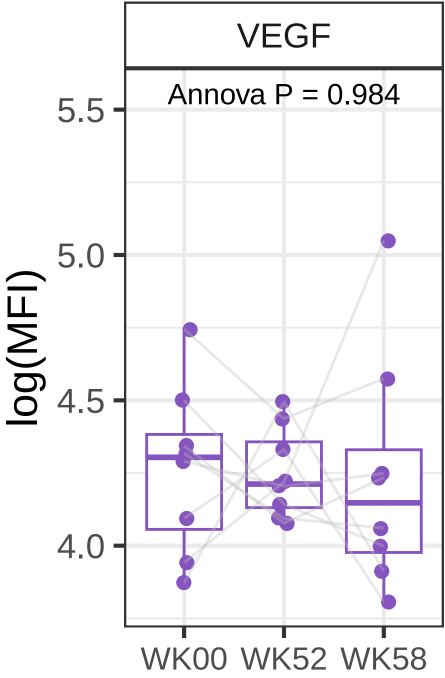

VEGF
Annova P = 0.984
log(MFI)
5.5
5.0
4.5
4.0
WK00
WK52
WK58

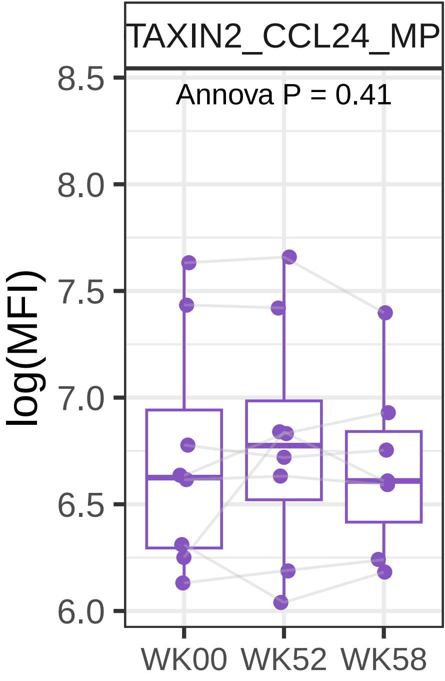

TAXIN2_CCL24_MP
Annova P = 0.41
log(MFI)
8.5
8.0
7.5
7.0
6.5
6.0
WK00
WK52
WK58

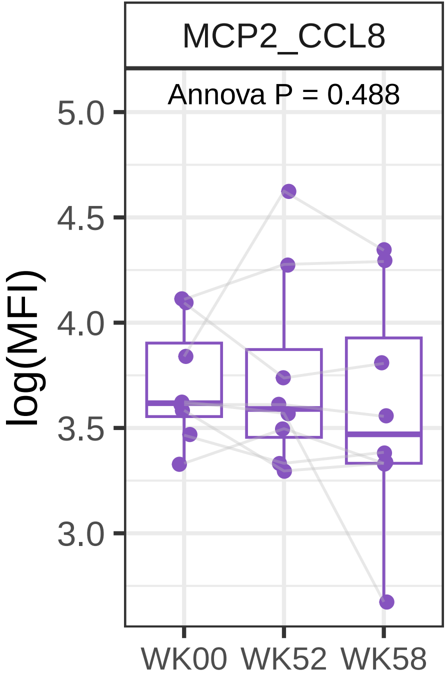
MCP2_CCL8
Annova P = 0.488
log(MFI)
5.0
4.5
4.0
3.5
3.0
WK00
WK52
WK58

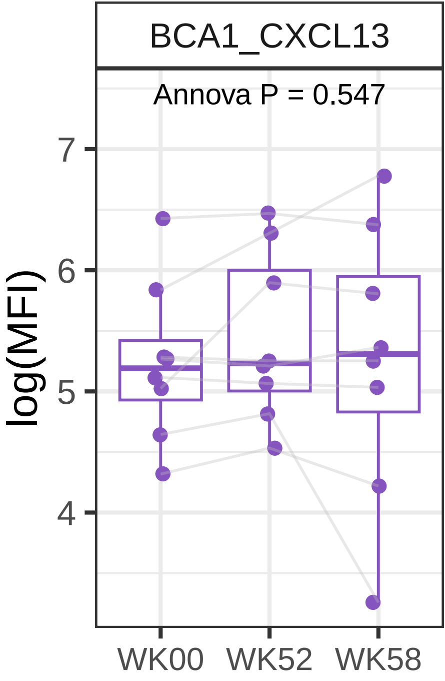
BCA1_CXCL13
Annova P = 0.547
log(MFI)
7
6
5
4
WK00
WK52
WK58

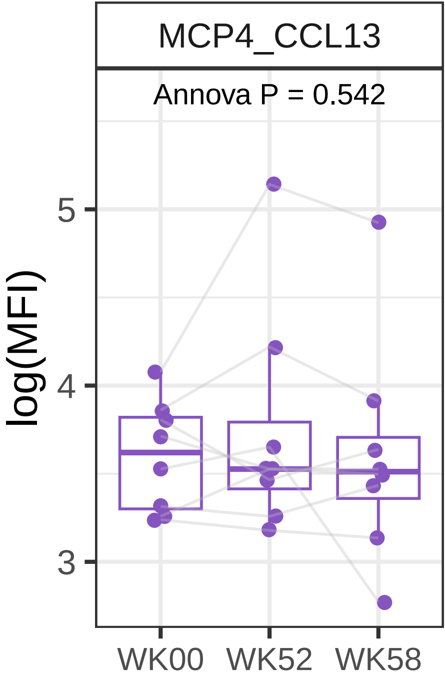

MCP4_CCL13
Annova P = 0.542
log(MFI)
5
4
3
WK00
WK52
WK58

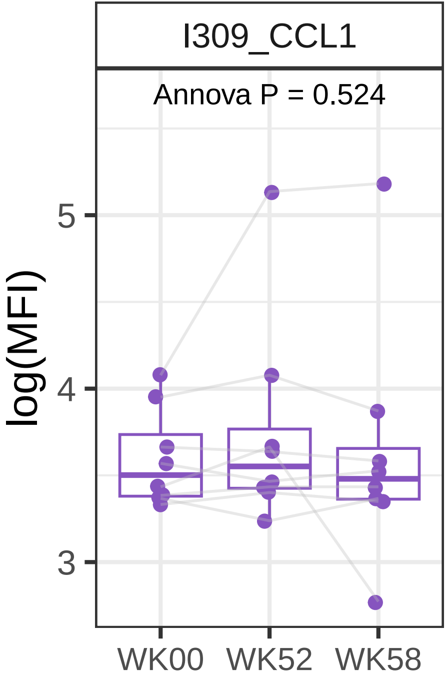

I309_CCL1
Annova P = 0.524
log(MFI)
5
4
3
WK00
WK52
WK58

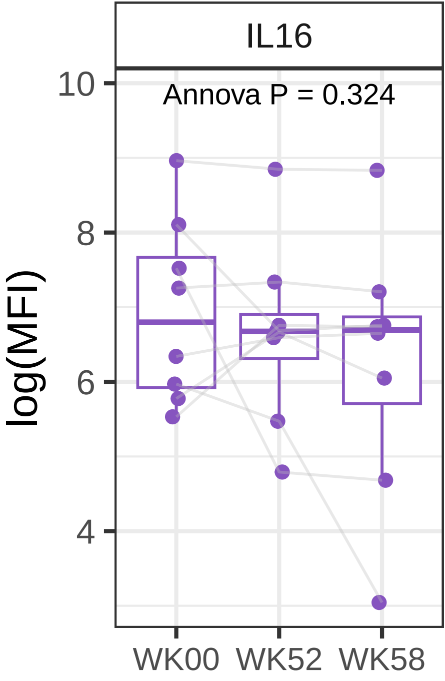
IL16
Annova P = 0.324
log(MFI)
10
8
6
4
WK00
WK52
WK58

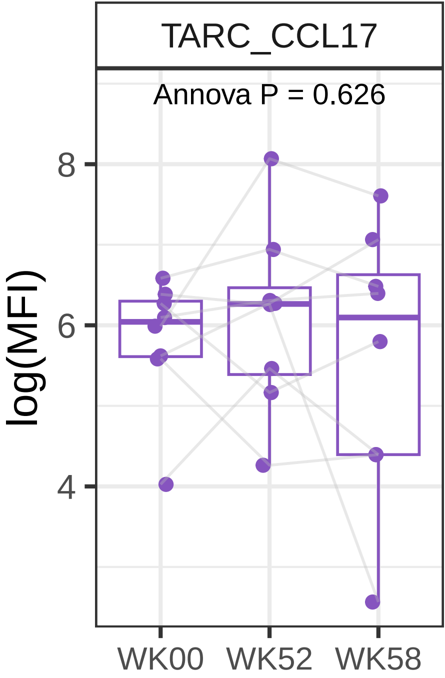

TARC_CCL17
Annova P = 0.626
log(MFI)
8
6
4
WK00
WK52
WK58

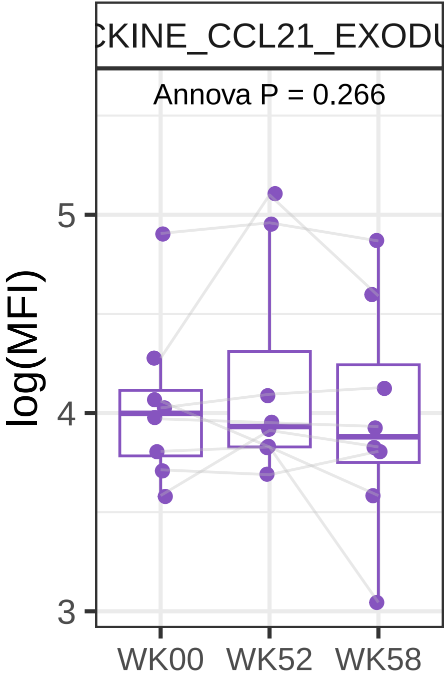

CKINE_CCL21_EXODU
Annova P = 0.266
log(MFI)
5
4
3
WK00
WK52
WK58

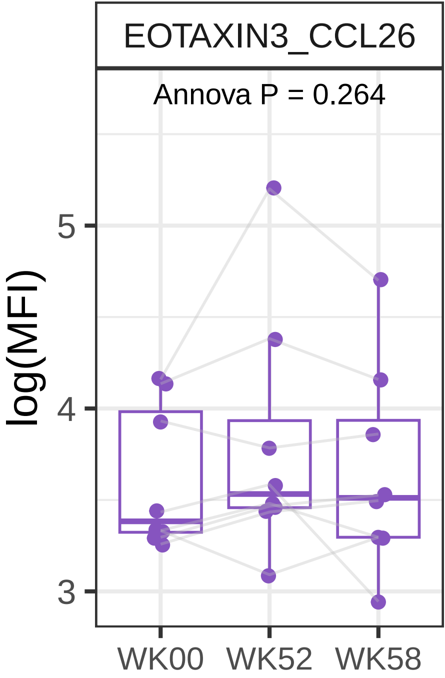

EOTAXIN3_CCL26
Annova P = 0.264
log(MFI)
5
4
3
WK00
WK52
WK58

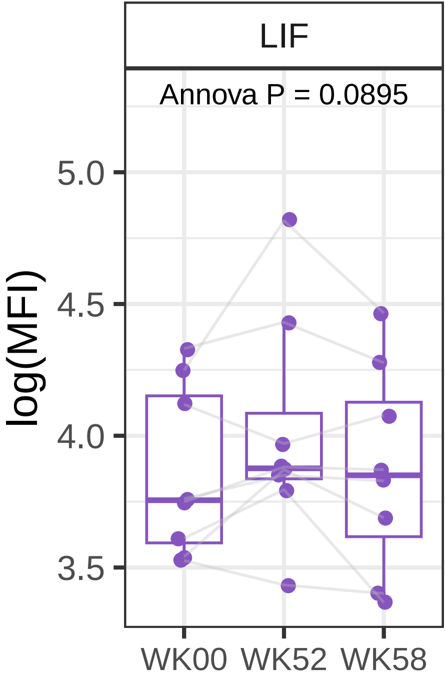
LIF
Annova P = 0.0895
log(MFI)
5.0
4.5
4.0
3.5
WK00
WK52
WK58

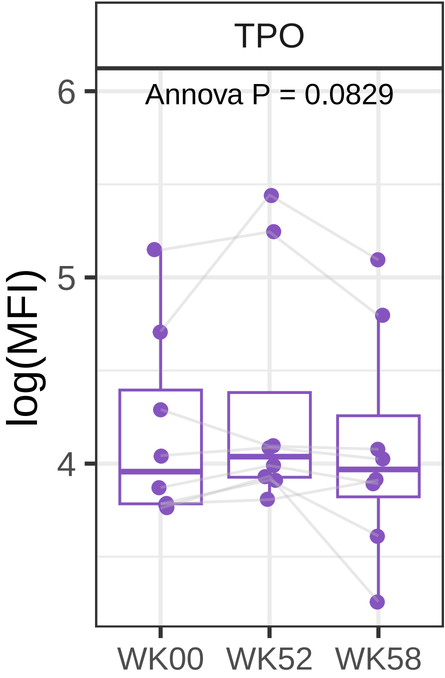
TPO
Annova P = 0.0829
log(MFI)
6
5
4
WK00
WK52
WK58

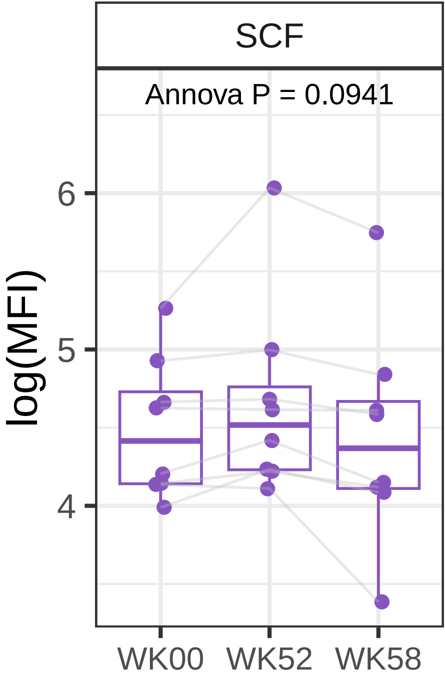

SCF
Annova P = 0.0941
log(MFI)
6
5
4
WK00
WK52
WK58

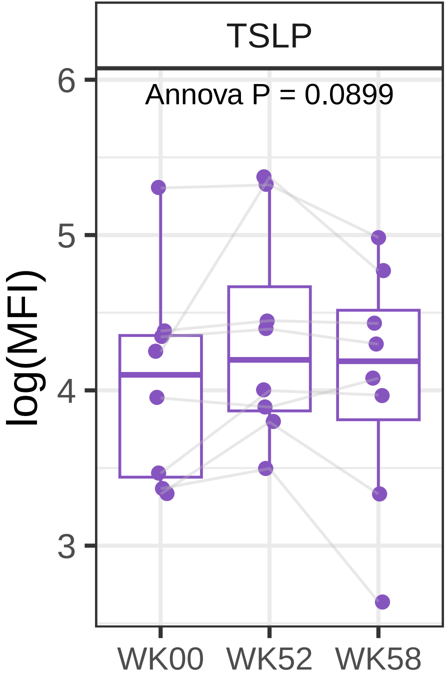
TSLP
Annova P = 0.0899
log(MFI)
6
5
4
3
WK00
WK52
WK58

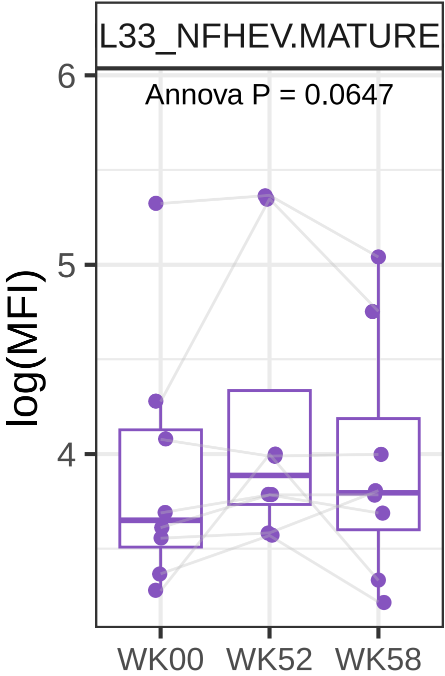

L33_NFHEV.MATURE
Annova P = 0.0647
log(MFI)
6
5
4
WK00
WK52
WK58

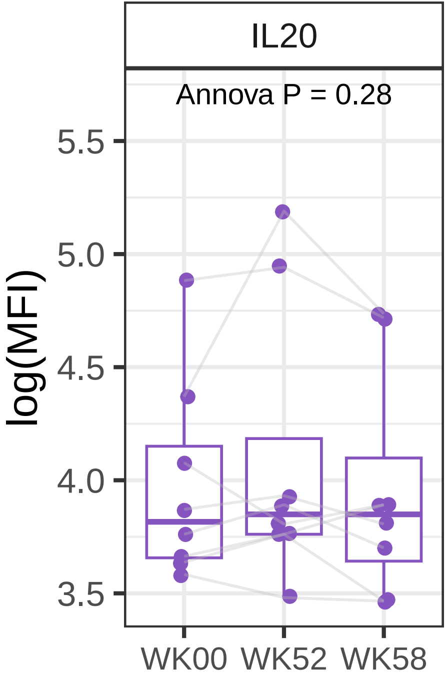
IL20
Annova P = 0.28
log(MFI)
5.5
5.0
4.5
4.0
3.5
WK00
WK52
WK58

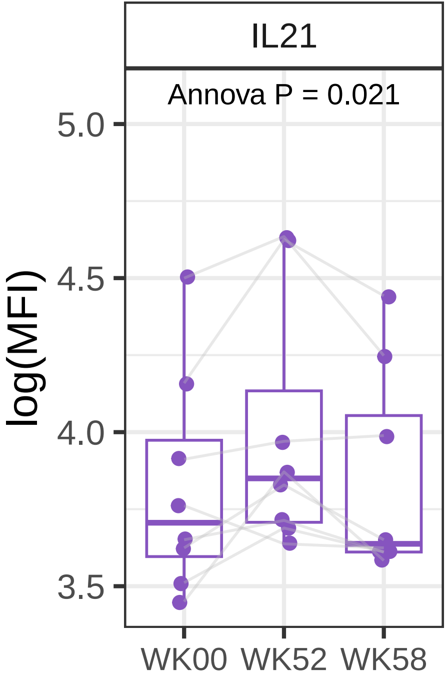
IL21
Annova P = 0.021
log(MFI)
5.0
4.5
4.0
3.5
WK00
WK52
WK58

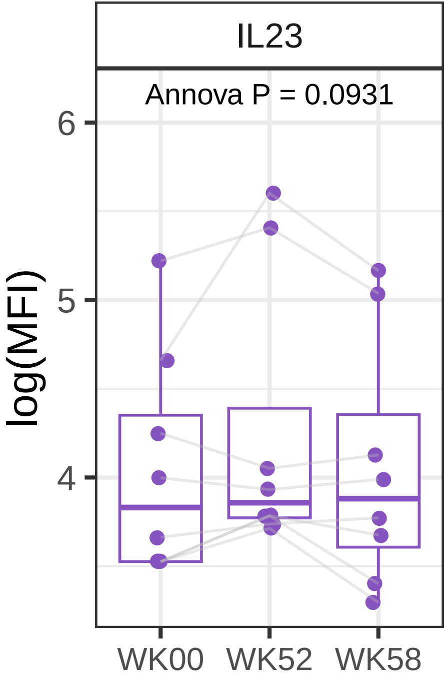
IL23
Annova P = 0.0931
log(MFI)
6
5
4
WK00
WK52
WK58

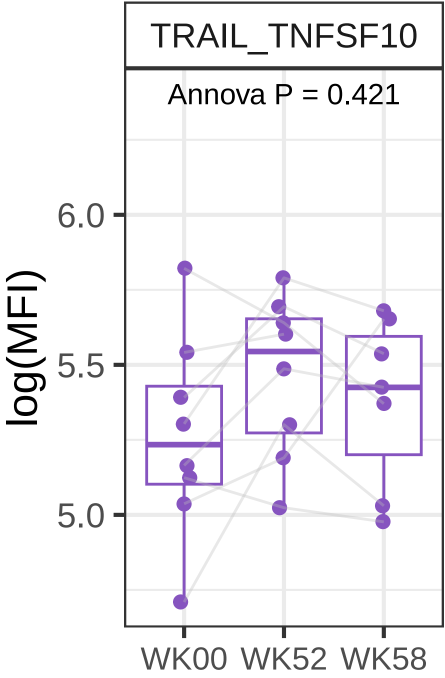

TRAIL_TNFSF10
Annova P = 0.421
log(MFI)
6.0
5.5
5.0
WK00
WK52
WK58

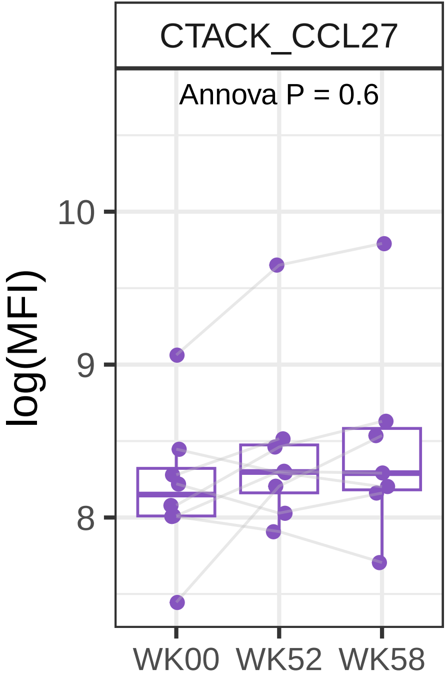
CTACK_CCL27
Annova P = 0.6
log(MFI)
10
9
8
WK00
WK52
WK58

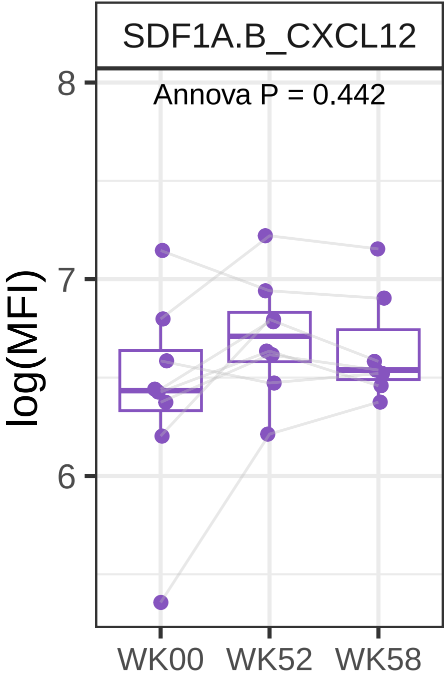

SDF1A.B_CXCL12
Annova P = 0.442
log(MFI)
8
7
6
WK00
WK52
WK58

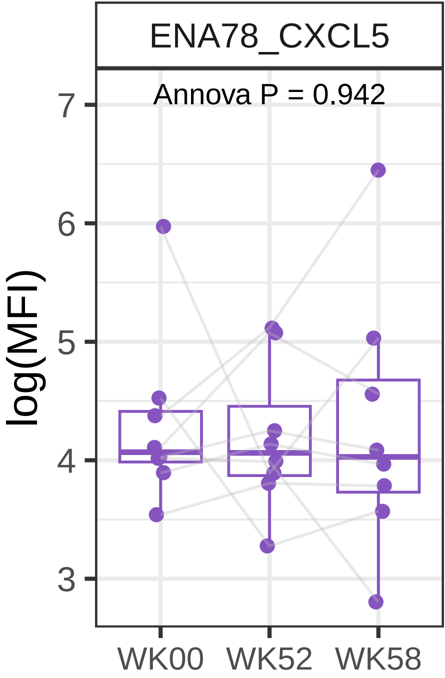
ENA78_CXCL5
Annova P = 0.942
log(MFI)
7
6
5
4
3
WK00
WK52
WK58

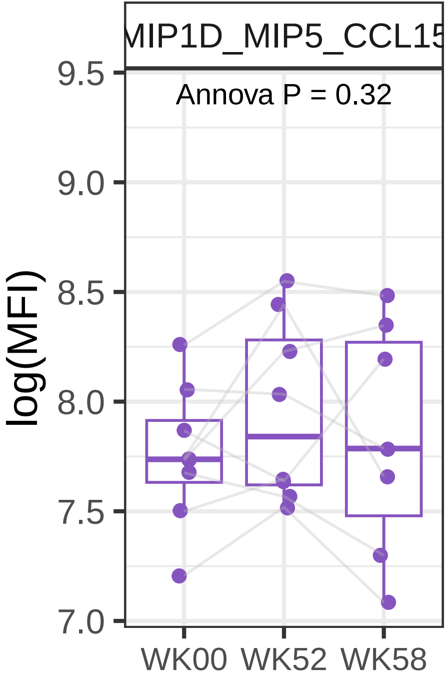

MIP1D_MIP5_CCL15
Annova P = 0.32
log(MFI)
9.5
9.0
8.5
8.0
7.5
7.0
WK00
WK52
WK58

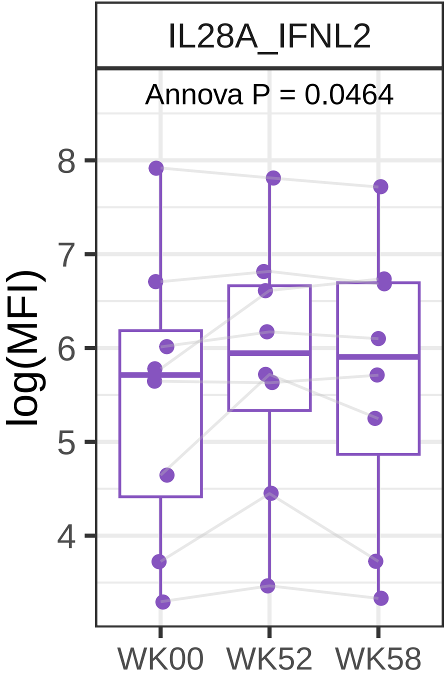
IL28A_IFNL2
Annova P = 0.0464
log(MFI)
8
7
6
5
4
WK00
WK52
WK58
