# Supplementary material for: Shrimp oral immunotherapy outcomes in the phase 2 clinical trial: MOTIF
Source: Front Allergy. 2025 Jul 22;6:1458131. doi: 10.3389/falgy.2025.1458131 (PMC12321884; doi:10.3389/falgy.2025.1458131)
Supplement: Supplementary Figure S3 — Luminex assay from unstimulated PBMC culture supernatants. [file Datasheet3.pdf]

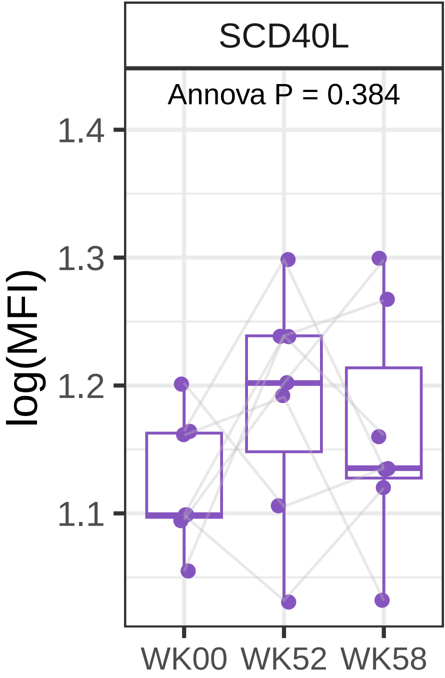
SCD40L
Annova P = 0.384
log(MFI)
1.4
1.3
1.2
1.1
WK00
WK52
WK58

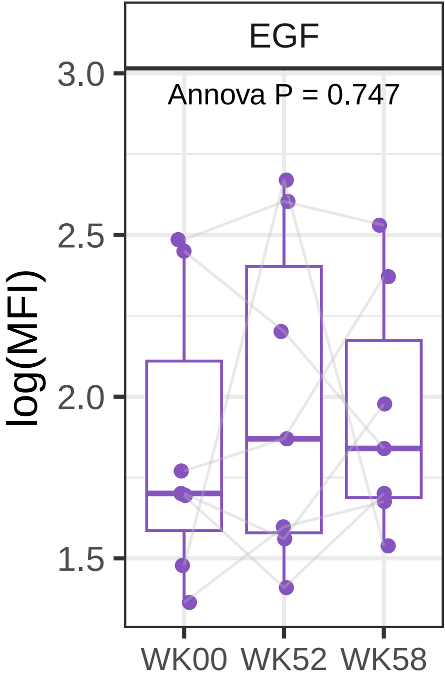
EGF
Annova P = 0.747
log(MFI)
3.0
2.5
2.0
1.5
WK00
WK52
WK58

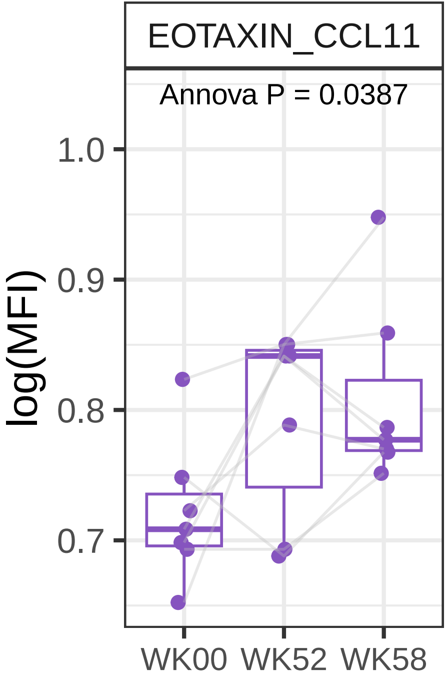
EOTAXIN_CCL11
Annova P = 0.0387
log(MFI)
1.0
0.9
0.8
0.7
WK00
WK52
WK58

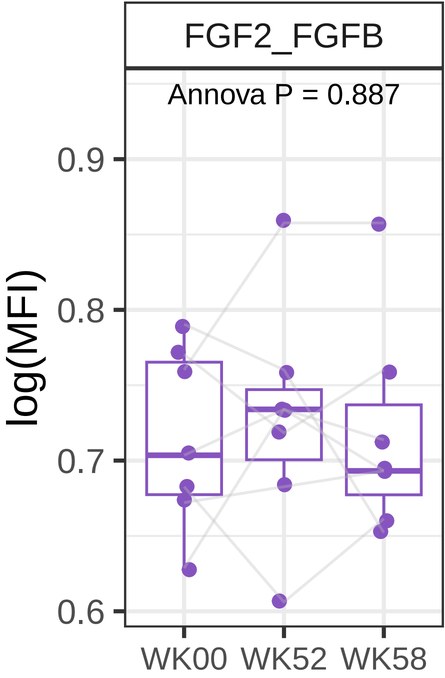

FGF2_FGFB
Annova P = 0.887
log(MFI)
0.9
0.8
0.7
0.6
WK00
WK52
WK58

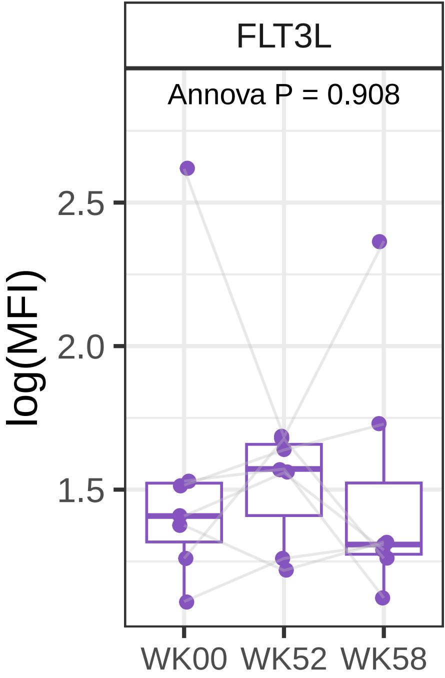
FLT3L
Annova P = 0.908
log(MFI)
2.5
2.0
1.5
WK00
WK52
WK58

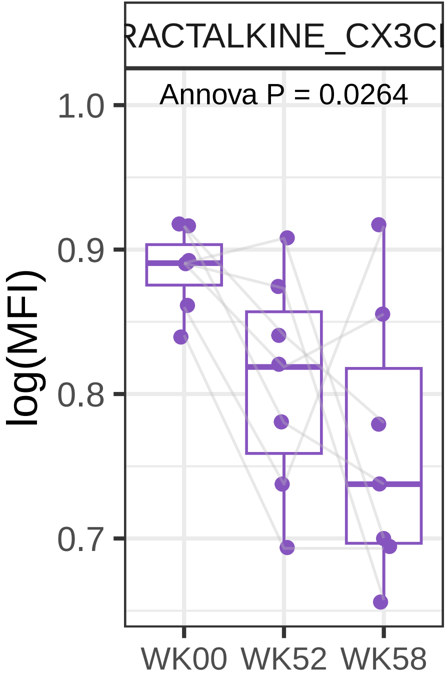

RACTALKINE_CX3CL
Annova P = 0.0264
log(MFI)
1.0
0.9
0.8
0.7
WK00
WK52
WK58

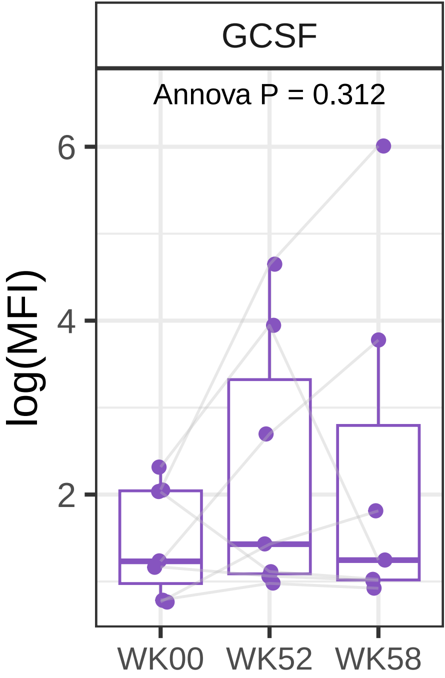
GCSF
Annova P = 0.312
log(MFI)
6
4
2
WK00
WK52
WK58

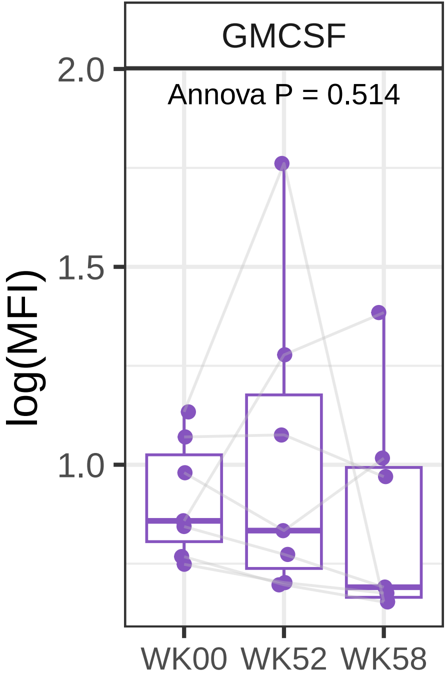
GMCSF
Annova P = 0.514
log(MFI)
2.0
1.5
1.0
WK00
WK52
WK58

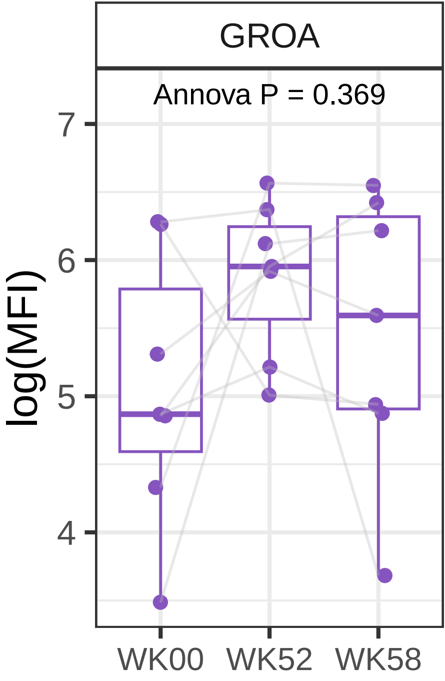

GROA
Annova P = 0.369
log(MFI)
7
6
5
4
WK00
WK52
WK58

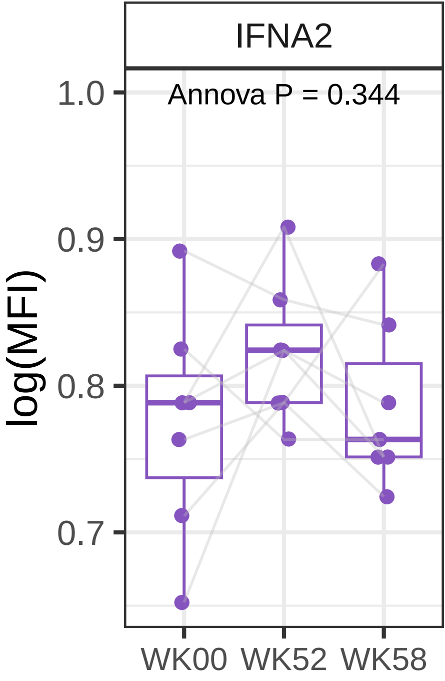
IFNA2
Annova P = 0.344
log(MFI)
1.0
0.9
0.8
0.7
WK00
WK52
WK58

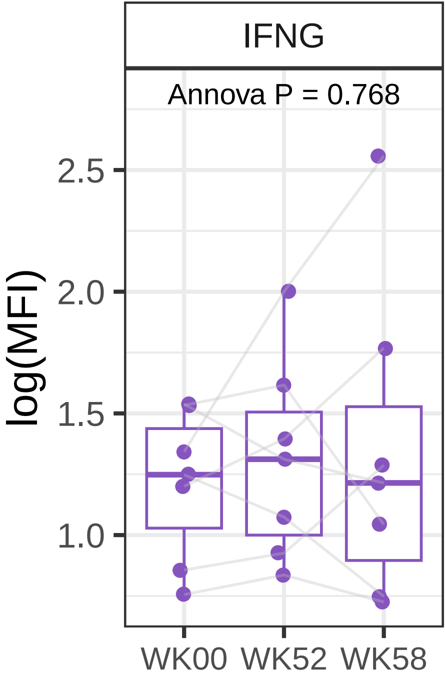

IFNG
Annova P = 0.768
log(MFI)
2.5
2.0
1.5
1.0
WK00
WK52
WK58

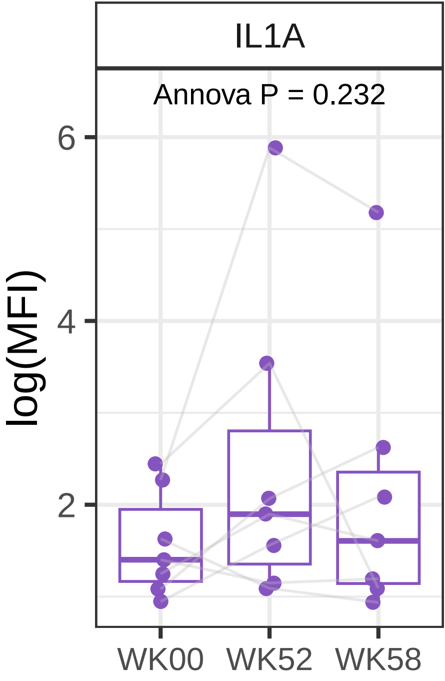
IL1A
Annova P = 0.232
log(MFI)
6
4
2
WK00
WK52
WK58

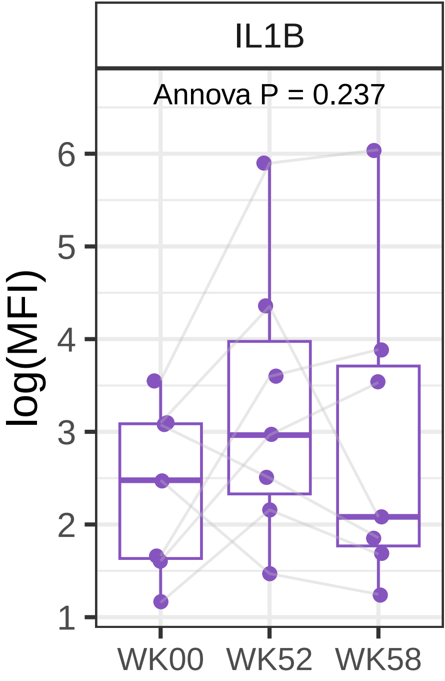
IL1B
Annova P = 0.237
log(MFI)
1
2
3
4
5
6
WK00
WK52
WK58

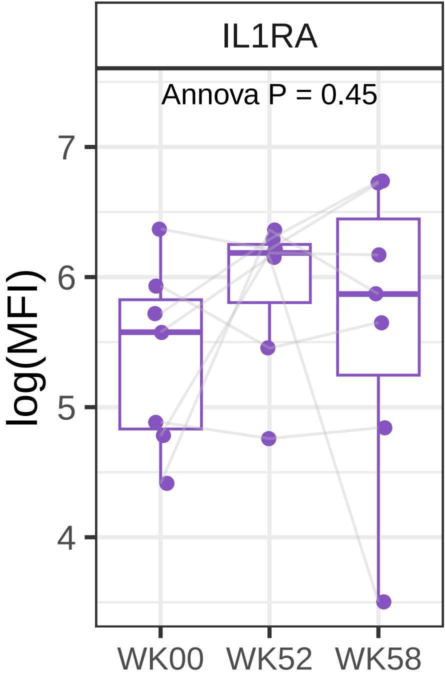
IL1RA
Annova P = 0.45
log(MFI)
7
6
5
4
WK00
WK52
WK58

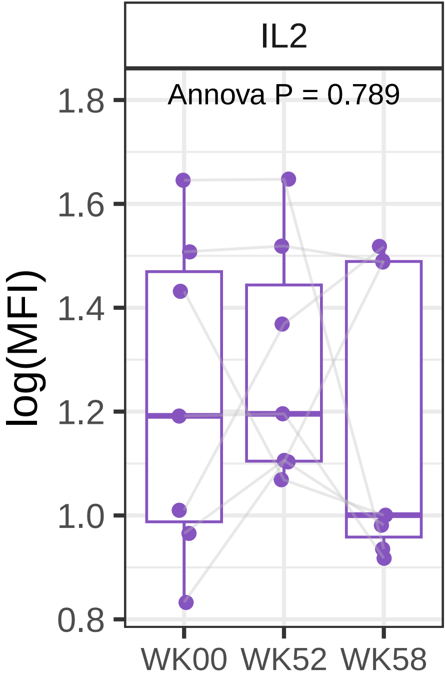
IL2
Annova P = 0.789
log(MFI)
1.8
1.6
1.4
1.2
1.0
0.8
WK00
WK52
WK58

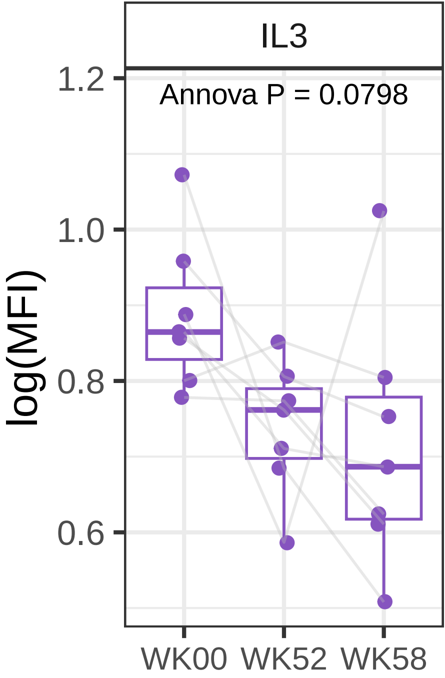
IL3
Annova P = 0.0798
log(MFI)
1.2
1.0
0.8
0.6
WK00
WK52
WK58

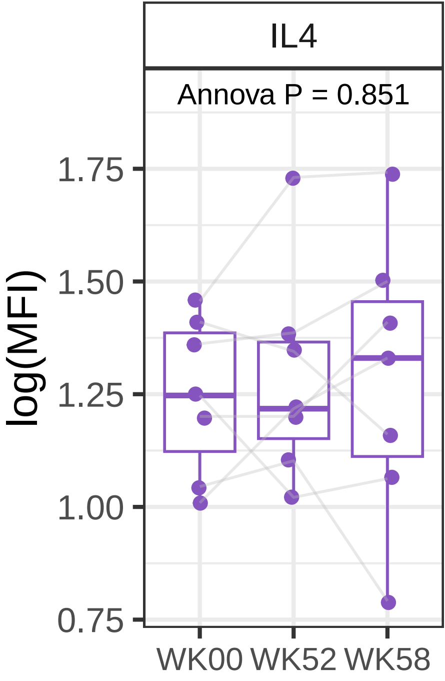
IL4
Annova P = 0.851
log(MFI)
1.75
1.50
1.25
1.00
0.75
WK00
WK52
WK58

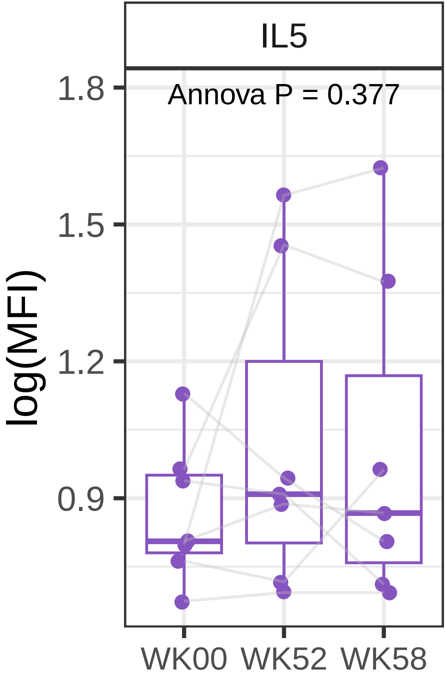
IL5
Annova P = 0.377
log(MFI)
1.8
1.5
1.2
0.9
WK00
WK52
WK58

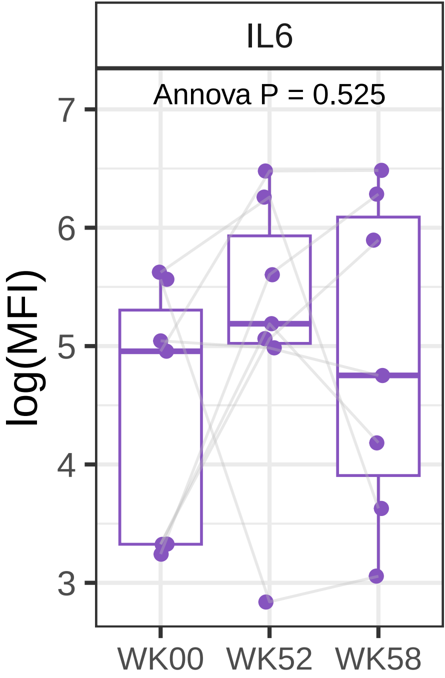
IL6
Annova P = 0.525
log(MFI)
7
6
5
4
3
WK00
WK52
WK58

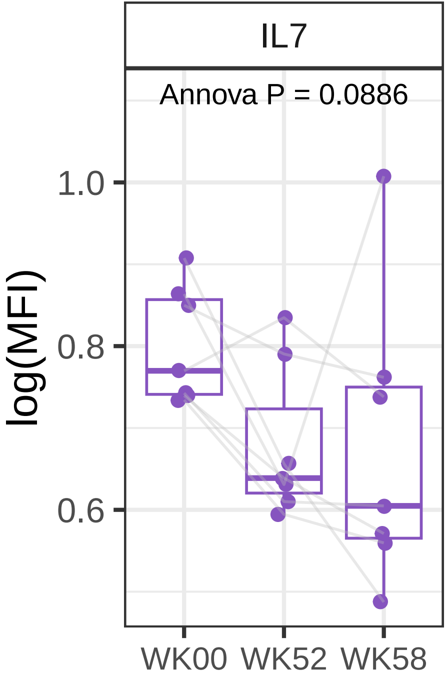
IL7
Annova P = 0.0886
log(MFI)
1.0
0.8
0.6
WK00
WK52
WK58

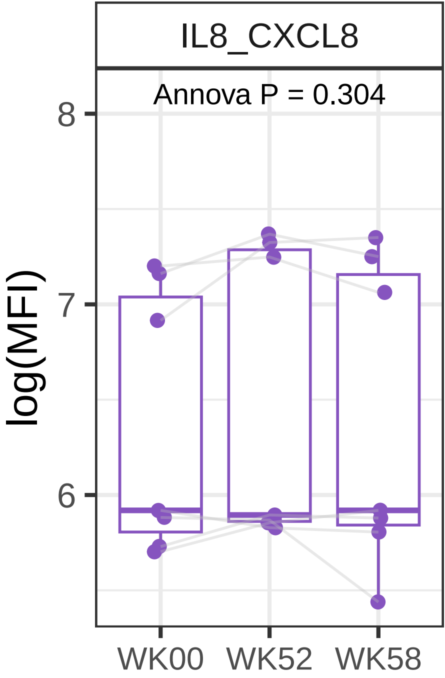

IL8_CXCL8
Annova P = 0.304
log(MFI)
8
7
6
WK00
WK52
WK58

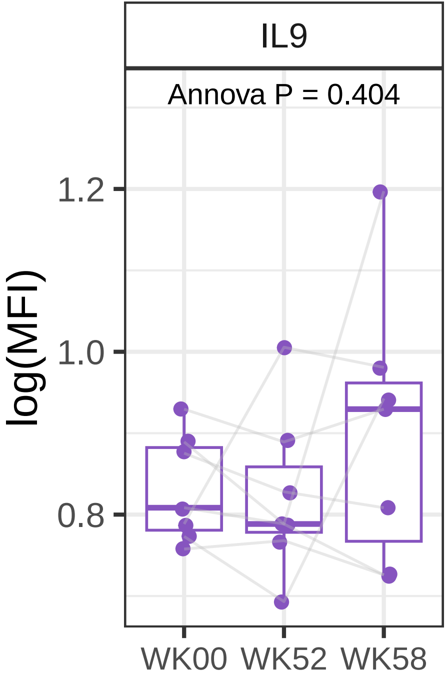

IL9
Annova P = 0.404
log(MFI)
1.2
1.0
0.8
WK00
WK52
WK58

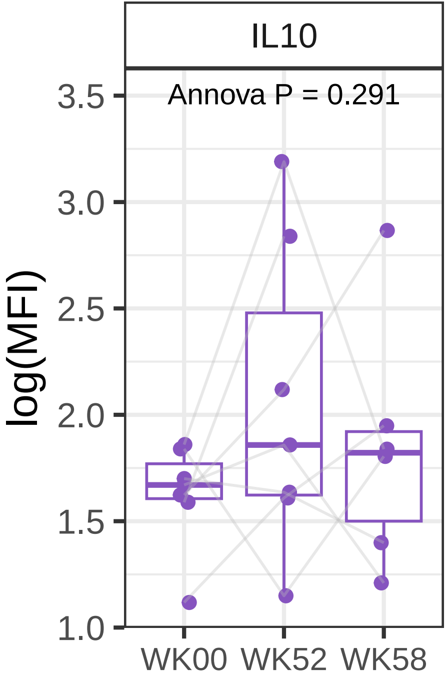
IL10
Annova P = 0.291
log(MFI)
3.5
3.0
2.5
2.0
1.5
1.0
WK00
WK52
WK58

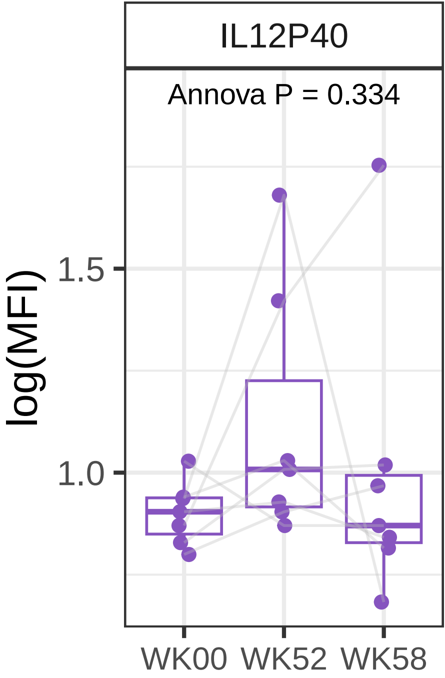
IL12P40
Annova P = 0.334
log(MFI)
1.5
1.0
WK00
WK52
WK58

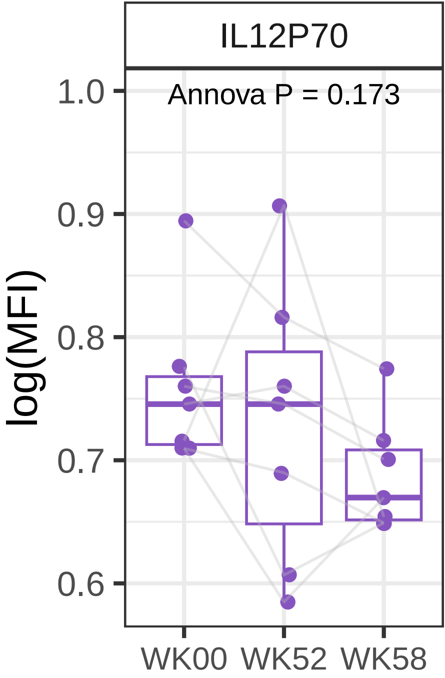
IL12P70
Annova P = 0.173
log(MFI)
1.0
0.9
0.8
0.7
0.6
WK00
WK52
WK58

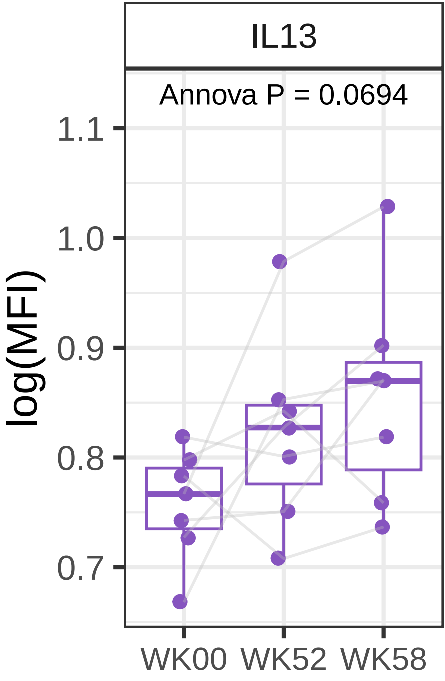
IL13
Annova P = 0.0694
log(MFI)
1.1
1.0
0.9
0.8
0.7
WK00
WK52
WK58

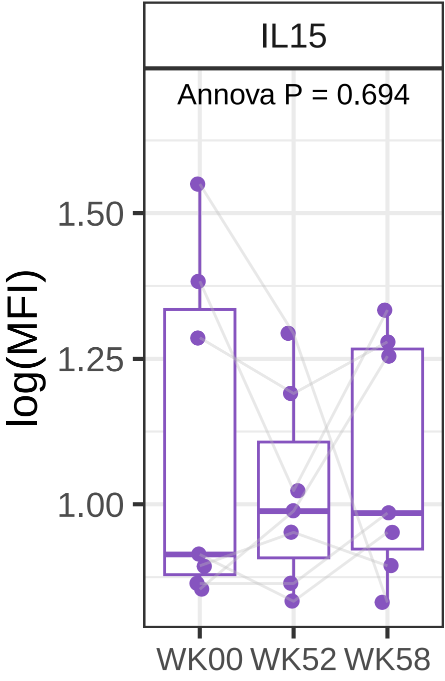

IL15
Annova P = 0.694
log(MFI)
1.50
1.25
1.00
WK00
WK52
WK58

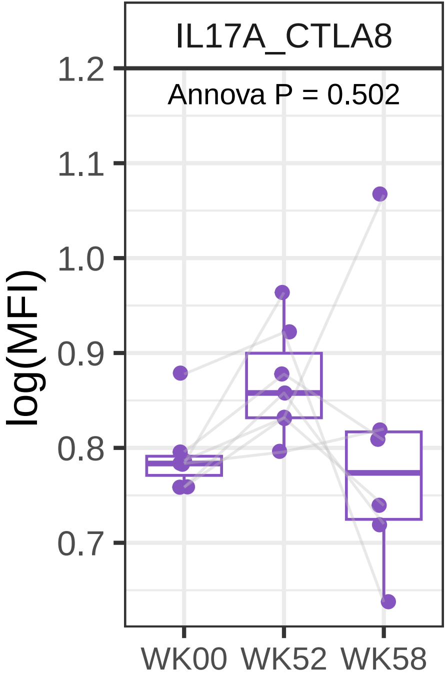
IL17A_CTLA8
Annova P = 0.502
log(MFI)
1.2
1.1
1.0
0.9
0.8
0.7
WK00
WK52
WK58

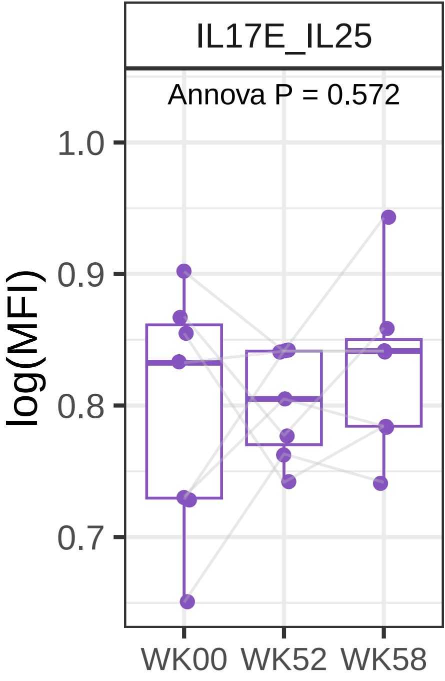
IL17E_IL25
Annova P = 0.572
log(MFI)
1.0
0.9
0.8
0.7
WK00
WK52
WK58

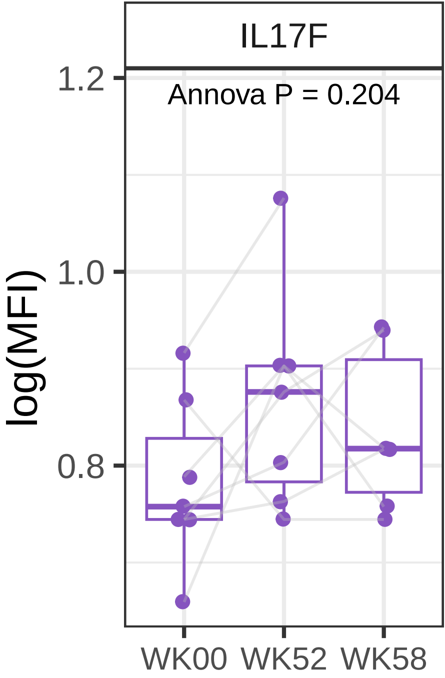
IL17F
Annova P = 0.204
log(MFI)
1.2
1.0
0.8
WK00
WK52
WK58

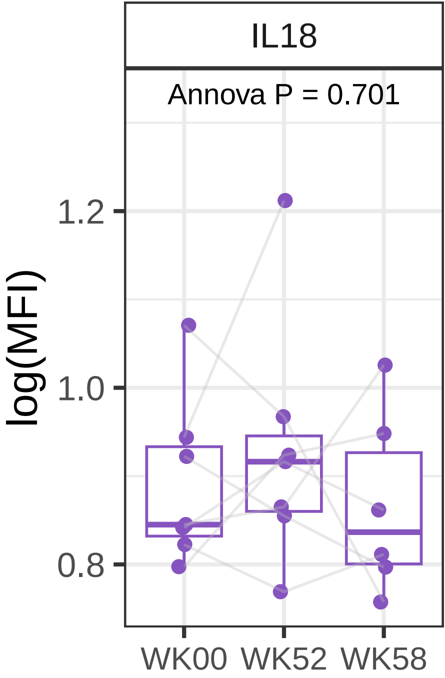
IL18
Annova P = 0.701
log(MFI)
1.2
1.0
0.8
WK00
WK52
WK58

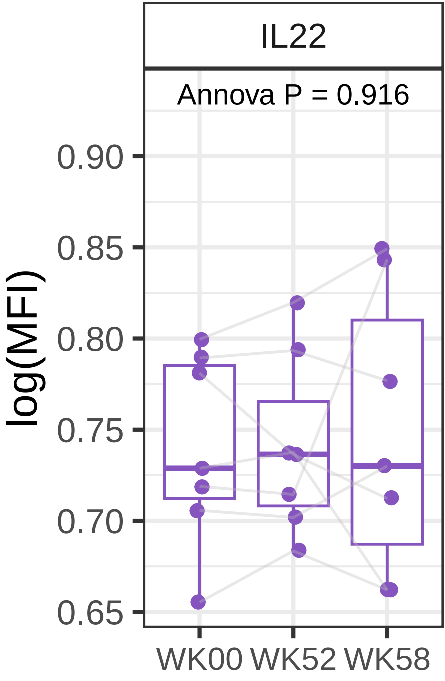
IL22
Annova P = 0.916
0.90
0.85
0.80
0.75
0.70
0.65
log(MFI)
WK00
WK52
WK58

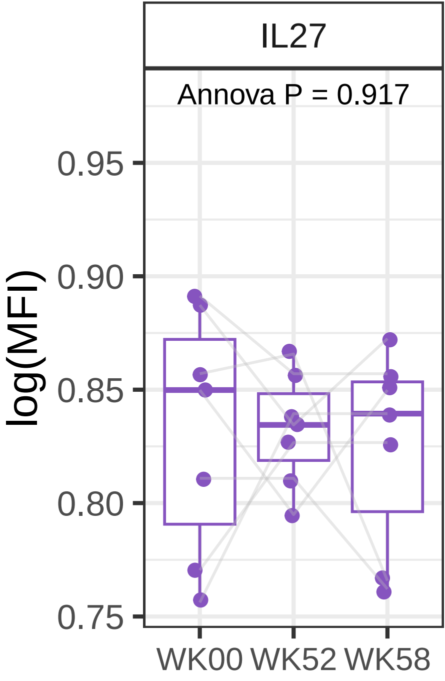

IL27
Annova P = 0.917
log(MFI)
0.95
0.90
0.85
0.80
0.75
WK00
WK52
WK58

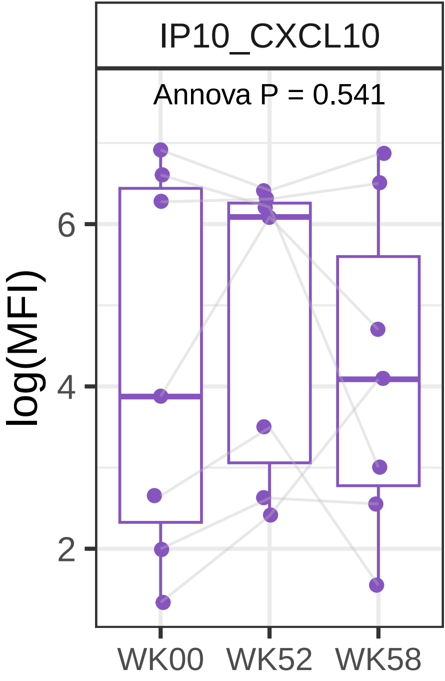
IP10_CXCL10
Annova P = 0.541
log(MFI)
6
4
2
WK00
WK52
WK58

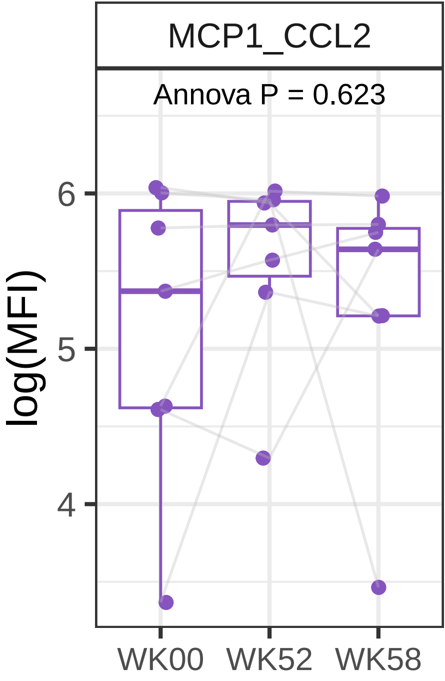

MCP1_CCL2
Annova P = 0.623
log(MFI)
6
5
4
WK00
WK52
WK58

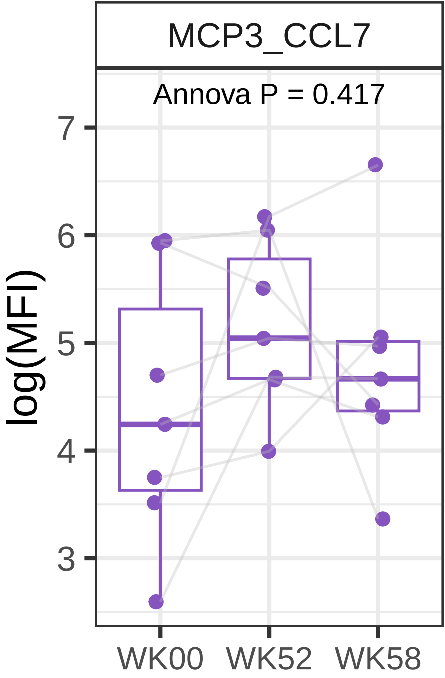
MCP3_CCL7
Annova P = 0.417
log(MFI)
7
6
5
4
3
WK00
WK52
WK58

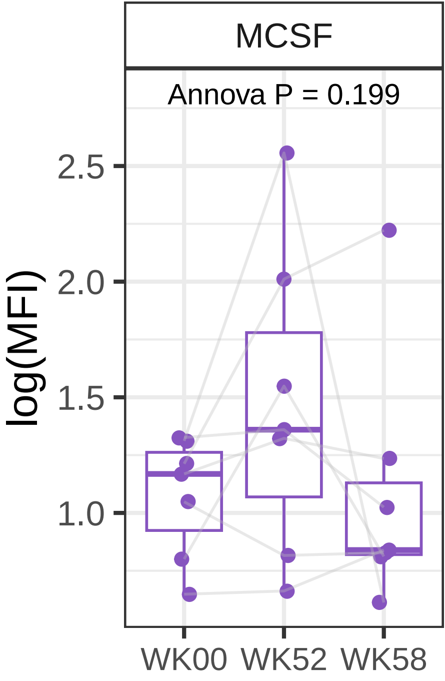
MCSF
Annova P = 0.199
log(MFI)
2.5
2.0
1.5
1.0
WK00
WK52
WK58

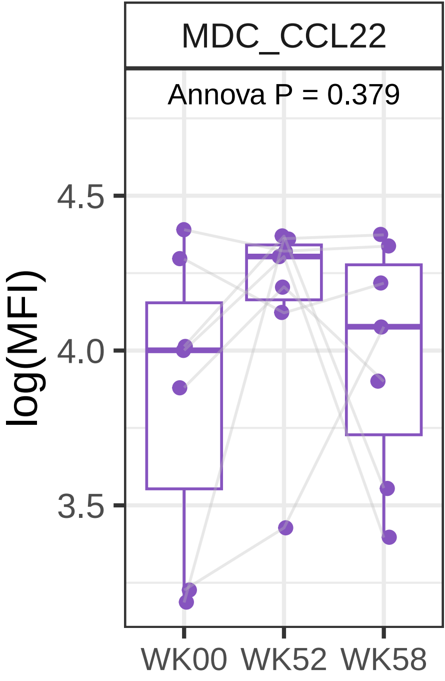
MDC_CCL22
Annova P = 0.379
log(MFI)
4.5
4.0
3.5
WK00
WK52
WK58

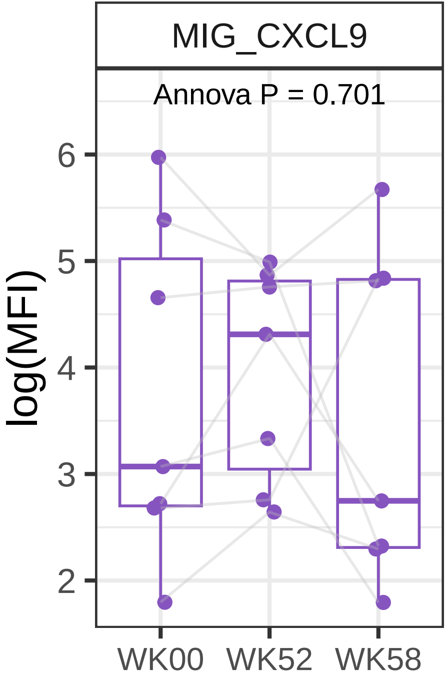
MIG_CXCL9
Annova P = 0.701
log(MFI)
6
5
4
3
2
WK00
WK52
WK58

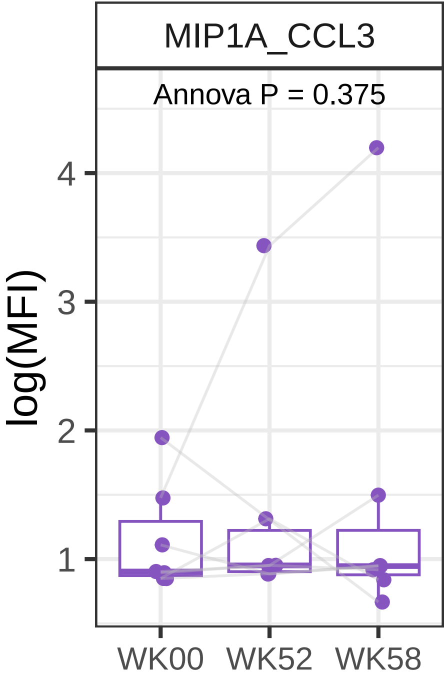

MIP1A_CCL3
Annova P = 0.375
log(MFI)
1
2
3
4
WK00
WK52
WK58

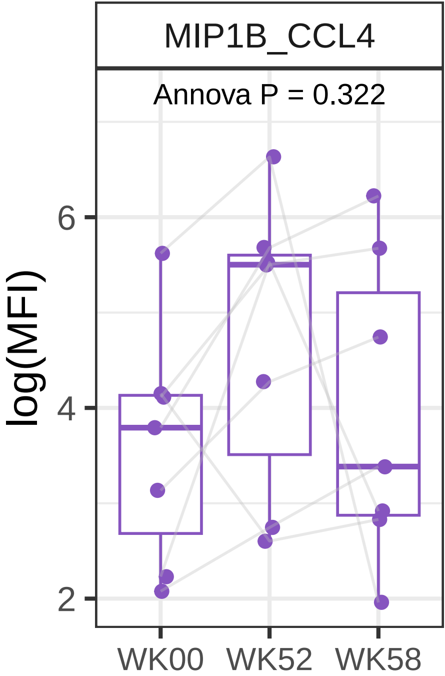
MIP1B_CCL4
Annova P = 0.322
log(MFI)
6
4
2
WK00
WK52
WK58

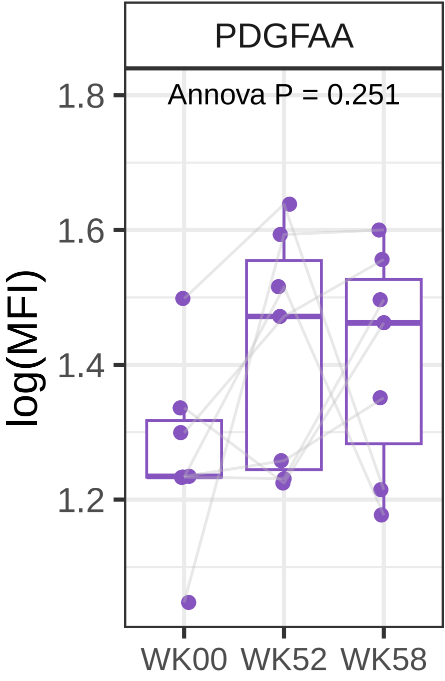
PDGFAA
Annova P = 0.251
log(MFI)
1.8
1.6
1.4
1.2
WK00
WK52
WK58

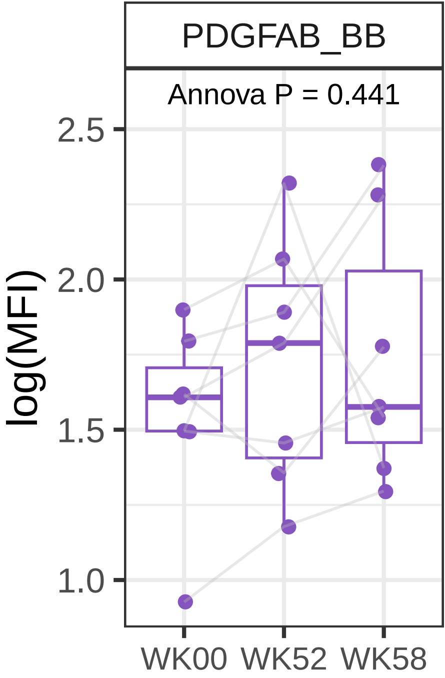
PDGFAB_BB
Annova P = 0.441
log(MFI)
2.5
2.0
1.5
1.0
WK00
WK52
WK58

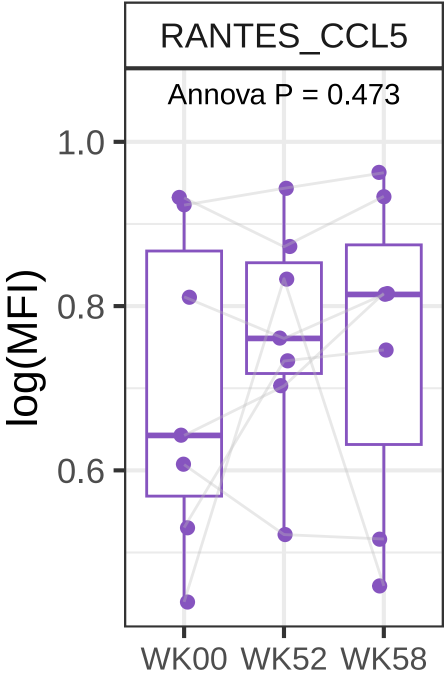
RANTES_CCL5
Annova P = 0.473
log(MFI)
1.0
0.8
0.6
WK00
WK52
WK58

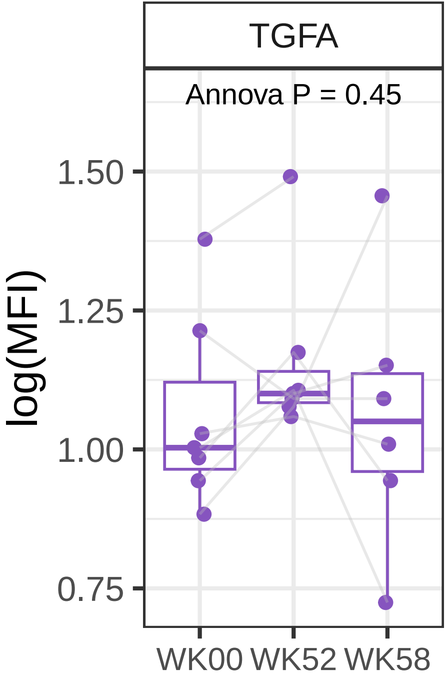
TGFA
Annova P = 0.45
log(MFI)
1.50
1.25
1.00
0.75
WK00
WK52
WK58

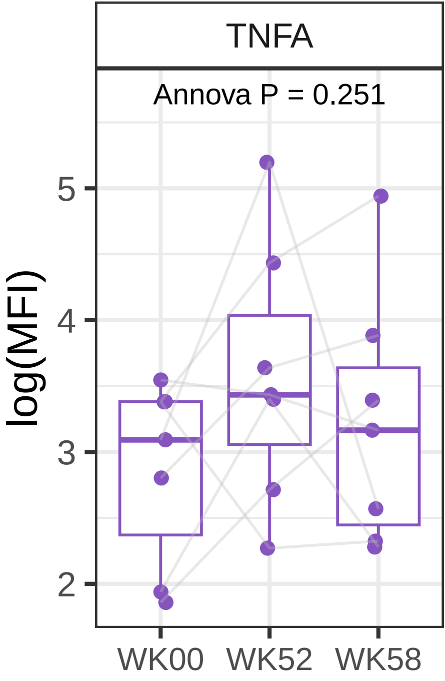
TNFA
Annova P = 0.251
log(MFI)
5
4
3
2
WK00
WK52
WK58

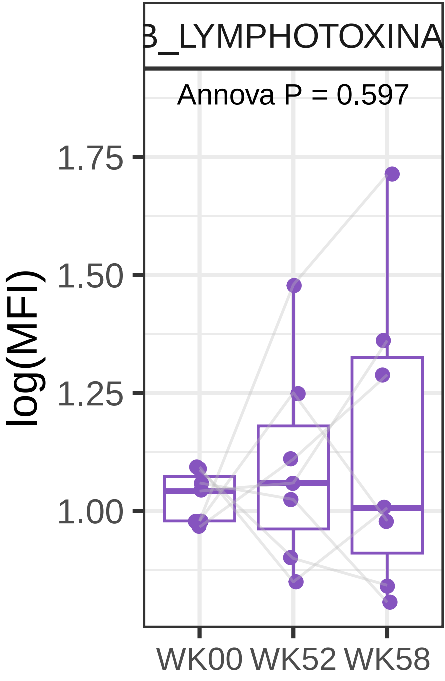

B_LYMPHOTOXINA
Annova P = 0.597
log(MFI)
1.75
1.50
1.25
1.00
WK00
WK52
WK58

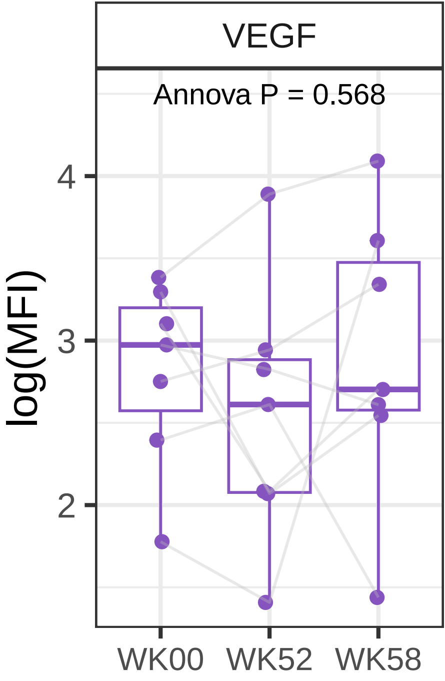

VEGF
Annova P = 0.568
log(MFI)
4
3
2
WK00
WK52
WK58
