# Supplementary material for: Shrimp oral immunotherapy outcomes in the phase 2 clinical trial: MOTIF
Source: Front Allergy. 2025 Jul 22;6:1458131. doi: 10.3389/falgy.2025.1458131 (PMC12321884; doi:10.3389/falgy.2025.1458131)
Supplement: Supplementary Figure S4 — Luminex assay from shrimp-stimulated PBMC culture supernatants. [file Datasheet4.pdf]

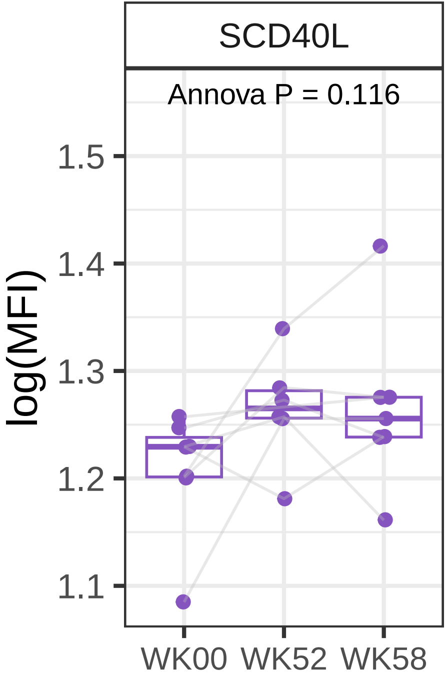
SCD40L
Annova P = 0.116
log(MFI)
1.5
1.4
1.3
1.2
1.1
WK00
WK52
WK58

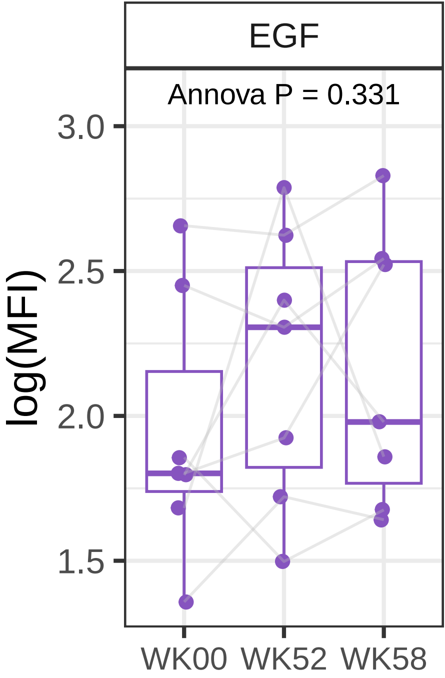
EGF
Annova P = 0.331
log(MFI)
3.0
2.5
2.0
1.5
WK00
WK52
WK58

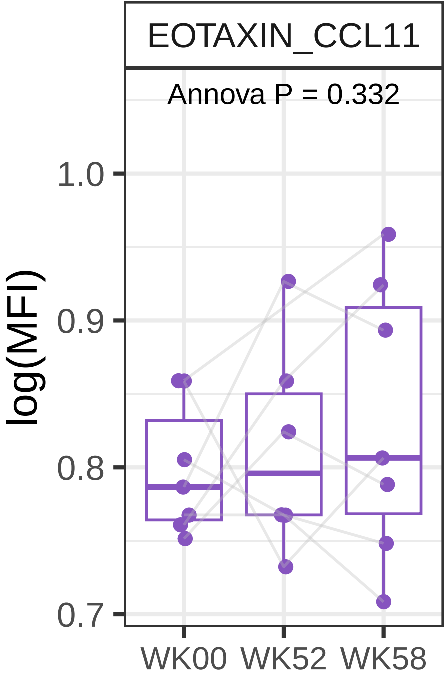
EOTAXIN_CCL11
Annova P = 0.332
log(MFI)
1.0
0.9
0.8
0.7
WK00
WK52
WK58

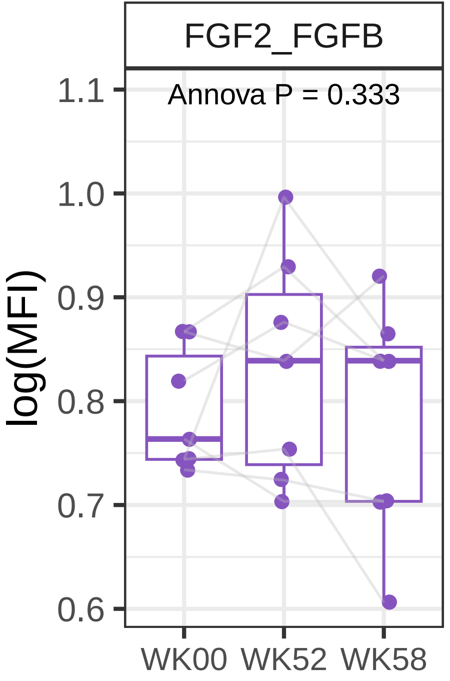
FGF2_FGFB
Annova P = 0.333
log(MFI)
1.1
1.0
0.9
0.8
0.7
0.6
WK00
WK52
WK58

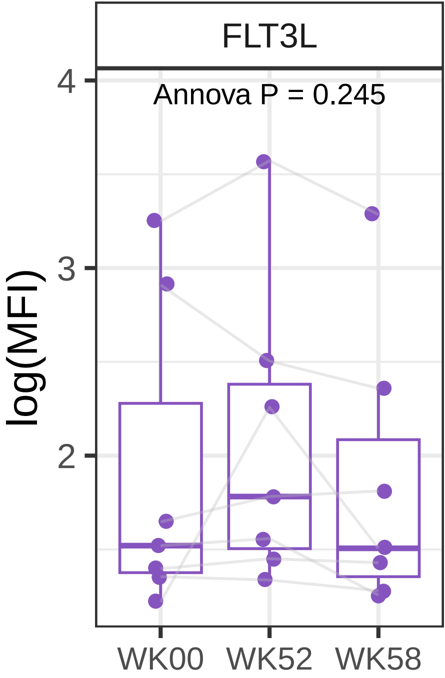
FLT3L
Annova P = 0.245
log(MFI)
4
3
2
WK00
WK52
WK58

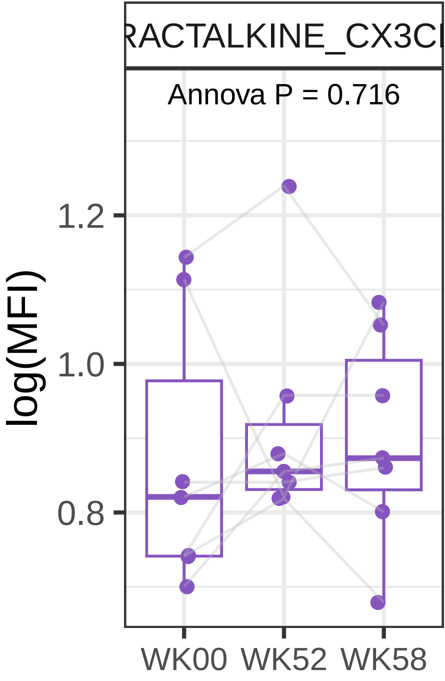
RACTALKINE_CX3CL
Annova P = 0.716
log(MFI)
1.2
1.0
0.8
WK00
WK52
WK58

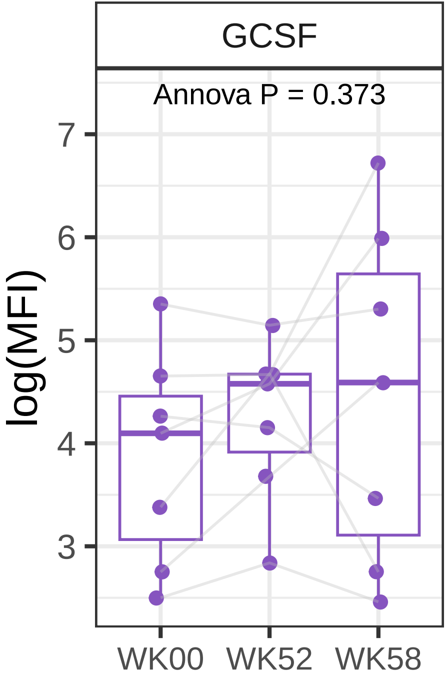
GCSF
Annova P = 0.373
log(MFI)
7
6
5
4
3
WK00
WK52
WK58

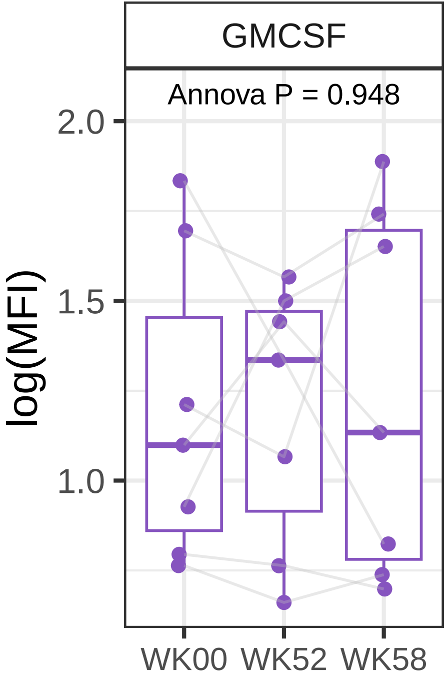

GMCSF
Annova P = 0.948
log(MFI)
2.0
1.5
1.0
WK00
WK52
WK58

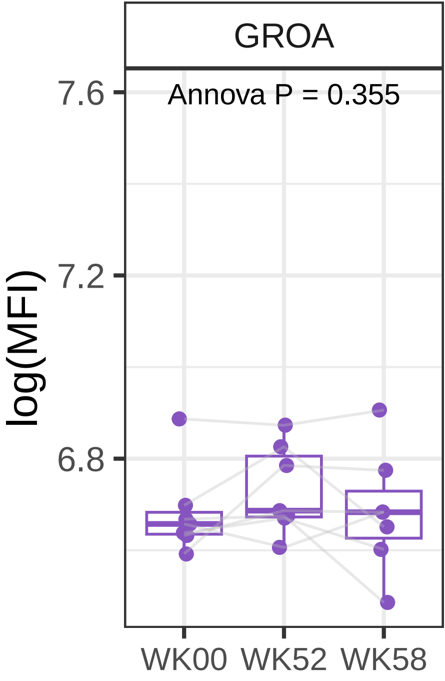
GROA
Annova P = 0.355
log(MFI)
7.6
7.2
6.8
WK00
WK52
WK58

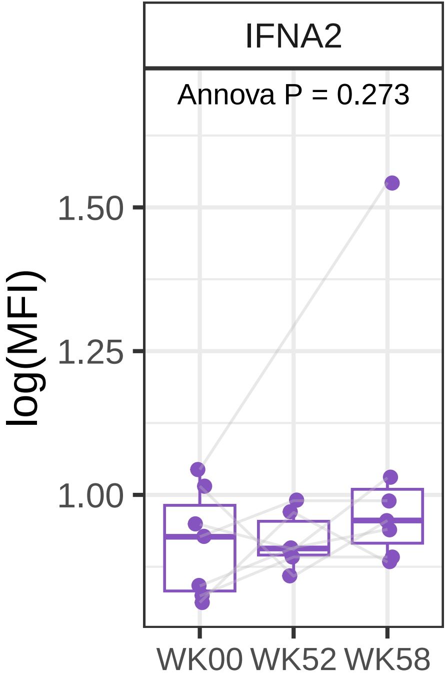
IFNA2
Annova P = 0.273
log(MFI)
1.50
1.25
1.00
WK00
WK52
WK58

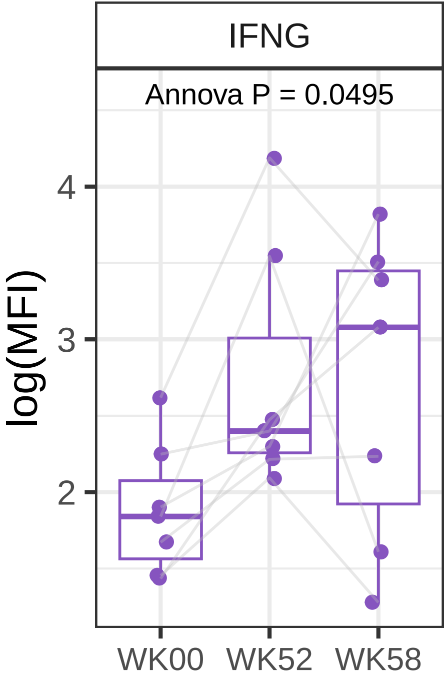
IFNG
Annova P = 0.0495
log(MFI)
4
3
2
WK00
WK52
WK58

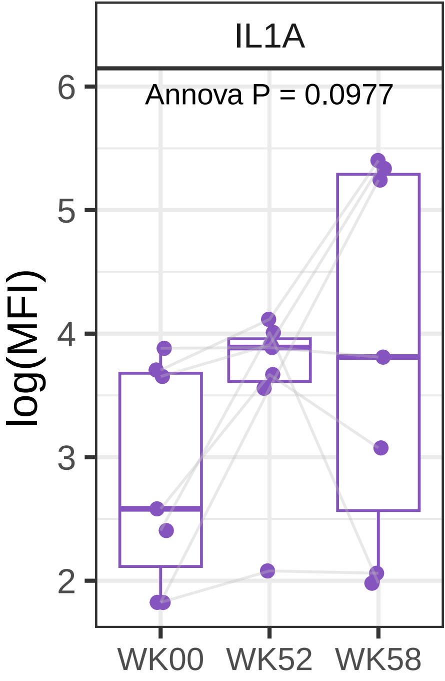
IL1A
Annova P = 0.0977
log(MFI)
6
5
4
3
2
WK00
WK52
WK58

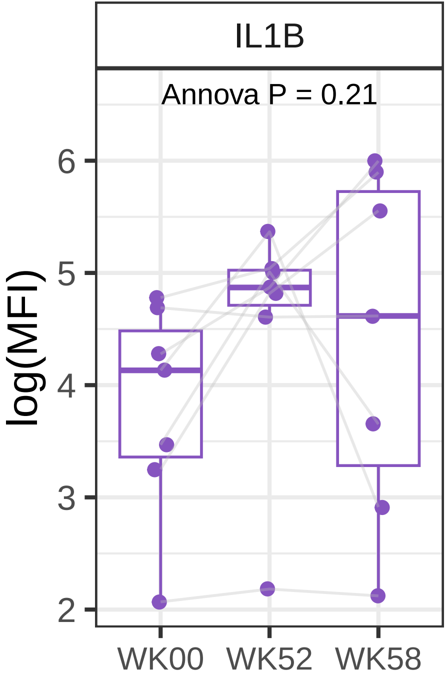
IL1B
Annova P = 0.21
log(MFI)
6
5
4
3
2
WK00
WK52
WK58

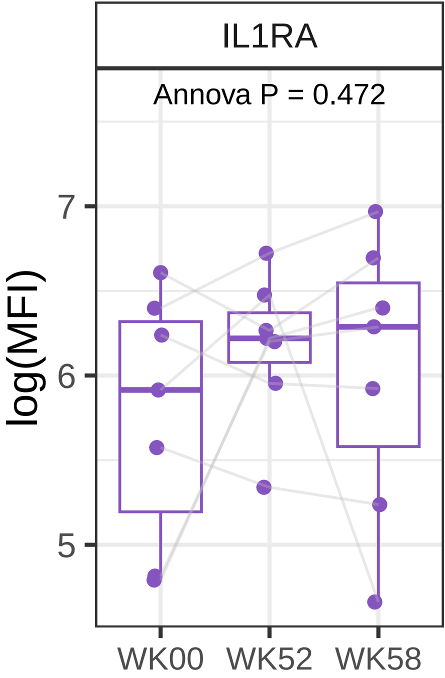
IL1RA
Annova P = 0.472
log(MFI)
7
6
5
WK00
WK52
WK58

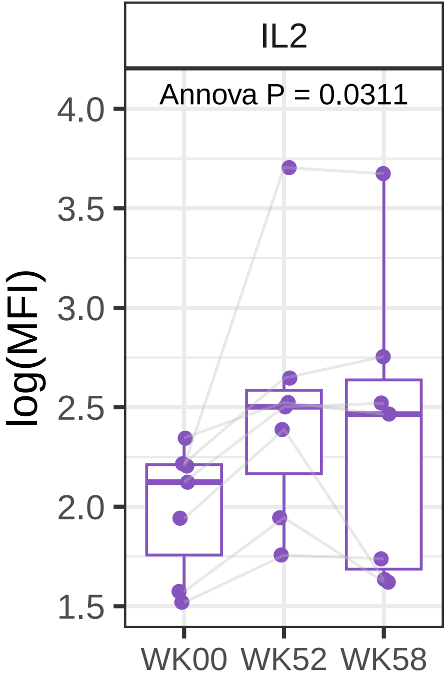

IL2
Annova P = 0.0311
log(MFI)
4.0
3.5
3.0
2.5
2.0
1.5
WK00
WK52
WK58

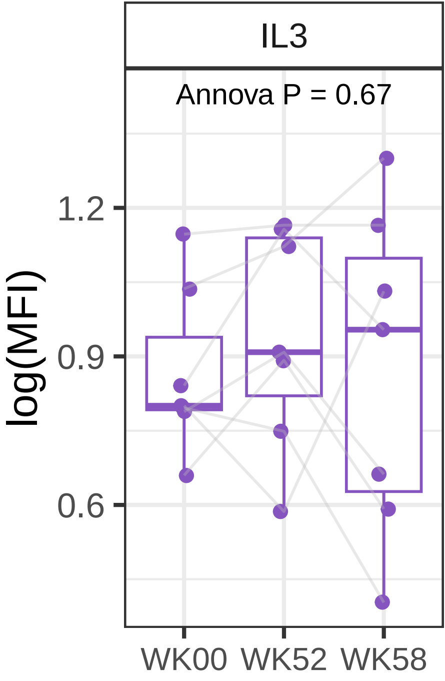
IL3
Annova P = 0.67
log(MFI)
1.2
0.9
0.6
WK00
WK52
WK58

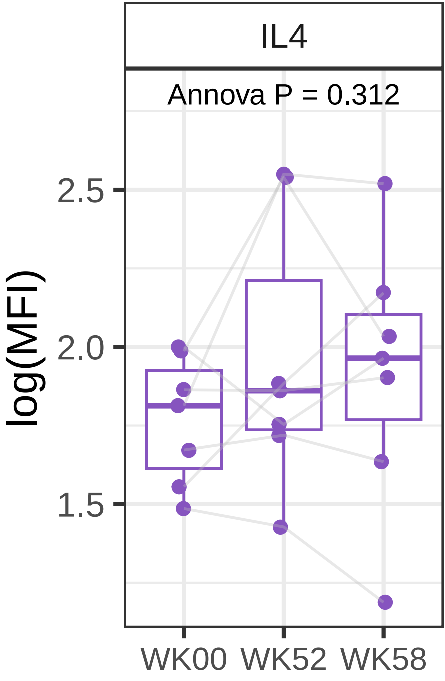

IL4
Annova P = 0.312
log(MFI)
2.5
2.0
1.5
WK00
WK52
WK58

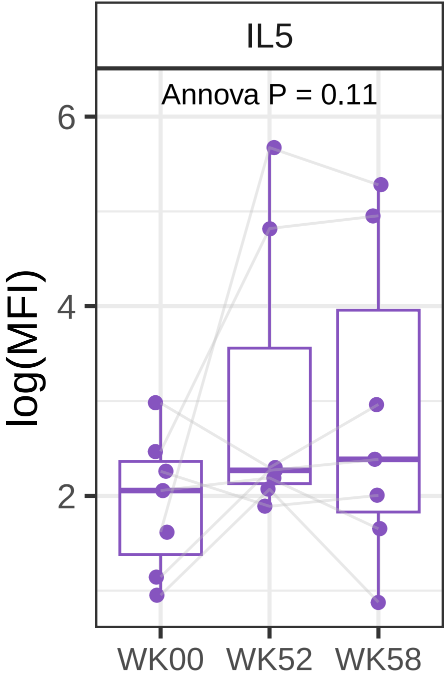
IL5
Annova P = 0.11
log(MFI)
6
4
2
WK00
WK52
WK58

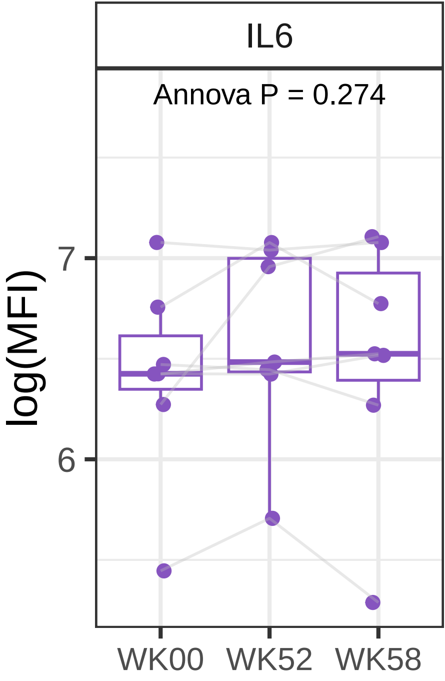
IL6
Annova P = 0.274
log(MFI)
7
6
WK00
WK52
WK58

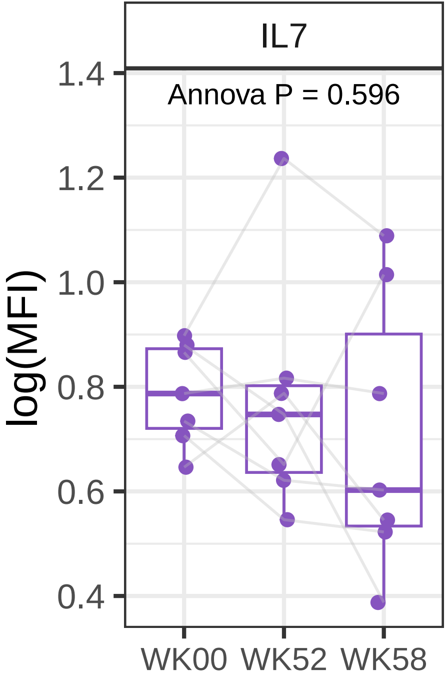
IL7
Annova P = 0.596
log(MFI)
1.4
1.2
1.0
0.8
0.6
0.4
WK00
WK52
WK58

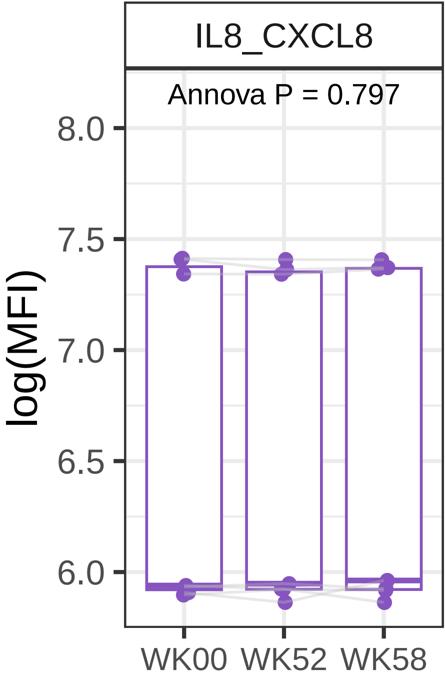
IL8_CXCL8
Annova P = 0.797
log(MFI)
8.0
7.5
7.0
6.5
6.0
WK00
WK52
WK58

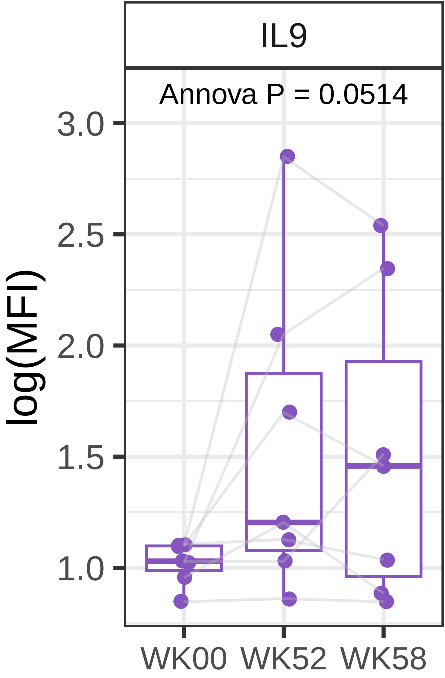
IL9
Annova P = 0.0514
log(MFI)
3.0
2.5
2.0
1.5
1.0
WK00
WK52
WK58

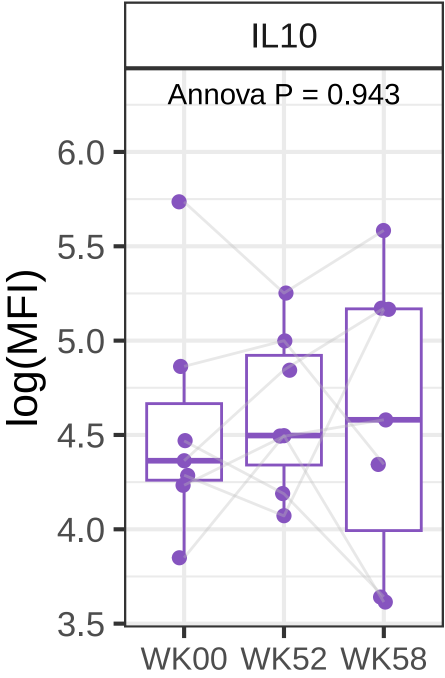
IL10
Annova P = 0.943
6.0
5.5
5.0
4.5
4.0
3.5
log(MFI)
WK00
WK52
WK58

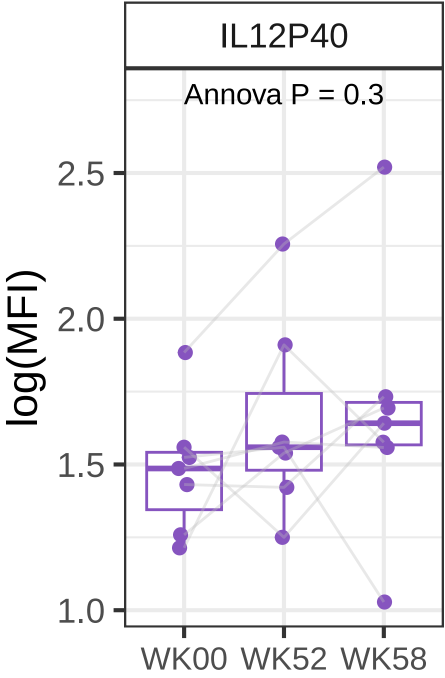
IL12P40
Annova P = 0.3
log(MFI)
2.5
2.0
1.5
1.0
WK00
WK52
WK58

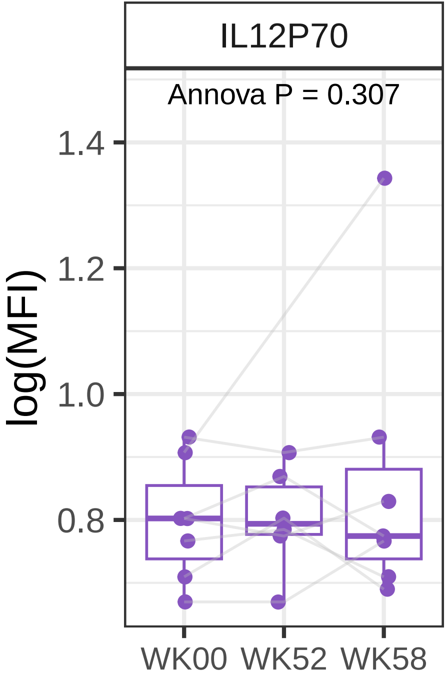
IL12P70
Annova P = 0.307
log(MFI)
1.4
1.2
1.0
0.8
WK00
WK52
WK58

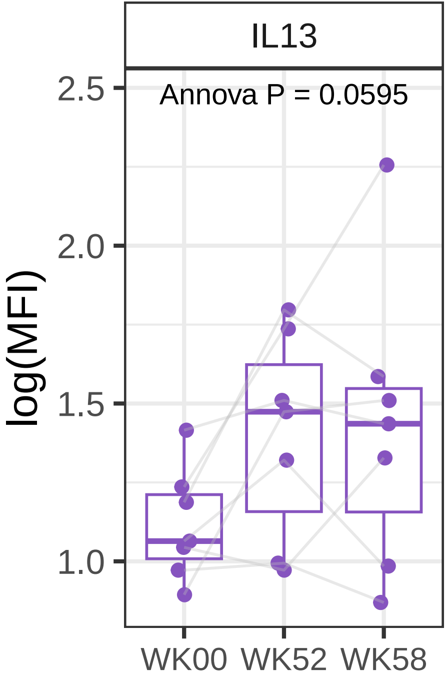

IL13
Annova P = 0.0595
log(MFI)
2.5
2.0
1.5
1.0
WK00
WK52
WK58

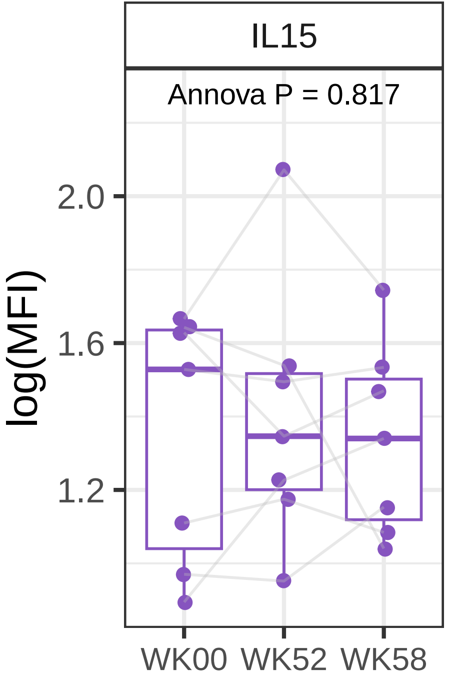
IL15
Annova P = 0.817
log(MFI)
2.0
1.6
1.2
WK00
WK52
WK58

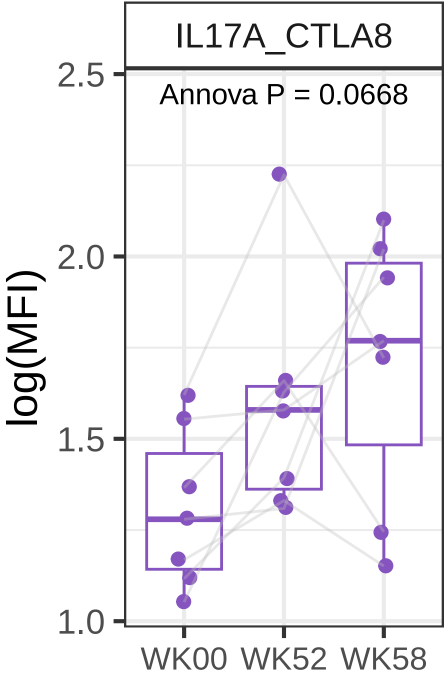
IL17A_CTLA8
Annova P = 0.0668
2.5
2.0
1.5
1.0
log(MFI)
WK00
WK52
WK58

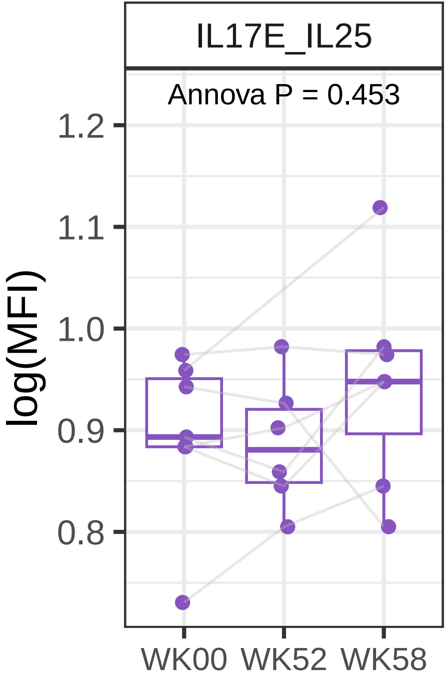
IL17E_IL25
Annova P = 0.453
log(MFI)
1.2
1.1
1.0
0.9
0.8
WK00
WK52
WK58

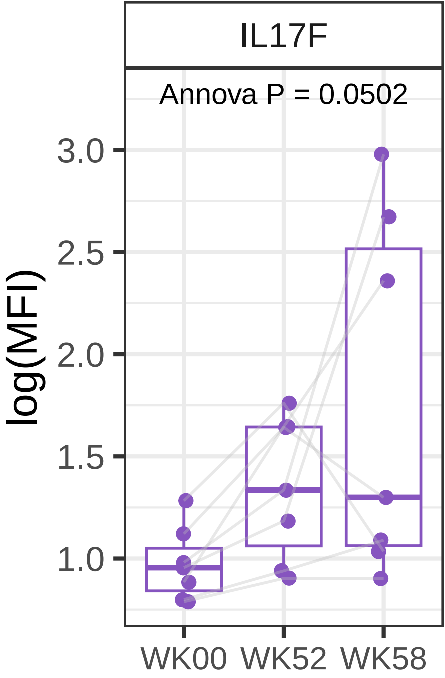
IL17F
Annova P = 0.0502
log(MFI)
3.0
2.5
2.0
1.5
1.0
WK00
WK52
WK58

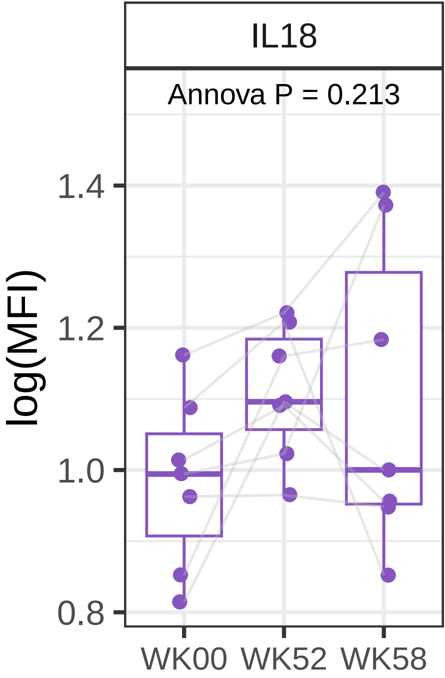

IL18
Annova P = 0.213
log(MFI)
1.4
1.2
1.0
0.8
WK00
WK52
WK58

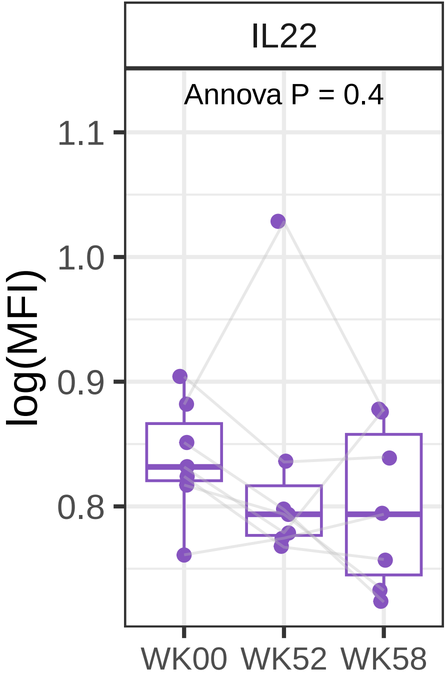
IL22
Annova P = 0.4
log(MFI)
1.1
1.0
0.9
0.8
WK00
WK52
WK58

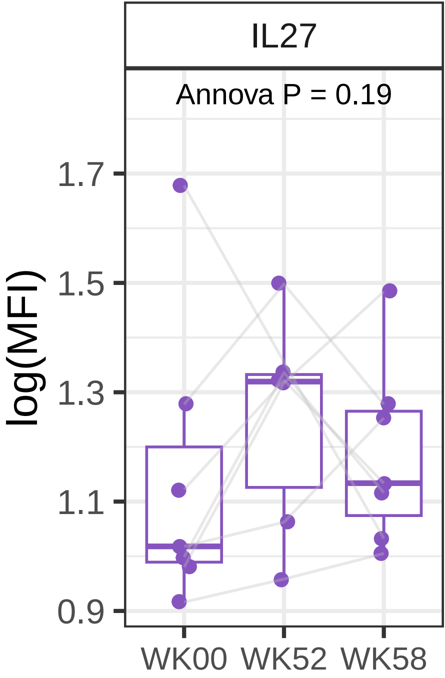

IL27
Annova P = 0.19
log(MFI)
1.7
1.5
1.3
1.1
0.9
WK00
WK52
WK58

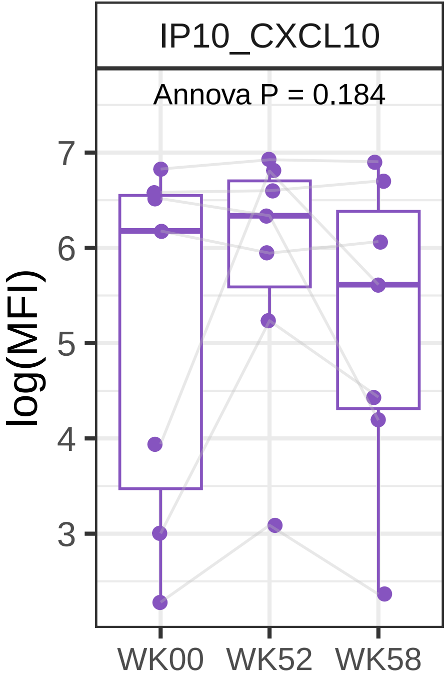
IP10_CXCL10
Annova P = 0.184
log(MFI)
7
6
5
4
3
WK00
WK52
WK58

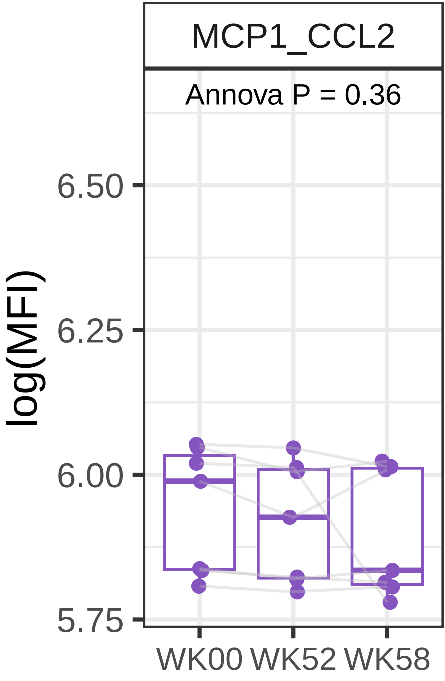
MCP1_CCL2
Annova P = 0.36
log(MFI)
6.50
6.25
6.00
5.75
WK00
WK52
WK58

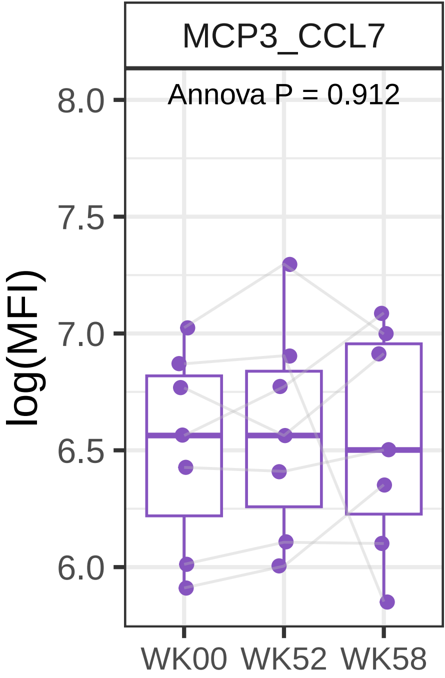
MCP3_CCL7
Annova P = 0.912
log(MFI)
8.0
7.5
7.0
6.5
6.0
WK00
WK52
WK58

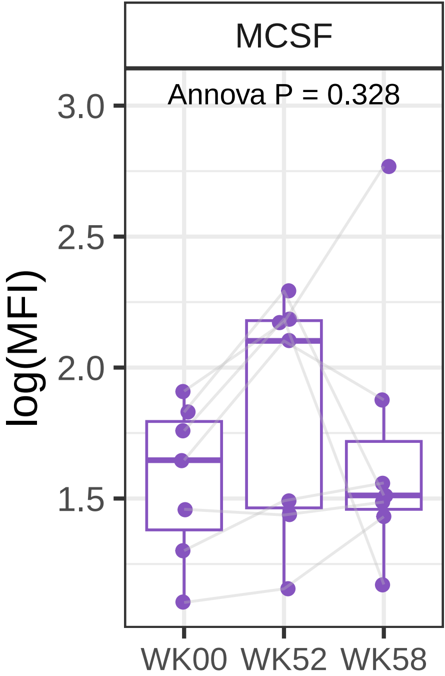
MCSF
Annova P = 0.328
log(MFI)
3.0
2.5
2.0
1.5
WK00
WK52
WK58

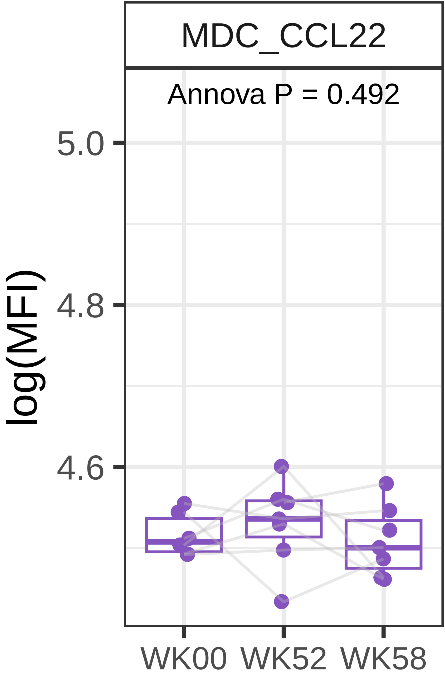
MDC_CCL22
Annova P = 0.492
log(MFI)
5.0
4.8
4.6
WK00
WK52
WK58

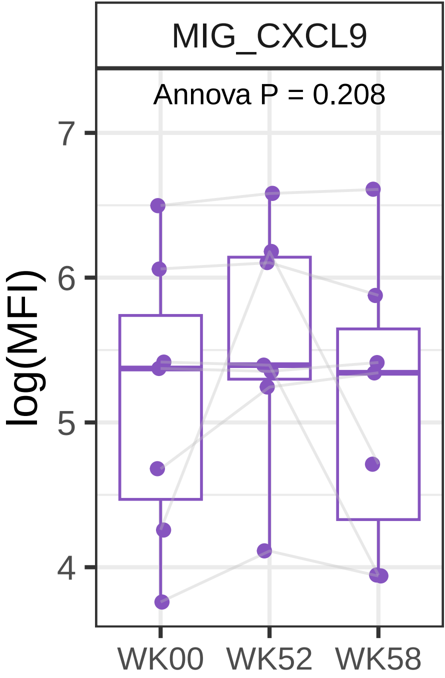
MIG_CXCL9
Annova P = 0.208
log(MFI)
7
6
5
4
WK00
WK52
WK58

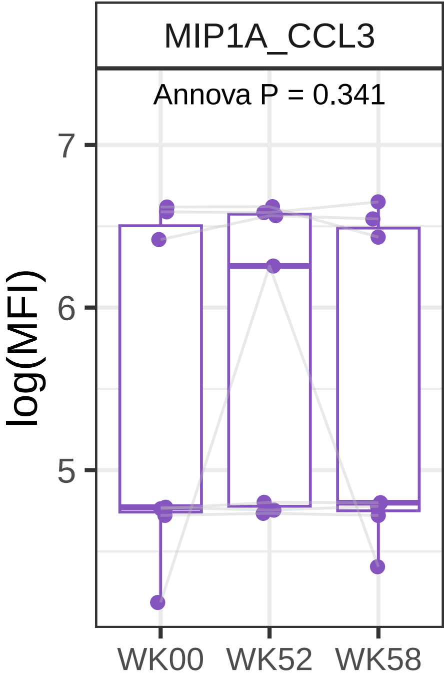
MIP1A_CCL3
Annova P = 0.341
log(MFI)
7
6
5
WK00
WK52
WK58

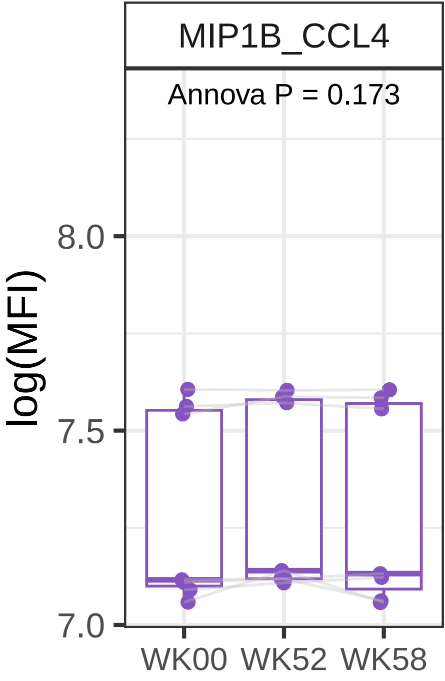
MIP1B_CCL4
Annova P = 0.173
log(MFI)
8.0
7.5
7.0
WK00
WK52
WK58

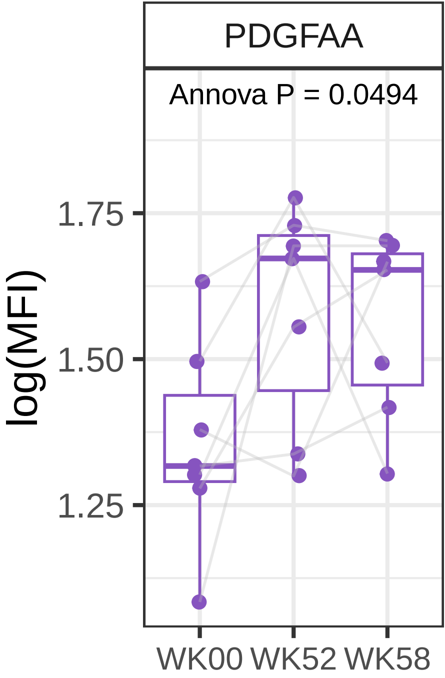
PDGFAA
Annova P = 0.0494
log(MFI)
1.75
1.50
1.25
WK00
WK52
WK58

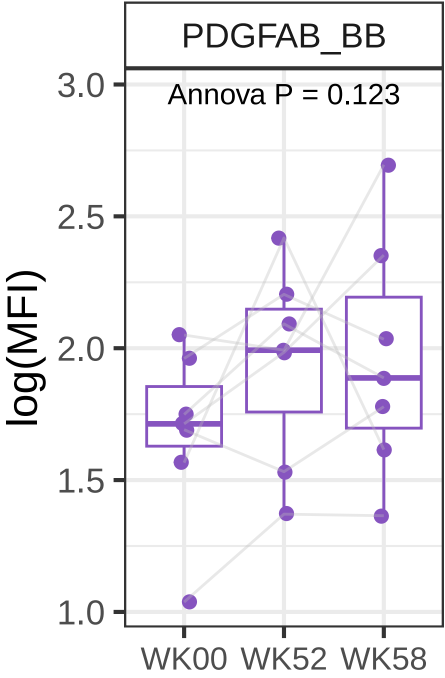
PDGFAB_BB
Annova P = 0.123
log(MFI)
3.0
2.5
2.0
1.5
1.0
WK00
WK52
WK58

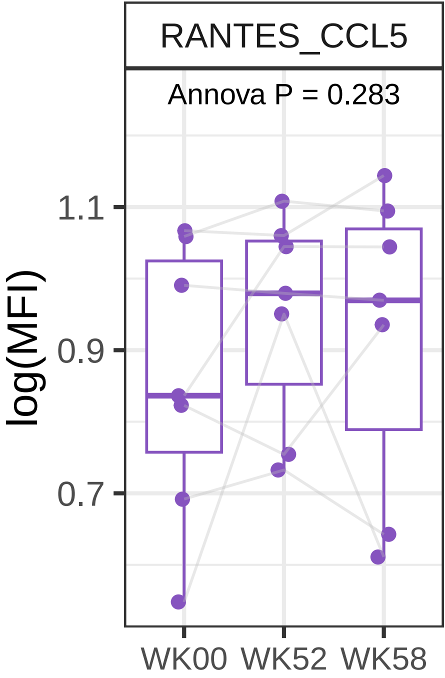
RANTES_CCL5
Annova P = 0.283
log(MFI)
1.1
0.9
0.7
WK00
WK52
WK58

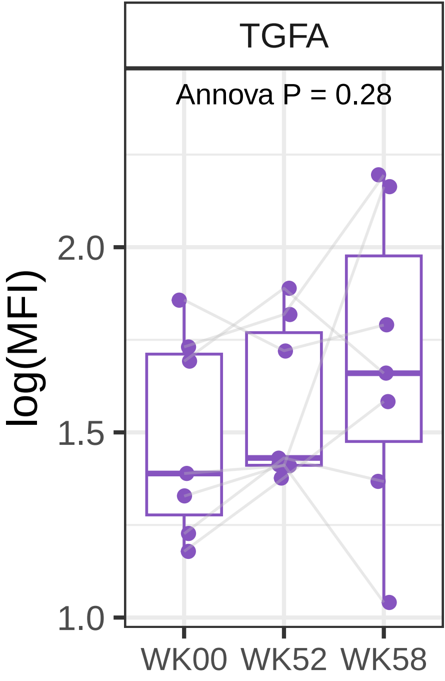
TGFA
Annova P = 0.28
log(MFI)
2.0
1.5
1.0
WK00
WK52
WK58

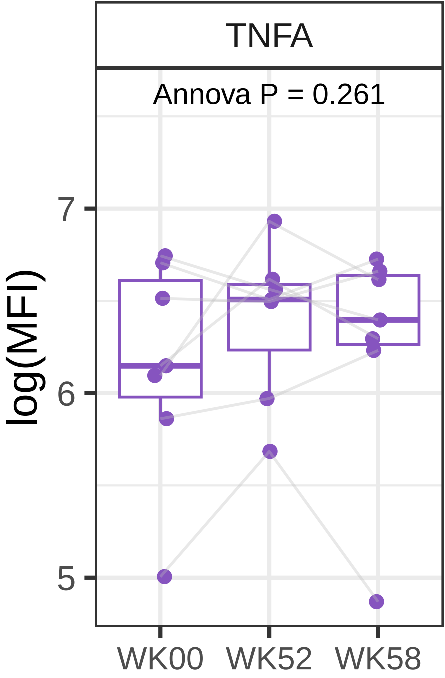
TNFA
Annova P = 0.261
log(MFI)
7
6
5
WK00
WK52
WK58

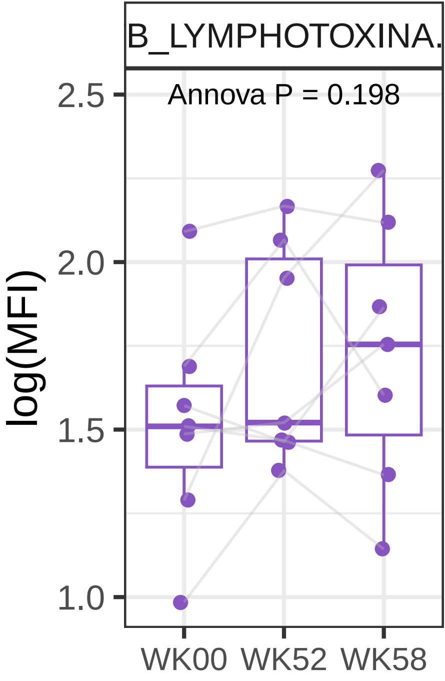

B_LYMPHOTOXINA.
Annova P = 0.198
log(MFI)
2.5
2.0
1.5
1.0
WK00
WK52
WK58

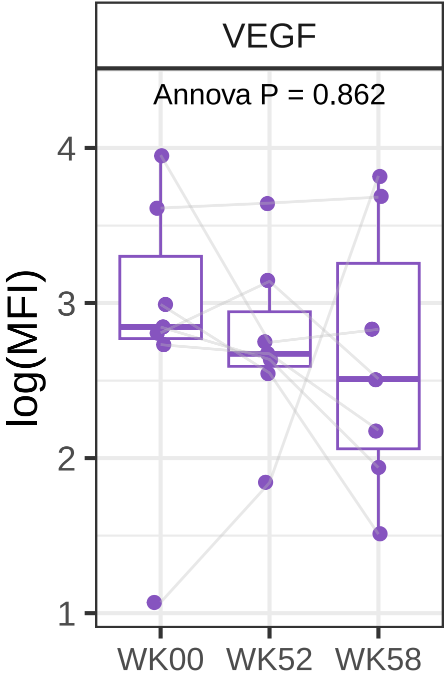
VEGF
Annova P = 0.862
log(MFI)
4
3
2
1
WK00
WK52
WK58
