# Supplementary figures and images for: Shrimp oral immunotherapy outcomes in the phase 2 clinical trial: MOTIF
Source: Front Allergy. 2025 Jul 22;6:1458131. doi: 10.3389/falgy.2025.1458131 (PMC12321884; doi:10.3389/falgy.2025.1458131)

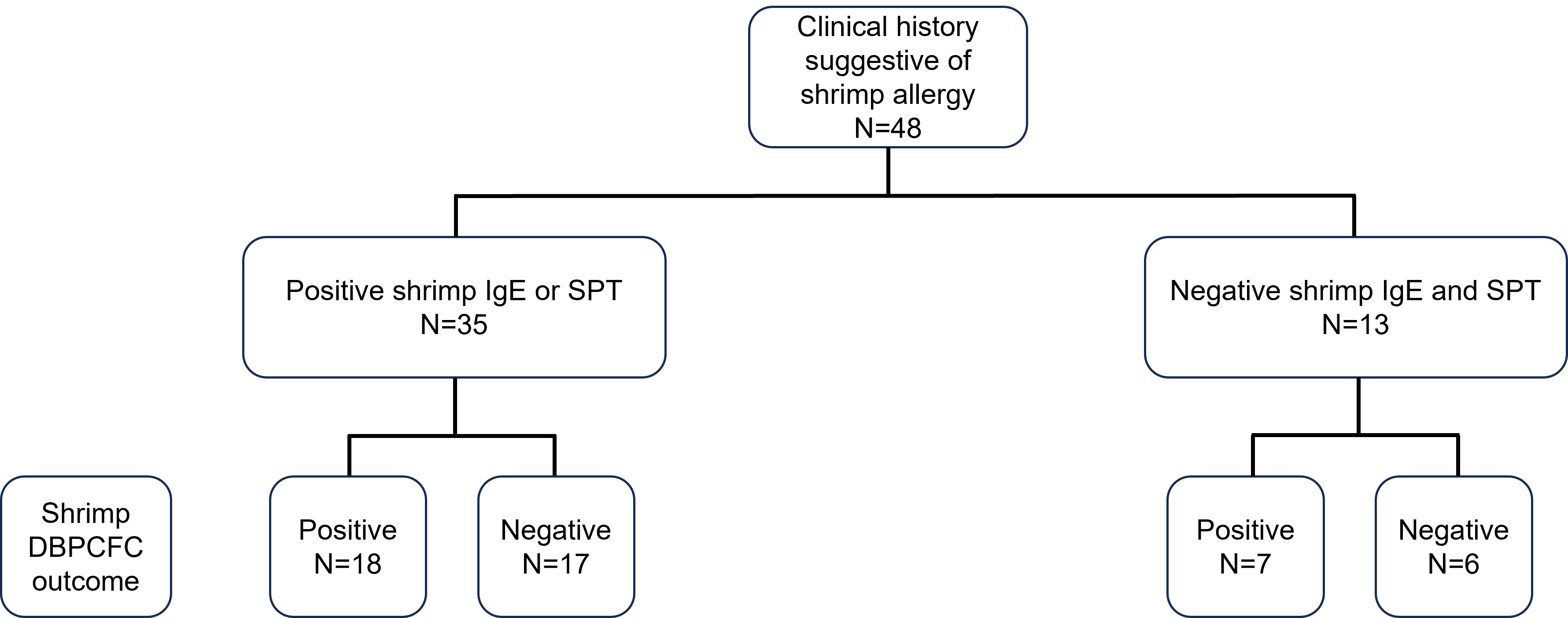

Supplement: Supplementary Figure S5 — Consort diagram for retrospective chart review. [file Image5.tif]

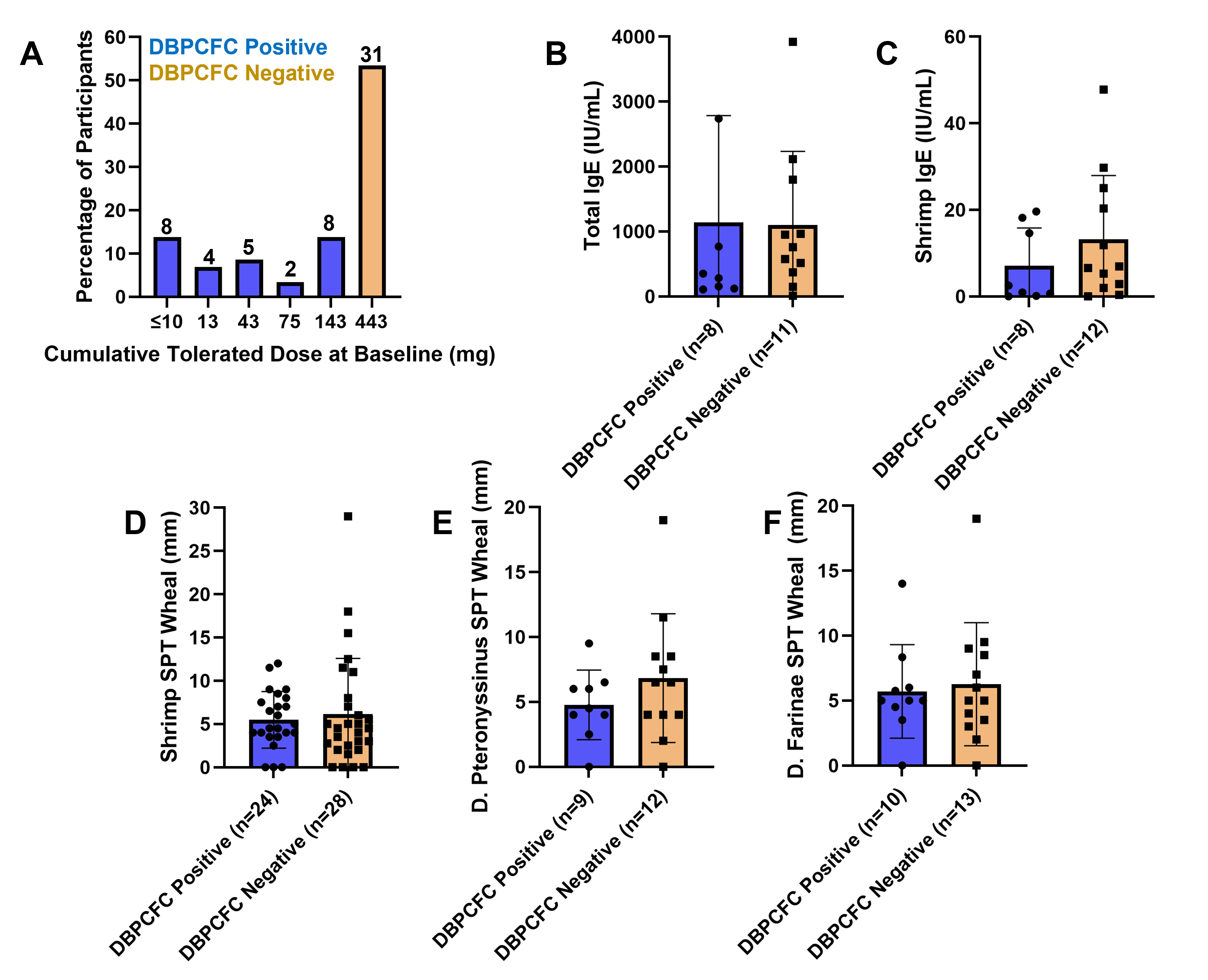

Supplement: Supplementary Figure S6 — Allergen sensitization in participants with positive or negative shrimp DBPCFC outcomes from retrospective chart review. [file Image6.tif]

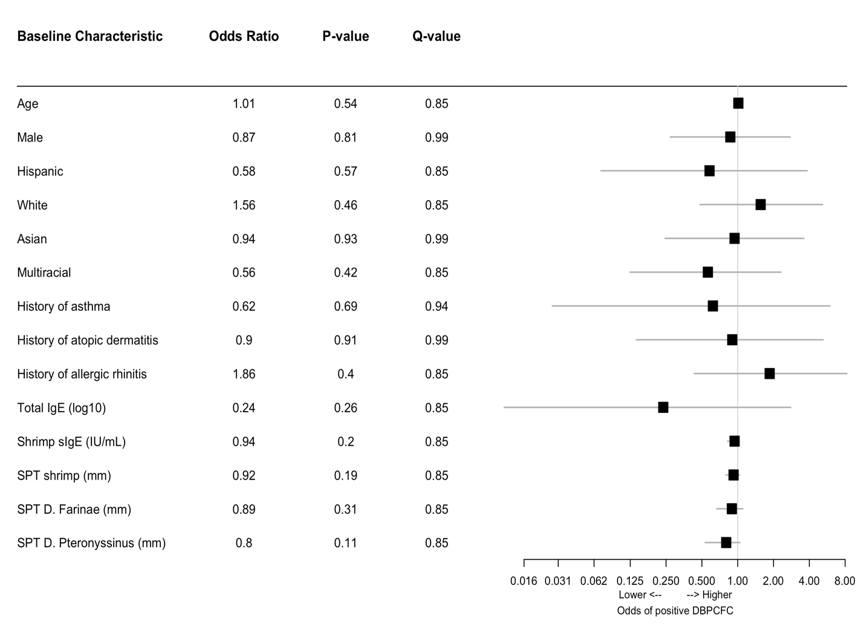

Supplement: Supplementary Figure S7 — Associations of baseline characteristics and the DBPCFC outcome. [file Image7.tif]

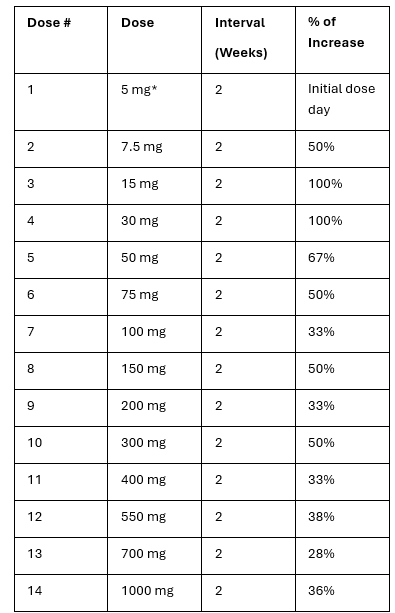

Supplement: Supplementary Table S1 — Dosing steps for shrimp OIT. [file Image8.tif]

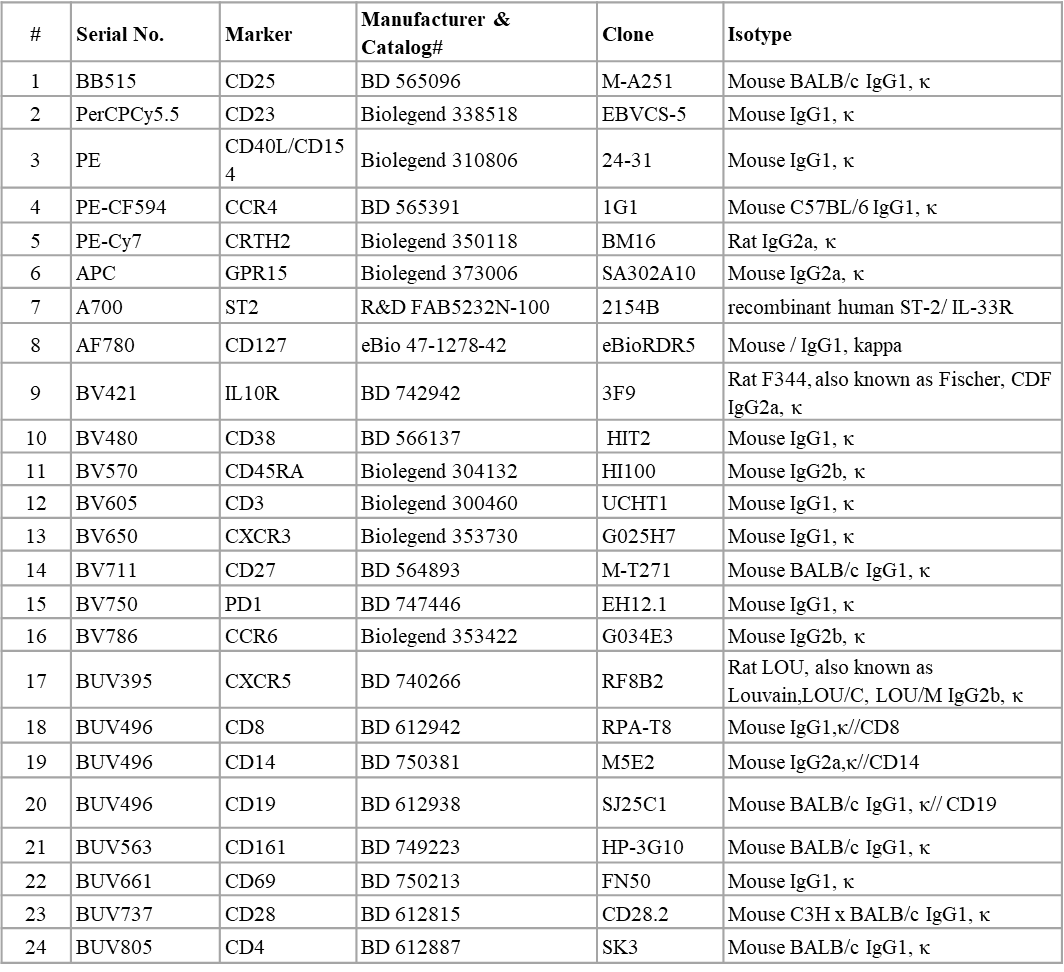

Supplement: Supplementary Table S2 — Staining panel for FACS analysis. [file Image9.tif]

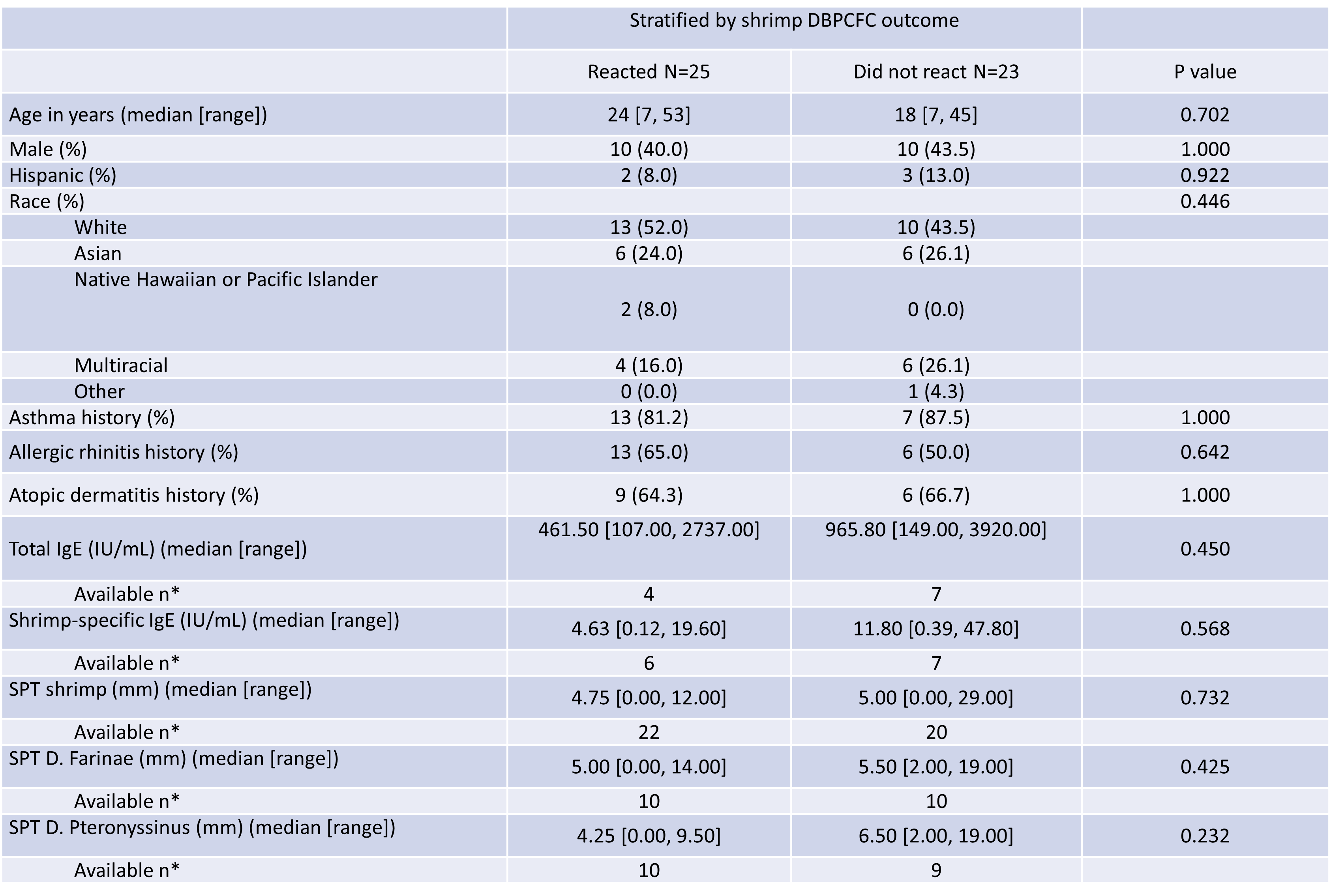

Supplement: Supplementary Table S3 — Screening demographics for participants screened for shrimp allergy for multiple studies. [file Image10.tif]
